# Single-cell RNA sequencing reveals the effects of chemotherapy on human pancreatic adenocarcinoma and its tumor microenvironment

**DOI:** 10.1038/s41467-023-36296-4

**Published:** 2023-02-13

**Authors:** Gregor Werba, Daniel Weissinger, Emily A. Kawaler, Ende Zhao, Despoina Kalfakakou, Surajit Dhara, Lidong Wang, Heather B. Lim, Grace Oh, Xiaohong Jing, Nina Beri, Lauren Khanna, Tamas Gonda, Paul Oberstein, Cristina Hajdu, Cynthia Loomis, Adriana Heguy, Mara H. Sherman, Amanda W. Lund, Theodore H. Welling, Igor Dolgalev, Aristotelis Tsirigos, Diane M. Simeone

**Affiliations:** 1 Department of Surgery, NYU Langone Health, New York, New York 10016 USA; 2 Perlmutter Cancer Center, NYU Langone Health, New York, New York 10016 USA; 3 Department of Medicine, NYU Langone Health, New York, New York 10016 USA; 4 Department of Pathology, NYU Langone Health, New York, New York 10016 USA; 5 Department of Cell, Developmental and Cancer Biology, Oregon Health Sciences University, Portland, Oregon 97239 USA; 6 Department of Dermatology, NYU Langone Health, New York, New York 10016 USA

**Keywords:** Pancreatic cancer, Cancer microenvironment, Pancreatic cancer, Bioinformatics, Cancer microenvironment

## Abstract

The tumor microenvironment (TME) in pancreatic ductal adenocarcinoma (PDAC) is a complex ecosystem that drives tumor progression; however, in-depth single cell characterization of the PDAC TME and its role in response to therapy is lacking. Here, we perform single-cell RNA sequencing on freshly collected human PDAC samples either before or after chemotherapy. Overall, we find a heterogeneous mixture of basal and classical cancer cell subtypes, along with distinct cancer-associated fibroblast and macrophage subpopulations. Strikingly, classical and basal-like cancer cells exhibit similar transcriptional responses to chemotherapy and do not demonstrate a shift towards a basal-like transcriptional program among treated samples. We observe decreased ligand-receptor interactions in treated samples, particularly between TIGIT on *CD8* + T cells and its receptor on cancer cells, and identify TIGIT as the major inhibitory checkpoint molecule of *CD8* + T cells. Our results suggest that chemotherapy profoundly impacts the PDAC TME and may promote resistance to immunotherapy.

## Abstract (web summary)

The role of therapy in shaping the tumor microenvironment in pancreatic ductal adenocarcinoma (PDAC) remains to be explored. Here, the authors perform single-cell RNA sequencing in PDAC samples before and after chemotherapy and suggest that chemotherapy may promote resistance to immunotherapy.

## Introduction

Pancreatic ductal adenocarcinoma (PDAC) is a highly lethal cancer with a five-year survival rate of approximately 11%^1^. With increasing incidence, it is projected to become the second leading cause of cancer-related deaths in the United States by 2030^2^. Current treatment options are limited. Only 15% to 20% of patients qualify for upfront surgery with curative intent; the remaining patients present with unresectable locally advanced disease or distant metastases. Nearly all patients receive adjuvant, neoadjuvant, or palliative chemotherapy^3^. Recent advances in immunomodulating drugs, such as checkpoint inhibitors, have yet to show encouraging results in PDAC^4,5^. A deeper understanding of pancreatic cancer biology is needed to develop better therapeutics and improve outcomes.

Although recent genomic and transcriptomic studies on bulk tumor samples identified key molecular drivers (notably *KRAS*, *TP53*, *CDKN2A*, and *SMAD4*) and cancer cell subtypes (basal and classical), these findings have yet to translate into treatment advances^6–9^. Recent investigations into the PDAC tumor microenvironment (TME) revealed that this complex ecosystem is a critical mediator of therapeutic resistance and tumor progression^10,11^. Single-cell RNA sequencing (scRNA-seq) has emerged as a valuable tool in precision medicine by enabling in-depth characterization of the TME and serial assessment on scant amounts of tissue^12–17^. While recent single-cell studies have provided insights into PDAC, there is an unmet need to characterize the effects of chemotherapy on the TME^18–22^.

For a comprehensive view of the PDAC TME, and its response to chemotherapy in a real-time clinical context, here we perform scRNA-seq on freshly collected tumor specimens from 27 PDAC patients. We observe significant heterogeneity in the malignant epithelial compartment, with most tumors displaying a mixture of basal-like and classical-like cells. Furthermore, we examine the composition of CAFs and discrete groups of immune cells in the TME and execute comparative analyses on naive and chemotherapy-treated samples, which we confirm in an external dataset. Our data show that chemotherapy profoundly alters the PDAC TME, inducing features consistent with tumor progression and refractoriness to immunotherapy by downregulation of inhibitory checkpoint molecules in CD8 + T cells.

## Results

### Single-cell analysis reveals the transcriptomic landscape in PDAC

**Fig. 1:**
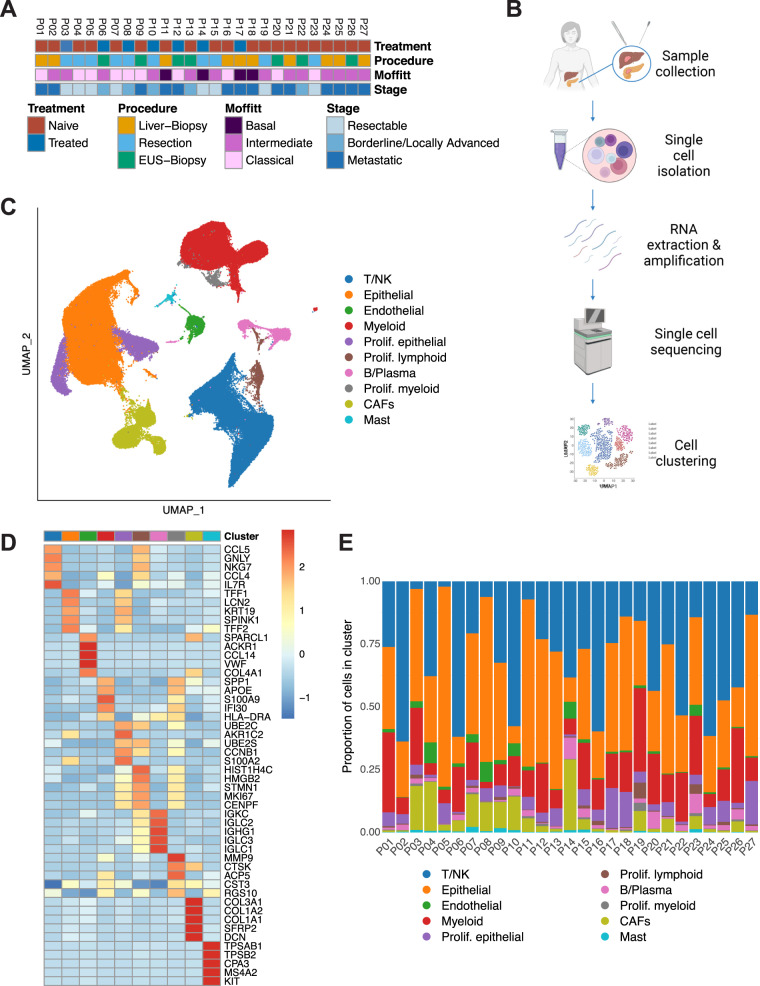
Single-cell analysis reveals the transcriptomic landscape in PDAC. **A** Clinicopathologic characteristics for each patient sample including treatment status, stage, Moffitt subtype, and procedure. EUS endoscopic ultrasound. **B** Workflow depicting sample acquisition, processing, and analysis. Designed with BioRender**©**. **C** UMAP, embedding of the expression profile of 139,446 cells that passed quality control from *n* = 27 patient samples. Distinct clusters are annotated and color-coded. CAF cancer-associated fibroblast, NK natural killer, UMAP uniform manifold approximation and projection. **D** Heatmap of the five most differentially expressed genes within each cluster. Colors correspond to the cluster colors in Fig. 1**C**. **E** Proportional distribution of each cell cluster by individual sample.

PDAC specimens were acquired via surgical resection of primary pancreatic lesions (*n* = 10) or by endoscopic/interventional radiology–guided biopsies of either primary pancreatic lesions (*n* = 7) or liver metastases (*n* = 10) (Fig. 1A, Supplementary Table 1). Of the 27 total patients, seven received chemotherapy (FOLFIRINOX-based *n* = 4, gemcitabine/abraxane-based *n* = 3) prior to tissue collection, while 20 patients had not been treated at the time of specimen acquisition (Fig. 1A, Supplementary Table 1). Evaluating the treated patients using the RECIST criteria, we found that two had stable disease, two partial response, three progressive disease, and one was nonevaluable (Supplementary Table 2). We prepared single-cell suspensions from tumor specimens and sequenced them using the 10x Genomics Chromium system (Fig. 1B, see Methods for details). Histopathological assessment was done on each tumor specimen to verify malignancy, and samples underwent mutational profiling (Supplementary Data 1, 2). After standard processing and quality filtering of the raw sequencing data (see Methods), a total of 139,446 cells were retained for analysis. Unsupervised, graph-based clustering and visualization via uniform manifold approximation and projection (UMAP) revealed ten distinct cell clusters that were annotated using canonical markers and gene expression profiles (Fig. 1C, D, Supplimentary Fig. 1A, Methods). Three major clusters were identified as epithelial, T/natural killer (NK), and myeloid cells. We further identified CAFs, endothelial cells, B/plasma cells, and a small mast cell population among our clusters. The proportional distribution of these major cell types was similar across all groups, with the exception of biopsies from liver metastases and primary PDAC which had quantitatively lower CAFs and endothelial cells than resection samples. We also identified an elevated amount of proliferating cancer cells in liver biopsies (Fig. 1E; Supplementary Fig. 1B). To ensure that tissue processing did not significantly change gene expression in our samples, we applied hypoxia and dissociation signatures^23,24^ to our dataset. This analysis showed that all scSeq samples contained very few hypoxic cells, with no significant differences between procedures (Supplimentary Fig. 1C), and demonstrated low dissociation signatures independent of procedure (Supplimentary Fig. 1D).

### The epithelial compartment reveals heterogeneous malignant subtype composition

**Fig. 2:**
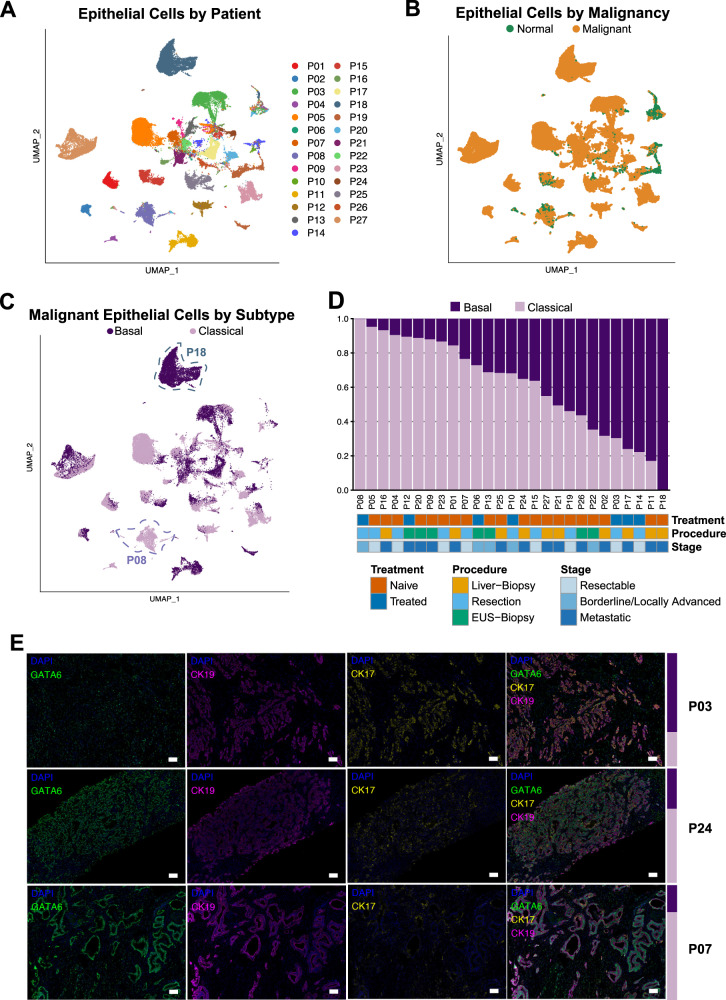
The epithelial compartment reveals heterogeneous malignant subtype composition. **A** Non-batch corrected UMAP of all epithelial cells, showing clustering by patient sample of most epithelial cells and clustering of some epithelial cells across samples. UMAP uniform manifold approximation and projection. **B** Non-batch corrected UMAP of malignant and normal epithelial cells as determined by InferCNV. **C** UMAP embedding of malignant epithelial cells labeled with Moffitt subtypes. Exemplary samples high in basal (P18) and classical (P08) cells are annotated. **D** Proportion of cells of each Moffitt subtype by sample. EUS endoscopic ultrasound. **E** Representative (*n* = 27) images of multiplex immunofluorescence across the subtype spectrum: higher in basal (upper panel, P03), mixed (middle panel, P24), and higher in classical (lower panel, P07) subtype tumors. Basal-to-classical ratio for each sample by scRNA-seq transcriptional analysis is shown on the right as a colored bar (dark = basal, light = classical). Channels (always including DAPI (blue)): GATA6 (green), CK19 (cytokeratin 19) (violet), CK17 (cytokeratin 17) (yellow), and merged. Scale bar = 100 μm.

The epithelial compartment evinced sample-specific differences while the mesenchymal and immune compartments clustered together across all samples (Supplimentary Fig. 2A). This suggests that non-epithelial cells of the PDAC TME share more common transcriptional signatures across all patients than their malignant counterparts within the same tumor. Since some epithelial cells also clustered across samples, we further assessed the proportion of malignant cells in the total epithelial compartment (Fig. 2A). To this end, we performed copy number variation (CNV) analysis. The vast majority of epithelial cells showed several CNV events, indicating malignancy and distinguishing them from normal epithelial cells (Fig. 2B, Supplimentary Fig. 2B). We further classified malignant cells as either basal or classical based on their Moffitt subtype signature expression^7^. (Any signature gene sets referenced in this paper can be found in Supplimentary Data 3). All samples but one were comprised of a mixture of basal and classical cancer cells in varying proportions (Fig. 2C, D). Tumor subtype composition did not correlate with the amount of any major non-malignant cell types (Supplimentary Fig. 2C).

We validated the Moffitt transcriptional subtypes on formalin-fixed, paraffin-embedded tissue (FFPE) sections using multiplex immunohistochemistry (IHC). We used the canonical markers cytokeratin 17 (CK17) for the basal subtype, GATA6 for the classical subtype, and cytokeratin 19 (CK19) as a pan-cancer cell marker. Representative samples with a higher percentage of basal or classical transcriptional subtypes were strongly positive for CK17 or GATA6, respectively (Fig. 2E, upper and lower panels), while a sample with a more even mixture of both subtypes showed an interspersed, more equal expression of GATA6 and CK17 (Fig. 2E, middle panels). Another patient sample with a mixture of both subtypes showed two distinct growth patterns with a CK17^high^ region next to a GATA6^high^ region (Supplimentary Fig. 2D). In general, GATA6 staining was more omnipresent, and CK17^high^ cells often stained positive for GATA6. Of note, most samples included cells staining positive for both CK17 and GATA6 (Supplimentary Fig. 2E), consistent with a previous report that hypothesized an intermediate cell state^25^.

To test this intermediate cell state hypothesis in our dataset, we applied the basal, classical, and intermediate gene signatures from that report, which were derived from treatment-naive liver metastases, to malignant cells from our treatment-naive PDAC liver metastatic samples. Although their basal and classical signatures matched closely with ours, we did not see clear evidence of the intermediate signature (Supplimentary Fig. 2F). We also assessed the bulk RNA-derived subtype classifications of Collisson, Bailey and Puleo^26–28^. In our dataset, the Moffitt-basal signature showed overlap with the Collisson-quasi-mesenchymal and Puleo-pure-basal-like, while the Bailey-squamous signature did not show a strong correlation with the basal signatures. Similarly, the Moffitt-classical signature overlapped with the Collisson-classical and Puleo-pure-classical signatures, while the Bailey-progenitor signature did not show a strong correlation with the classical signatures. The less commonly found subtype signatures of Bailey (ADEX and immunogenic), Collisson (exocrine-like), and Puleo (desmoplastic) did not show strong expression in our dataset of CNV-confirmed cancer cells, suggesting that these might be derived from non-epithelial cells within tumor samples (Supplimentary Fig. 2G, H). These findings are supported by a TCGA publication from 2017 which examined sample purity and subtype classifications^9^. Further characterization of basal and classical cells by differential gene expression showed typical basal and classical genes upregulated in the respective subtypes (Supplimentary Fig. 2I). Pathway enrichment analysis revealed enrichment of EMT, angiogenesis, and extracellular matrix (ECM) interaction—features related to migration and invasion—in basal cells, while classical cells were enriched for metabolic pathways, especially lipid metabolism (Supplimentary Fig. 2J). These results emphasize the heterogeneity of malignant epithelial cells in PDAC and suggest that single-marker analysis may be insufficient to accurately identify cancer cell subtypes within a patient’s tumor.

### Charting the CAF landscape in the PDAC tumor microenvironment

**Fig. 3:**
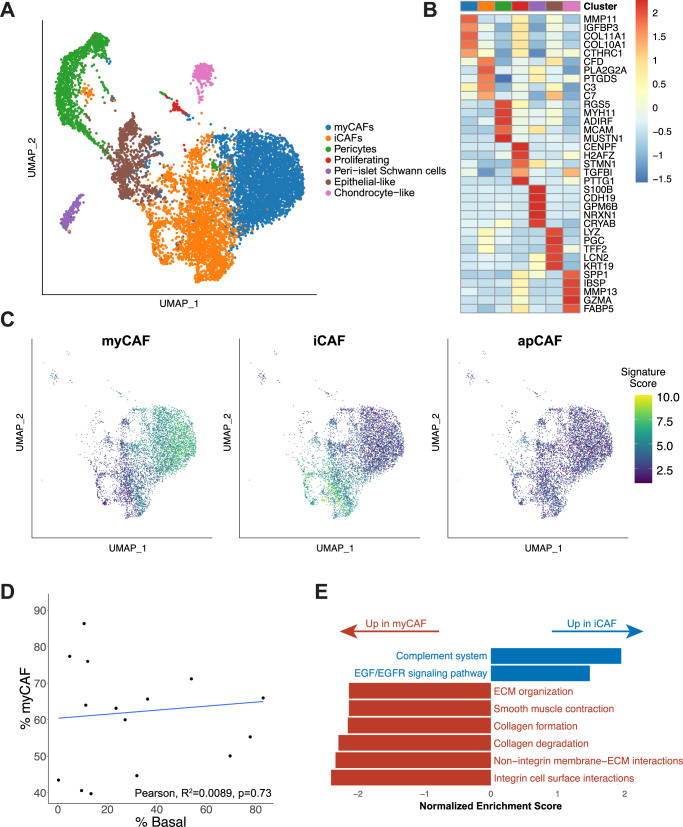
Charting the CAF landscape in the PDAC tumor microenvironment. **A** UMAP overview of the mesenchymal compartment. CAF cancer-associated fibroblast, iCAF inflammatory CAF, myCAF myofibroblastic CAF, UMAP uniform manifold approximation and projection. **B** Heatmap of the five most differentially expressed genes within each cluster. Colors correspond to the cluster colors in panel (**A**, **C**). iCAF, myCAF, and apCAF signatures from Elyada et al.^19^ overlaid on the UMAP embedding of our mesenchymal compartment. The iCAF and myCAF signatures are derived from human fibroblasts; the apCAF signature is derived from KPC mouse fibroblasts. apCAF antigen-presenting CAF. **D** Assessment of correlation (two-sided Pearson correlation) between myCAF/iCAF composition and basal/classical composition among our samples (*n* = 16). Samples are plotted as a function of the percentage of their CAF cells that are myCAFs and the percentage of their malignant epithelial cells that are basal. Samples with fewer than 40 total CAF cells were excluded; all but one of these samples were biopsies, which were not expected to pick up many mesenchymal cells. **E** Selected gene set enrichment analysis results from a comparison between the cells in the iCAF and myCAF compartments. ECM extracellular matrix.

We next analyzed the mesenchymal compartment of the PDAC TME. Mesenchymal cell reclustering revealed seven distinct clusters (Fig. 3A). The two main clusters consisted of myofibroblastic CAFs (myCAFs) and inflammatory CAFs (iCAFs) (Fig. 3A, B)^29^. We did not find a strong antigen-presenting CAF (apCAF) signature (Fig. 3C). Notably, the apCAF signature was originally derived from mouse CAFs and may not translate to the human CAFs in our cohort. As apCAF features in human CAFs have been linked to co-expression of *CD74* and human leukocyte antigen (HLA) molecules, we checked for expression of these genes in our CAF populations. A small number of cells in both the iCAF and myCAF subpopulations co-expressed CD74 and HLA genes (Fig. 3C, Supplementary Fig. 3A)^19^. Other clusters were identified as pericytes and Schwann cells, while a single sample provided a cluster of chondrocyte-like cells. Additionally, we detected a small proportion of cells expressing epithelial markers (Fig. 3A, B). We ruled out these cells being cancer cells undergoing EMT due to a lack of CNVs, low expression of EMT-related transcription factors (TFs) *SNAIL1* and *TWIST1*, and low values of an EMT-TF independent partial EMT (pEMT) program signature^30^ (Supplimentary Fig. 3B). These cells were also not consistent with mesothelial cells, as mesothelin (*MSLN*) and other markers for mesothelial cells were minimally expressed; we therefore labeled this cluster “epithelial-like” (Supplimentary Fig. 3C).

The myCAF to iCAF ratio varied across samples, while the proportional distribution of both subpopulations in relation to Moffitt subtype distribution was not significantly different (Fig. 3D). In myCAFs, *SDC1*^31^, a gene linked to CAF-mediated regulation of breast cancer cell migration, was upregulated, while in iCAFs, *CCL19*^32^, a gene linked to cytotoxic T cell recruitment by CAFs in lung cancer, was highly expressed (Supplimentary Fig. 3D, Supplimentary Data 4). Additionally, iCAFs showed enrichment for genes involved in the complement cascade and EGF signaling, whereas myCAFs demonstrated enrichment for ECM organization and smooth muscle contraction, supporting the notion of CAF heterogeneity and distinct functional characteristics of CAFs in the TME (Fig. 3E).

### Charting the T/NK landscape in the PDAC tumor microenvironment

**Fig. 4:**
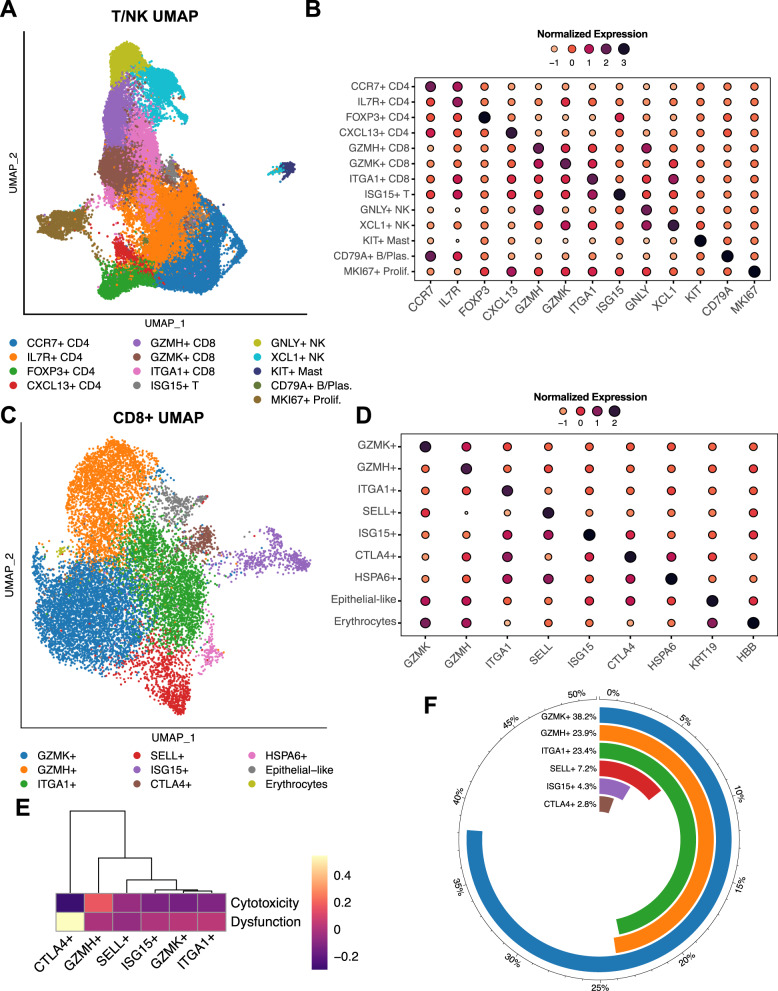
Charting the T/NK landscape in the PDAC tumor microenvironment. **A** UMAP overview of the T/NK compartment. NK natural killer, UMAP uniform manifold approximation and projection. **B** The most differentially expressed gene within each T/NK cluster. **C** UMAP of the more granular *CD8* + T cell reclustering reveals additional subpopulations. **D** The most differentially expressed gene within each *CD8* + T cell cluster. **E** Hierarchical clustering of the *CD8* + T cell subsets based on dysfunction and cytotoxicity scores. **F** Composition of the *CD8* + T cell subset (excluding the two non-T cell clusters and the single-patient cluster).

To better characterize tumor-infiltrating lymphocytes, we analyzed the T/NK population and identified 13 distinct clusters (Fig. 4A). *CD4* + T cells clustered into four distinct subtypes: (i) *CCR7*+ *CD4*+ (high expression of naïve/central memory markers), (ii) *IL7R* + *CD4* + (high expression of activation markers), (iii) *FOXP*3+ *CD*4+ (high expression of regulatory T cell genes), and (iv) *CXCL13* + *CD4* + (high expression of follicular helper genes) (Fig. 4B). *CD8* + T cells clustered into three main subpopulations: (i) *GZMH* + *CD8* + (high in cytotoxic gene expression), (ii) *GZMK*+ *CD8*+ (expressing effector memory genes), and (iii) *ITGA1* + *CD8* + (expression of tissue residency and exhaustion markers). We also identified a small *ISG15* + cluster, shared between *CD4* + and *CD8* + T cells, with high interferon (IFN)-related gene expression. NK cells separated into a *GNLY*+ cluster highly expressing cytotoxic genes and an *XCL1* + cluster with lower cytotoxic gene expression and higher expression of genes previously linked to tissue-resident NK cells^33,34^. Smaller clusters were identified as mast cells and plasma cells (Fig. 4A, B). T/NK subpopulations were heterogeneously distributed across samples and did not correlate with basal/classical subtyping (Supplimentary Fig. 4A, B).

Because our initial T/NK clustering did not identify distinct exhausted T cell subpopulations, we refined the resolution of the *CD8* + T cell clustering (Fig. 4C). At this finer granularity, we discerned a small *CTLA4* + *CD8* + T cell cluster with high levels of exhaustion markers (*CTLA4, HAVCR2, LAG3, TIGIT*). Additional distinct CD8 + T cell clusters included (i) *GZMK* + *CD8* + (high expression of effector memory genes), (ii) *GZMH* + *CD8* + (high expression of cytotoxic genes), (iii) *ITGA1* + *CD8* + (high expression of tissue residency genes), (iv) *SELL* + *CD8* + (expression of naive/central memory genes), and (v) *ISG15* + *CD8* + (expressing IFN activation genes) (Fig. 4C, D). We also found an epithelial-like cluster, an erythrocyte-like cluster, and an *HSPA6* + cluster; the latter was mainly derived from one patient and did not show other distinct *CD8* + subpopulation features (Fig. 4C, D).

To further assess exhaustion in our *CD8* + T cell subpopulations, we scored each subset on a dysfunction and cytotoxicity scale^35^. As expected, *GZMH* + *CD8* + T cells scored highest in cytotoxicity, and *CTLA4* + *CD8* + T cells highest in exhaustion (Fig. 4E). Except for *CTLA4* + *CD8* + T cells, the exhaustion state was similar between subsets (Fig. 4E). Exhausted *CD8* + T cells represented only 2.8% of all *CD8* + T cells (Fig. 4F). Moreover, exhausted T cells, along with most other *CD8* + T cell subsets, were heterogeneously distributed across samples, independent of the Moffitt subtype composition of their epithelial compartments (Supplimentary Fig. 4C). Of note, *ITGA1* + *CD8* + T cells were the only subpopulation that was significantly negatively correlated with the basal subtype (Supplimentary Fig. 4D).

### C1QC+ and SPP1+ TAMs comprise two distinct subpopulations within the myeloid cell compartment

**Fig. 5:**
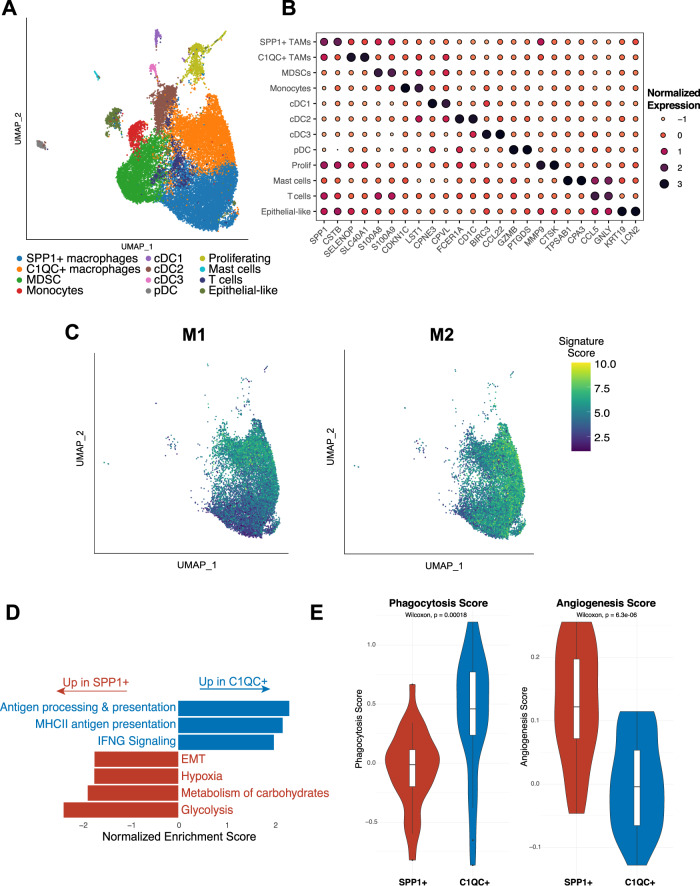
*C1QC* + and SPP*1* + TAMs comprise two distinct subpopulations within the myeloid cell compartment. **A** UMAP overview of the myeloid compartment. DC dendritic cell, MDSC myeloid-derived suppressor cell, UMAP uniform manifold approximation and projection. **B** For each myeloid cluster, the two genes most differentially expressed between that cluster and the rest of the myeloid compartment. TAM, tumor-associated macrophages. **C** M1 (left) and M2 (right) polarization signatures in the macrophage populations. **D** Selected gene set enrichment analysis results from a comparison between *SPP1* + and *C1QC* + TAMs. EMT, epithelial-mesenchymal transition. **E** Phagocytosis and angiogenesis scores for SPP*1* + and *C1QC* + TAMs. One sample with fewer than 40 total TAM cells was excluded. (*n* = 26 samples; two-sided Wilcoxon rank-sum test; box plots centered around the median with hinges at 1^st^ and 3^rd^ quartiles and whiskers from hinge to max value or 1.5*IQR, whichever is smallest).

Myeloid cells are an important component of the larger TME. We identified twelve distinct myeloid clusters (Fig. 5A). TAMs, representing the majority of myeloid cells, clustered into SPP*1* + and *C1QC* + subpopulations (Fig. 5A, B). We also identified myeloid-derived suppressor cells (MDSCs), monocytes, and dendritic cells (DCs), which further clustered into conventional DCs 1-3 (cDC1-3) and plasmacytoid DCs (pDCs) (Fig. 5A, B). We did not detect any trends in myeloid composition across samples or in relation to Moffitt subtypes (Supplimentary Fig. 5A, B).

To better understand what distinguishes SPP*1* + and *C1QC* + TAMs, we further characterized these two populations. Historically, TAMs have been classified as either M1 (pro-inflammatory) or M2 (pro-tumorigenic), so we assessed M1 and M2 signature expression in our TAMs^13^. While *C1QC* + TAMs showed higher M1 signature expression than SPP*1* + TAMs, both SPP*1* + and *C1QC* + subsets displayed strong M2 signature expression and therefore could not be clearly distinguished based on the M1/M2 classification (Fig. 5C). SPP*1* + TAMs showed increased expression of *S100A8* and *S100A9*, genes previously linked to immunosuppressive macrophages^36^, while *C1QC* + TAMs had increased expression of *HLA-DRB5* and *CXCL9*, which is involved in the anticancer immune response^37^ (Supplimentary Fig. 5C, Supplimentary Data 4). In general, SPP*1* + TAMs were enriched for genes involved in EMT, metabolism of carbohydrates, and hypoxia, while *C1QC* + TAMs showed enrichment for IFN signaling and antigen presentation (Fig. 5D). Consistent with the gene set enrichment analysis (GSEA) results and a recent colon cancer study^38^, SPP*1* + TAMs were enriched for angiogenesis-related genes and *C1QC* + TAMs for phagocytosis (Fig. 5E).

To estimate whether the TAM subtype may affect overall survival in PDAC, we evaluated SPP*1* + and *C1QC* + TAM signature expression in the Cancer Genome Atlas (TCGA) PDAC bulk tumor dataset^9^. While a similar analysis of the colorectal cancer (CRC) TCGA dataset implied worse overall survival for patients with a SPP*1* + ^high^ /*C1QC* + ^low^ TAM combination^38^, we found that estimated TAM composition did not correlate with overall survival in the TCGA PDAC data (Supplimentary Fig. 5D).

### Chemotherapy induces transcriptional changes in cancer cells independent of subtype

To analyze the effect of chemotherapy on the PDAC TME, we focused on primary pancreatic tumor specimens collected before treatment (treatment-naive, *n* = 11) and after treatment (*n* = 6). Specimens collected from liver biopsies were excluded from this part of the analysis in order to minimize potential confounding effects of mixing tumors from multiple sites. We also obtained an external dataset from Cui Zhou et al.^39^ to use as a confirmatory set to validate our findings; this dataset contains primary pancreatic tumor specimens collected before treatment (*n* = 7) and after treatment (*n* = 14).

**Fig. 6:**
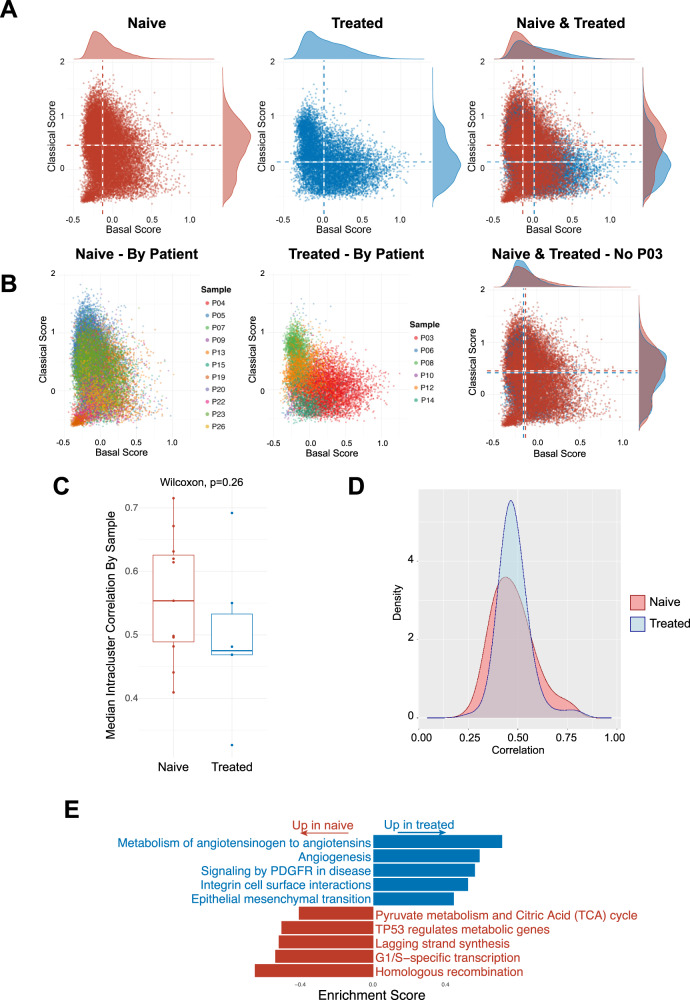
Chemotherapy treatment induces transcriptional changes in cancer cells independent of subtype. **A** Basal and classical signature expression scatterplot of cancer cells (treated, naive and combined). **B** Basal and classical signature expression scatterplot of cancer cells (treated and naïve by patient, combined by treatment); sample P03 has been removed from the combined plot. **C** Box plot showing the median intracluster correlation of cancer cells by sample for treated (*n* = 6) and naive (*n* = 11) groups (two-sided Wilcoxon rank-sum test, *p* = 0.26; box plots centered around the median with hinges at 1^st^ and 3^rd^ quartiles and whiskers from hinge to max value or 1.5*IQR, whichever is smallest). **D** Density plots of cancer cell correlation within samples for treated and untreated groups. **E** Gene set enrichment analysis of overall cancer cells, treated and untreated.

As our results have shown that most samples are a mixture of basal and classical cancer cells, which could imply subtype plasticity, we investigated the effect of chemotherapy on subtype distribution. Among the top differentially expressed genes was *TFF3*, part of the classical subtype signature^7,27^, which was significantly higher in naive samples than treated samples (Supplimentary Fig. 6A, Supplimentary Data 4). To validate the differences in RNA expression we performed IHC analyses of TFF3 in select corresponding tissue slides (Supplimentary Fig. 6B, C). Since a single marker might not be sufficient to characterize samples as basal or classical, we used representative genes for both Moffitt subtypes^7^ to create a basal and classical signature score which allowed us to place each cell on a basal-classical spectrum rather than assigning discrete phenotypes. Overall, treated samples did not show a shift towards a more basal-like expression pattern (Fig. 6A), although the treated sample P03 was distinctly more basal than any other sample (Fig. 6B). P03 was, notably, the only treated sample with poorly differentiated histology. Generally, cancer cells clustered by sample on this subtype spectrum, highlighting intertumoral heterogeneity (Fig. 6B). We saw this behavior in our confirmatory dataset (Supplimentary Fig. 6D, E), which also contained one distinctly basal treated patient; when this patient was removed, the median classical score in the treated samples was actually higher than in the untreated samples. Looking beyond Moffitt signature genes, we also measured the heterogeneity of a sample’s expression profile by calculating the correlations between every pair of its cancer cells. By this metric, treated and naive samples displayed no difference in intratumor heterogeneity, either per sample (Fig. 6C) or per cell (Fig. 6D).

To identify cell states, we used GSEA to check for common gene expression patterns of cancer cells in each sample. Here, cancer cells from treated samples were enriched in pathways related to angiogenesis and EMT, processes that can contribute to tumor recurrence and progression, as compared to naive samples (Fig. 6E). In the confirmatory dataset, we continued to see pathways related to angiogenesis and EMT enriched in the treated samples (Supplimentary Fig. 6F). Analysis of CAF and TAM subpopulation distributions between naive and treated groups yielded no significant changes (Supplimentary Fig. 6G).

### Chemotherapy reduces expression of inhibitory checkpoint molecules and ligand-receptor interactions in PDAC

**Fig. 7:**
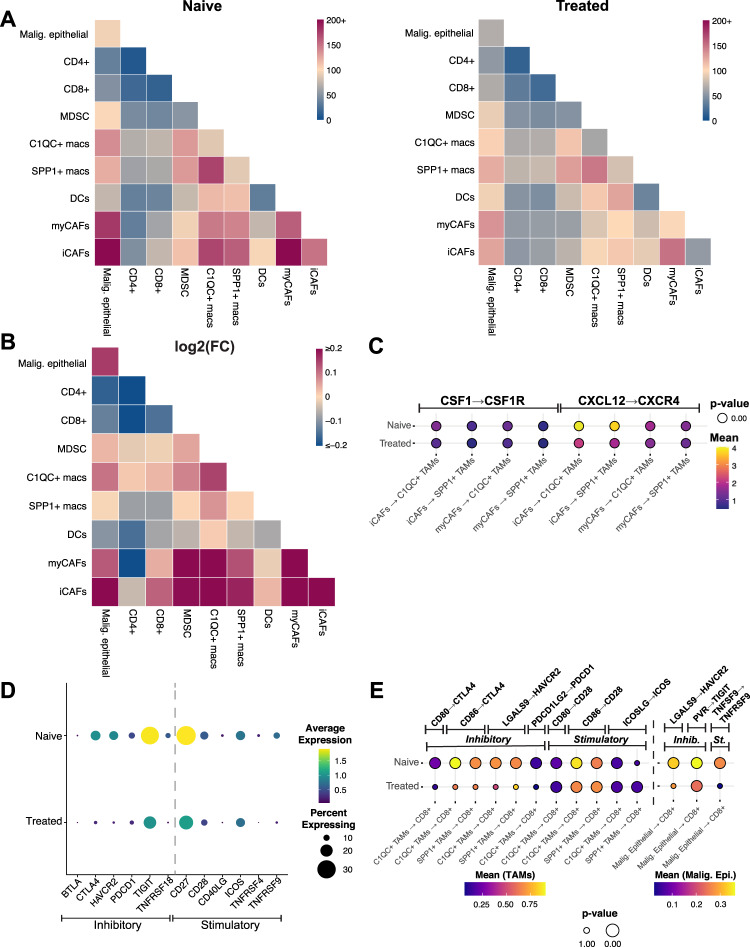
Chemotherapy reduces expression of inhibitory checkpoint molecules and ligand-receptor interactions in PDAC. **A** Heatmap displaying an overview of all inferred LRIs between displayed cell types (cancer cells, MDSCs, DCs, *C1QC* + TAMs, SPP*1* + TAMs, iCAFs, myCAFs, *CD8* + T cells, *CD4* + T cells). Left: untreated samples, right: treated samples. CAF cancer-associated fibroblast, DC dendritic cell, iCAF inflammatory CAF, LRI ligand-receptor interaction, MDSC myeloid-derived suppressor cell, myCAF myofibroblastic CAF, TAM tumor-associated macrophage. **B** Heatmap displaying the fold change between the number of inferred LRIs in naïve and treated samples for each pair of cell types. Red indicates more inferred LRIs in naïve samples and blue indicates more inferred LRIs in treated samples. **C** Dot plot of CellphoneDB output (see Methods) for recruitment-associated LRIs between iCAFs/myCAFs and SPP*1* + /*C1QC* + TAMs comparing untreated and treated samples. **D** Differential gene expression of checkpoint molecules in *CD8* + T cells between treated and untreated samples. **E** Dot plot of CellphoneDB output (see Methods) for checkpoint molecule LRIs between *CD8* + T cells, SPP*1* + or *C1QC* + TAMs, and cancer cells comparing untreated and treated samples.

We applied CellPhoneDB^40^ to infer potential ligand-receptor interactions (LRIs) between malignant epithelial cells, TAM and CAF subpopulations, *CD8* + and *CD4* + T cells, MDSCs, and DCs in naïve and treated samples (Fig. 7A; more detailed results in Supplimentary Data 5), and found a general decrease of cell-cell interactions in the chemotherapy-treated group in our dataset (Fig. 7B). We noted an unexpectedly high volume of putative interactions between CAFs and TAMs, which is highlighted in Supplimentary Fig. 7A. Before replicating this analysis in our confirmatory dataset, we removed one sample, which had an extreme number of SPP*1* + cells (Supplimentary Fig. 7B) and was producing highly unusual results. The general overview of LRIs in the confirmatory dataset matched that of our original dataset, with the decrease in LRIs between naïve and treated samples being even more prevalent across compartments (Supplimentary Fig. 7C, D).

As CAFs are known to recruit and polarize macrophages, we checked for recruitment-associated ligand-receptor interactions between CAFs and TAMs^41^. There was evidence for only two ligand-receptor pair interactions in this analysis: low levels of the CSF1-CSF1R interaction that did not change with treatment, and an interaction between CXCL12 (on iCAFs but not myCAFs) and CXCR4 (on both TAM subpopulations). CXCL12-CXCR4, the strongest potential ligand-receptor interaction pair in naive samples, significantly weakened with treatment in both our dataset and the confirmatory dataset (Fig. 7C, Supplimentary Fig. 7E–H). Notably, it appears that the change in the volume of the CXCL12-CXCR4 interaction is driven by a decrease in CXCR4 in TAMs with treatment (Supplimentary Fig. 7F–H). The CXCL12-CXCR4 interaction between perivascular CAFs and TAMs has recently been linked to metastasis^42^ and may be one way in which conventional chemotherapy affects crosstalk in the TME.

Since checkpoint inhibitory drugs have yet to show benefit in PDAC clinical trials (except in the 1% of PDAC cases with mutations in mismatch repair genes^43^), we further evaluated treatment-related checkpoint molecule expression in *CD8* + T cells. We found *TIGIT* to be the highest and most broadly expressed inhibitory checkpoint molecule, while *PDCD1* was expressed at low levels and in a minority of *CD8* + T cells (Fig. 7D). In addition, expression of *TIGIT*, *PDCD1*, and other checkpoint molecules was lower in *CD8* + T cells of chemotherapy-treated samples than naive samples (Fig. 7D). The findings in Fig. 7D are replicated very closely in the confirmatory set (Supplimentary Fig. 7I). To further validate our findings, we used multiplex IHC to stain for CD8 + T cells, TIGIT, and PD-1 in corresponding tissue slides of treatment-naïve and treated and pancreatic samples (Supplimentary Fig. 7J). Using representative samples, we found that TIGIT was expressed in most CD8 + T cells in naive samples and to a significantly lesser extent in CD8 + T cells in treated samples (Supplementary Figure 7K). In addition, most CD8 + T cells did not show expression of PD-1 in either naive or treated samples (Supplimentary Fig. 7L). Of note, we found cells in the TME that are TIGIT positive but CD8 negative, representing expression in other immune cell types.

We also assessed whether treatment alters checkpoint molecule interactions between CD8 + T cells, TAMs, and malignant cells. We evaluated all *CD8* + T cells due to the scarcity of exhausted T cells in our dataset. Moreover, CD8 + T cells can exhibit different states of exhaustion, and the larger CD8 + population is more representative of response regarding checkpoint molecule interactions with chemotherapy^44^. We observed more potential ligand-receptor interactions between TAMs and *CD8* + T cells than between cancer cells and *CD8* + T cells in both our samples (Fig. 7E; Supplimentary Fig. 7M, N) and the confirmatory dataset (Supplimentary Fig. 7O–P). Comparing naïve and treated samples, we noticed that all of the putative inhibitory ligand-receptor interactions were significant only in naive samples, while immune stimulatory interactions between TAMs and *CD8* + cells were significant in both treated and naive samples (Fig. 7E). The only significant PD-1 (PDCD1) interaction occurred in naive samples, between PD-1 on *C1QC* + TAMs and PD-L2 (PDCD1LG2) on *CD8* + T cells (Fig. 7E); this interaction was not present in the confirmatory dataset (Supplimentary Fig. 7O). We only detected three significant interactions between *CD8* + T cells and cancer cells, with TIGIT-PVR being the strongest. TIGIT-PVR was also the only interaction that was still significant in treated samples, though the effect was weaker than in naive samples (Fig. 7E). Of those three, only PVR-TIGIT was found in the confirmatory dataset (Supplimentary Fig. 7O). Of note, we did not detect any PD-1 related interactions between *CD8* + T cells and cancer cells. We next performed multiplex IHC to assess coexpression of TIGIT + on CD8 + T cells and PVR + on CK19 + cancer cells at the protein level. We detected both cell types expressing the ligand-receptor pair in close proximity to each other, supporting the potential for ligand-receptor interactions between those cell types (Supplimentary Fig. 7Q).

## Discussion

In this study we systematically characterized the composition of human PDAC at the single-cell transcriptomic level and assessed the effects of chemotherapy on the TME. Individual samples demonstrated significant heterogeneity in their Moffitt subtypes and TME composition. Analysis of the TME revealed discrete cell subpopulations with distinct features, further highlighting the complexity of the PDAC TME. We found, and confirmed in an external dataset, that chemotherapy profoundly alters the PDAC TME and might lead to further resistance to immunotherapy by reduced inhibitory checkpoint molecule expression and interactions involving CD8 + T cells.

Consistent with previous reports on subtype heterogeneity, our initial per-sample analysis of cancer cell composition revealed a heterogeneous mixture of cells with classical or basal transcriptional signatures in most samples and a variety of subclonal growth patterns^22,25,45,46^. A recent study demonstrating plasticity of pancreatic cancer cells and subtype switching dependent on culture conditions in vitro also identified a new intermediate subtype state^25^. Although we did not observe this specific intermediate state gene signature (the number of samples in each dataset may limit the generalizability of such a signature), we did discover cancer cells in numerous patient samples that expressed both basal and classical histological markers to varying degrees. Since both subtypes exhibit different biology and metabolic features^47,48^, using scRNA-seq to evaluate individual subtype composition prior to therapy might help understand how these elements dictate responses to different therapeutic approaches.

In recent years, investigation of CAFs has led to the identification of distinct CAF subpopulations in human and mouse PDAC^19^. While iCAFs and myCAFs were present in our dataset, neither unsupervised clustering nor signature analysis revealed a defined apCAF population. Our finding is also consistent with a recent publication on single nucleus sequencing in human PDAC^49^. Nevertheless, a selection of iCAFs and myCAFs did co-express CD74 and HLA genes, consistent with another recent report^22^. Plasticity between CAF subtypes, as hypothesized by multiple groups, might explain the differences in CAF subpopulation findings^19,29^.

Myeloid cell analysis led to the identification of SPP*1* + and *C1QC* + TAMs as the major TAM subpopulations in human PDAC. In our data, M1/M2 polarization did not fully match these recently described subpopulations, suggesting that M1/M2 classification does not capture the complexity of TAM subpopulations in the human TME^50–52^. Further characterization showed enrichment for EMT and a high angiogenesis score in SPP*1* + TAMs, while *C1QC* + TAMs were enriched for antigen presentation and phagocytosis. These findings are consistent with recent scRNA-seq studies in CRC^38,53^. A SPP*1* + ^high^/*C1QC* + ^low^ TAM signature has been associated with worse overall survival in the TCGA CRC dataset, and SPP*1* + TAMs have been linked to CRC liver metastases^38,54^. In our analysis of both TAM signatures in the TCGA PDAC dataset, TAM subtype combination did not correlate with worse overall survival. Since the TCGA PDAC dataset has only bulk transcriptomics and a limited variety of clinical stages, the role of SPP*1* + and *C1QC* + TAMs in PDAC will require further evaluation in a larger, non-bulk RNA clinical cohort.

For a more unambiguous analysis of chemotherapy effects on the TME we compared naive and treated samples from primary tumors only. Recent studies have correlated the classical subtype with better therapy response than the more aggressive basal subtype and proposed that chemotherapy might shift the Moffitt subtype from classical to basal^55,56^.However, when assessing for a basal and classical multi-gene signature we found heterogeneity between tumors in regard to Moffitt subtype composition but no shift toward a basal subtype in chemotherapy-treated samples, nor a significant difference in intratumor heterogeneity. Taken together, the heterogeneity within each tumor and differences in individual gene expression imply that PDAC subtype categorization should not only rely on single marker analysis. Since individual genes might not reveal biological changes in cancer cells with treatment, we also checked for gene expression programs with GSEA and noticed a Moffitt-independent shift in enrichment of angiogenesis and EMT genes in treated samples. EMT is a hallmark feature of tumor invasion, and different EMT programs have been linked to PDAC progression, dissemination, and resistance to chemotherapy^57–59^. While the complexity of EMT makes therapeutic approaches targeting it challenging, novel approaches would be highly impactful^60^. Tumor angiogenesis and microvessel density have been linked to metastasis in PDAC^61–63^; while anti-angiogenic drugs have thus far not yielded clinical results, the question of how chemotherapy affects the microvasculature in PDAC warrants further investigation^64^.

The complexity of PDAC is not only determined by the cancer cell heterogeneity but also the intricate composition of and communication between cells in the TME. In our analysis, we found a high volume of putative ligand receptor interactions between TAMs and CAFs. There is limited data on crosstalk between CAFs and TAMs in the TME, even though these interactions likely play an important role in PDAC biology and may reveal potential targets for therapy^41^. We found CXCL12-CXCR4, specific to iCAFs and TAMs respectively, to be the most significant potential interaction. Notably, it has been demonstrated that CXCL12 secretion by CAFs in PDAC promotes immune evasion by T cell exclusion^65^, and CXCL12-CXCR4 interaction between perivascular CAFs and TAMs promotes metastasis in a breast cancer model^42^. In our dataset, CXCL12-CXCR4 interaction between iCAFs and TAMs was significantly lower in treated samples, exemplifying how conventional chemotherapy may alter the PDAC TME in ways we could not previously observe.

Although effective in a number of other malignancies, checkpoint inhibition therapy has performed poorly in PDAC. While the underlying biology is still being uncovered, few studies have explored checkpoint expression in human PDAC samples at the single cell level. In our analysis, exhausted *CD8* + T cells constituted only 2.8% of all *CD8* + T cells. This corresponds with previous studies showing PDAC to be a relatively immune-cold tumor with low PD-1 expression and might be a reason why checkpoint inhibition has failed in clinical trials for PDAC^4,5,66^. The strongest interaction we observed between *CD8* + T cells and cancer cells was TIGIT-PVR, which significantly decreased with treatment. Recently, Steele et al. found elevated TIGIT expression levels in tumor-infiltrating CD8 + T cells compared to CD8 + T cells from normal pancreatic tissue, but no changes in PD1 between the two groups^21^. Functionally, Freed-Pastor et al. used a PDAC mouse model to show that the TIGIT-PVR axis is critical for maintaining immune evasion and that TIGIT blockage elicited an immune response to PDAC^67^. *TIGIT* was the most expressed inhibitory checkpoint molecule on *CD8* + T cells and its expression, as with other checkpoint molecules, decreased with treatment. Lately, another study using single-nucleus sequencing in PDAC also found *TIGIT* overexpressed in *CD8* + T cells in naive samples^49^. These findings and ours imply that TIGIT may be a stronger immunotherapy candidate than PD-1 in PDAC. The higher *TIGIT* expression in treatment-naive samples further suggests that blocking TIGIT may be more effective given as first-line monotherapy or administered concurrently with chemotherapy, and that immune blockade as second-line treatment following chemotherapy may lessen its antitumor effects. While a recent phase III trial of tiragolumab as first line therapy in small cell lung cancer was unsuccessful^68^, the Morpheus-Pancreatic Cancer trial (NCT03193190) is investigating the effect of TIGIT blockage in metastatic PDAC and will hopefully provide more insight into the clinical relevance of TIGIT inhibition in PDAC. Overall, our results provide an in-depth view into human PDAC composition, its TME, and the effects of chemotherapy, and may provide insight into more effective strategies to treat this recalcitrant disease.

## Methods

### Patient cohort and data collection

A total of 27 PDAC patients were recruited for study participation at the Perlmutter Cancer Center at New York University (NYU) Langone Health between May 1, 2020, and June 30, 2021. Informed written consent for the collection of blood, tissue, and clinical information was obtained from all patients using a study protocol approved by the NYU Langone Health Institutional Review Board. Standard clinicopathological variables including sex, age, tumor site, disease stage, type of procedure, tumor stage (TNM classification), and treatment type were collected for each patient as part of a prospective PDAC database. Clinical germline and somatic tumor analyses (Tempus xT 596 gene panel, Tempus Labs, Chicago, IL; Invitae 91 gene panel, Invitae Corporation, San Francisco, CA; FoundationOne CDx 324 gene panel, Foundation Medicine, Cambridge, MA) were performed. A supplement providing individual UMAP, H&E as well as clinical and mutational information is provided as Supplementary Data 2.

### Sample acquisition and processing

Of the 27 samples, 10 were obtained following surgical resection, 7 were obtained from endoscopic ultrasound (EUS)-guided biopsies, and 10 were obtained by interventional radiology (IR)-guided biopsies from metastatic liver lesions. For EUS- and IR-guided biopsies, 2-3 cores were used for scRNA-seq analysis. The pathologic diagnosis of PDAC was confirmed in all samples by a board-certified pancreatic pathologist. Within two hours of tissue acquisition, the specimen was placed in refrigerated RPMI 1640 medium (Gibco, Cat. No. 11875101) and transported to the Center for Biospecimen Research & Development at NYU, where the sample was provided for tissue processing.

### Tissue processing and single cell isolation

Tissue samples were washed in ice-cold phosphate-buffered saline (PBS; Corning, Cat. No. 21-040-CM) and grossly assessed for adequacy (solid tissue component, free of large hemorrhagic or necrotic areas). Next, the tissue was manually minced with a scalpel blade and filtered through a 40 µm mesh filter (Falcon; Cat. No. 352340) to remove any excess debris or blood. The tissue was then transferred to a sterile gentleMACS C tube (Miltenyi Biotec, Cat. No. 130096334), resuspended in 3–5 mL of Miltenyi Tumor Dissociation Kit buffer (Cat. No. 130-095-929), and mechanically minced to submillimeter particles. The suspension was then digested utilizing the gentleMACS Octo Dissociator (Miltenyi Biotec, Cat. No. 130-095-937) for 25–30 min using the “37C_h_TDK_3” program for tumor tissue. Dissociated cells were then quenched with ice-cold RPMI 1640 containing 10% fetal bovine serum (FBS; Gibco, Cat. No. 10082147) and filtered through a 40 µm mesh filter. Red blood cells were removed by treatment with 3–5 mL of ACK lysis buffer (Gibco, Cat. No. A1049201) and a subsequent washing step in RPMI 1640 with 10% FBS. Final cell counts were established with 0.4% trypan blue solution and a Countess II Automated Cell Counter (Applied Biosystems, Cat. No. A27977). The final concentration was titrated to 300–500 viable cells/µL for further analysis.

### Single-cell library preparation

Single-cell suspensions were processed for 10x Genomics by the Genome Technology Center at the NYU School of Medicine per the manufacturer’s guidelines. The libraries were prepared using the following reagents: Chromium Single Cell 3′ Reagent Kits (v3)—the Single Cell 3′ Library & Gel Bead Kit v3 (PN-1000075), the Chromium Next GEM Chip G Single Cell Kit (PN-100120), and the i7 Multiplex Kit (PN-120262) (10x Genomics) —by following the Single-Cell 3′ Reagent Kits (v2) User Guide (manual part no. CG00052 Rev A) and the Single-Cell 3′ Reagent Kits (v3) User Guide (manual part no. CG000183 Rev C). DNA libraries were run using the NovaSeq 6000 system (Illumina, San Diego, CA) to achieve roughly 500 million paired-end reads per sample.

### Single-cell RNA sequencing and data processing

Sequencing results were de-multiplexed and converted to FASTQ format using Illumina bcl2fastq software. The 10x Genomics Cell Ranger 5.0.1 software suite^69^ was used to perform sample de-multiplexing, barcode processing, and single-cell 3’ gene counting. The cDNA insert was aligned to the hg38/GRCh38 reference genome. Only confidently mapped, non-PCR duplicates with valid barcodes and unique molecular identifiers were used to generate the gene-barcode matrix. Further analysis including the identification of highly variable genes, dimensionality reduction, standard unsupervised clustering algorithms, and the discovery of differentially expressed genes was performed using Seurat^70^ and scooter^71^.

Raw data were initially filtered to remove ambient RNA using SoupX version 1.5.2^72^; and cells were subsequently filtered to include only high-quality cells (as defined by >500 detectable genes, >1500 unique molecular identifiers, and <15% of transcripts coming from mitochondrial genes). Cells with >1% of transcripts representing erythroid genes (HBA1, HBA2, HBB, HBM, and ALAS2) were also excluded from the analysis. The data were normalized to the total expression, multiplied by a scaling factor of 10,000, and log-transformed. Likely doublets/multiplets were identified and removed using scDblFinder 1.6.0^73^.

To account for biological and technical batch differences between individual patients and scRNA-seq libraries, the Seurat anchor-based integration method for merging datasets that identify pairwise correspondences between cell pairs across datasets^74^ was utilized to transform them into a shared space. The 2000 most variable genes based on standardized variance were selected for canonical correlation analysis as an initial dimensional reduction. The integration anchors were then identified based on the first 30 dimensions and used to generate a new dimensional reduction for further analysis.

To visualize the data, the dimensionality of the scaled integrated data matrix was further reduced to project the cells in two-dimensional space using principal component analysis followed by UMAP^75^. The 30 nearest neighbors were used to define the local neighborhood size with a minimum distance of 0.3. The resulting principal components were also used as a basis for partitioning the dataset into clusters using a smart local moving community detection algorithm^76^. A range of resolutions (0.1–10) was utilized to establish a sufficient number of clusters to separate known populations based on the expression of established markers.

### Cell identification and clustering

Single-cell clusters were identified based on common marker genes for various cell types, the most prominent of which are listed here. Full dataset (Fig. 1C): T/NK (*CD3E*), epithelial (*KRT19*), endothelial (*VWF, PECAM1*), myeloid (*CD68*), proliferating epithelial (*KRT19, MKI67*), proliferating lymphoid (*CD3E, MKI67*), B/plasma (*CD79A*), proliferating myeloid (*CD68, MKI67*), mesenchyme (*DCN*), mast (*KIT*). Mesenchyme (Fig. 3A): myCAF (*ACTA2, MMP11, COL10A1*^−^), iCAF (*C3, C7, CFD, PTGDS*), pericyte (*ACTA2*^*+*^*, RGS5*^*+*^*, CSPG4, NOTCH3*), proliferating (*MKI67*), peri-islet Schwann cells (*SOX10, S100B, PLP1*), epithelial-like (*KRT19, EPCAM*), chondrocyte-like (SPP*1, IBSP, MMP13*). T/NK (Fig. 4A): *CCR7* + *CD4* + T cells (*CCR7*), *IL7R* + *CD4* + T cells (*IL7R*), *FOXP3* + *CD4* + T cells (*FOXP3*), *CXCL13* + *CD4* + T cells (*CXCL13*), *GZMH* + *CD8* + T cells (*GZMH*), *GZMK* + *CD8* + T cells (*GZMK*), *ITGA1* + *CD8* + T cells (*ITGA1*), *ISG15* + T cells (*ISG15*), *GNLY* + NK cells (*GNLY, NCAM1*), *XCL1* + NK cells (*XCL1, NCAM1*), *KIT* + mast (*KIT*), *CD79* + B/Plasma (*CD79A*), *MKI67* + proliferating (*MKI67*). *CD8* + T cells (Fig. 4C): *GZMK* + *CD8* + T cells (GZMK), *GZMH* + *CD8* + T cell (*GZMH*), *ITGA1* + *CD8* + T cells (*ITGA1*), *SELL* + *CD8* + T cells (*SELL*), *ISG15* + *CD8* + T cells (*ISG15*), *CTLA4* + *CD8* + T cells (*CTLA4*), *HSPA6* + *CD8* + T cells (*HSPA6*), epithelial-like (*KRT19*), erythrocytes (*HBB*). Myeloid (Fig. 5): SPP*1* + macrophage (SPP*1, MARCO*), *C1QC* + macrophage (*C1QA, C1QB, C1QC*), MDSC (*S100A8, S100A9, S100A12*), monocyte (*FCGR3A, CDKN1C*), cDC1 (*CLEC9A, XCR1*), cDC2 (*CD1C, FCER1A*), cDC3 (*CCL19, CCL22, CCR7*), pDC (*LILRA4, PLD4*), proliferating (*MKI67*), mast (*KIT*), T (*CD3E*), epithelial-like (*KRT18, KRT19*).

### Identification of malignant and pancreatic epithelial cells

To detect large-scale chromosomal CNVs, InferCNV version 1.8.1^77^ was run at a sample level with the parameters recommended for 10x Genomics data. For each sample, all epithelial cells were tested for copy number aberrations. Non-epithelial (CAF, endothelial, immune) cells were used as the reference normal set. For samples with over 1,000 non-epithelial cells, they were randomly subsampled to 1000. Additionally, 10% of non-epithelial cells were used in the observation set. We used a 101-gene window in subclustering mode with HMM enabled and a six-group clustering of observations.

### Ligand-receptor interaction

CellPhoneDB v.2.1.7^40^ was used to estimate ligand-receptor interaction. We used the statistical method with 10 iterations and no subsampling; other parameters were left as default. CPDB calculates statistical significance using permutation testing; see original paper for details.

### Gene signature scores

Gene signature scores were created using Seurat’s AddModuleScore function. Lists of genes used for each score can be found in Supplementary Data 3.

### Differential gene expression

Differential gene expression was analyzed using the Wilcoxon rank-sum test with Bonferroni correction that is included in Seurat’s FindMarkers function.

### Gene set enrichment analysis

Gene set enrichment analysis was performed using the GSEAPreranked function of GSEA version 4.2.0^78^. Genes were pre-ranked for each comparison using the output of our differential gene expression; first by taking the -log_10_(adjusted p-value) and then multiplying it by the sign of the fold change (for example, a gene with a fold change of −2.3 and an adjusted p-value of 1e-160 would have a rank metric of −160). GSEAPreranked parameters were set to the defaults. The gene chip annotation file used was Human_Gene_Symbol_with_Remapping_MSigDB.v7.4.chip, and genes were collapsed and remapped to gene symbols. Gene sets used included Hallmarks (h.all.v7.5.1), KEGG (c2.cp.kegg.v7.5.1), Reactome (c2.cp.reactome.v7.5.1), Canonical Pathways (c2.cp.v7.5.1), and GO (c5.go.v7.5.1).

### Survival analysis of TCGA data

The TCGA PDAC (PAAD) clinical and gene expression data^9^ were downloaded, using TCGABiolinks version 3.15^79^, to examine the prognostic utility of specific cell subpopulation signatures. Gene expression was z-score transformed. For each signature, two distinct groups (low and high expression) were formed, corresponding to mean expression values below the 45th and above the 55th quantile values, respectively. The survival analysis was performed using the R package survminer^80^. Cox proportional hazards models from the R package survival^81^ were used to adjust for tumor stage, age, and sex.

### Histology and multiplex IHC

For histopathological assessment, tumor sections were cut and slides prepared by routine processes, followed by staining with hematoxylin & eosin. For multiplex immunofluorescence and imaging, five-micron formalin fixed paraffin-embedded sections were stained with Akoya Biosciences® Opal™ multiplex automation kit reagents (Leica Cat #ARD1001EA) on a Leica BondRX® autostainer, according to the manufacturers’ instructions. In brief, slides underwent sequential epitope retrieval with Leica Biosystems Epitope Retrieval 2 solution (ER2, EDTA based, pH 9, Cat. AR9640), primary and secondary antibody incubation, and tyramide signal amplification with Opal® fluorophores as shown in Supplementary Table 3. Primary and secondary antibodies/polymers were removed during sequential epitope retrieval steps while the relevant fluorophores remained covalently attached to the antigen. Slides were counterstained with spectral DAPI (Akoya Biosciences, FP1490) and mounted with ProLong Gold Antifade (ThermoFisher Scientific, P36935). Semi-automated image acquisition was performed on a Vectra® Polaris multispectral imaging system. After whole slide scanning at 20x in the motif mode, regions of interest were selected for spectral unmixing and image processing using InForm® version 2.4.10 software from Akoya Biosciences.

### IHC Quantification

Semi-automated image acquisition was performed on a Vectra® Polaris multispectral imaging system. Eleven cases were used for this quantification. After whole slide scanning at 20X the tissue was manually outlined to select fields for spectral unmixing and image analysis using InForm® version 2.6 software from Akoya Biosciences. Selection of areas was performed by a trained pathologist on H&E-stained sequential sections and copied onto the fluorescent image on Akoya Biosciences Phenochart software. A training algorithm was first used to segment images into tissue categories: “CK19 + ” or “CK19-” (to exclude areas of the image with no tissue). Tissue segmentations were reviewed for different samples during training iterations. Following tissue classification cells were segmented based on nuclear signal (DAPI). Cells were phenotyped after segmentation using inForm’s trainable algorithm based on the R glmnet package^82^.

Four algorithms were created to classify cells as CD8 + or ‘other’, CK19 + or ‘other’, TIGIT + or ‘other’, and PD1 + or ‘other’. Phenotypes were reviewed for different samples during training iterations. Data was exported as text containing sample names, field of acquisition coordinates, individual cell information including coordinates and identified phenotype. Each image was analyzed with all four algorithms so that every cell was classified four times. Concatenation and aggregation of all phenotyping information was performed in R using the phenoptrReports package^83^.

### Reporting summary

Further information on research design is available in the Nature Portfolio Reporting Summary linked to this article.

## Supplementary information

Supplementary Information

Description of Additional Supplementary Files

Supplementary Data 1

Supplementary Data 2

Supplementary Data 3

Supplementary Data 4

Supplementary Data 5

Reporting Summary

## Data Availability

The raw scRNA-seq data generated for this project is available on GEO with accession number GSE205013. TCGA data^9^ is publicly available on the GDC Data Portal [https://portal.gdc.cancer.gov/]. The confirmatory dataset from Cui Zhou et al.^39^ is publicly available at the Human Tumor Atlas Network (HTAN) under the HTAN WUSTL Atlas [https://humantumoratlas.org/explore?selectedFilters=%5B%7B%22group%22%3A%22AtlasName%22%2C%22value%22%3A%22HTAN+WUSTL%22%7D%5D&tab=file] and on dbGaP with accession number phs002371.v2.p1. The remaining data are available within the article and its supplementary files.
