# Author Correction: Single-cell RNA sequencing reveals the effects of chemotherapy on human pancreatic adenocarcinoma and its tumor microenvironment

**DOI:** 10.1038/s41467-023-39680-2

**Published:** 2023-07-03

**Authors:** Gregor Werba, Daniel Weissinger, Emily A. Kawaler, Ende Zhao, Despoina Kalfakakou, Surajit Dhara, Lidong Wang, Heather B. Lim, Grace Oh, Xiaohong Jing, Nina Beri, Lauren Khanna, Tamas Gonda, Paul Oberstein, Cristina Hajdu, Cynthia Loomis, Adriana Heguy, Mara H. Sherman, Amanda W. Lund, Theodore H. Welling, Igor Dolgalev, Aristotelis Tsirigos, Diane M. Simeone

**Affiliations:** 1 Department of Surgery, NYU Langone Health, New York, NY 10016 USA; 2 Perlmutter Cancer Center, NYU Langone Health, New York, NY 10016 USA; 3 Department of Medicine, NYU Langone Health, New York, NY 10016 USA; 4 Department of Pathology, NYU Langone Health, New York, NY 10016 USA; 5 Department of Cell, Developmental and Cancer Biology, Oregon Health Sciences University, Portland, OR 97239 USA; 6 Department of Dermatology, NYU Langone Health, New York, NY 10016 USA

**Keywords:** Pancreatic cancer, Cancer microenvironment, Pancreatic cancer, Bioinformatics, Cancer microenvironment

Correction to: *Nature Communications* 10.1038/s41467-023-36296-4, published online 13 February 2023

In this article the grant number T32CA193111 relating to the National Institutes of Health for Dr Grace Oh was omitted. In addition, the following disclaimer statement was omitted and should have read ‘Its contents are solely the responsibility of the authors and do not necessarily represent the official views of the NIH. The original article has been corrected.

