# Supplementary material for: Single-cell RNA sequencing reveals the effects of chemotherapy on human pancreatic adenocarcinoma and its tumor microenvironment
Source: Nat Commun. 2023 Feb 13;14:797. doi: 10.1038/s41467-023-36296-4 (PMC9925748; doi:10.1038/s41467-023-36296-4)
Supplement: Supplementary file 1 — Supplementary Information [file 41467_2023_36296_MOESM1_ESM.pdf]

A

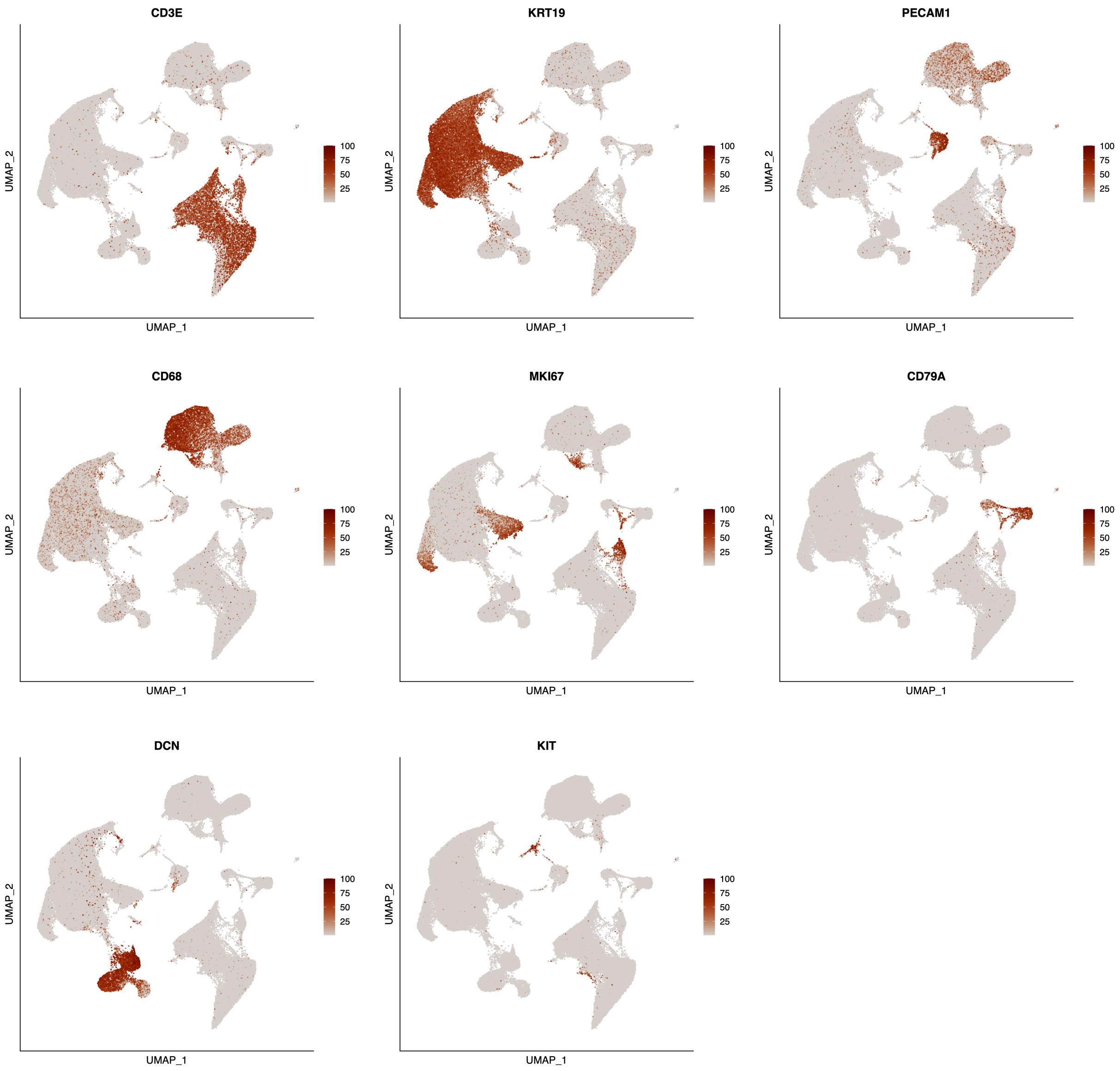

CD3E
KRT19
PECAM1
CD68
MKI67
CD79A
DCN
KIT
UMAP_1
UMAP_2
100
75
50
25

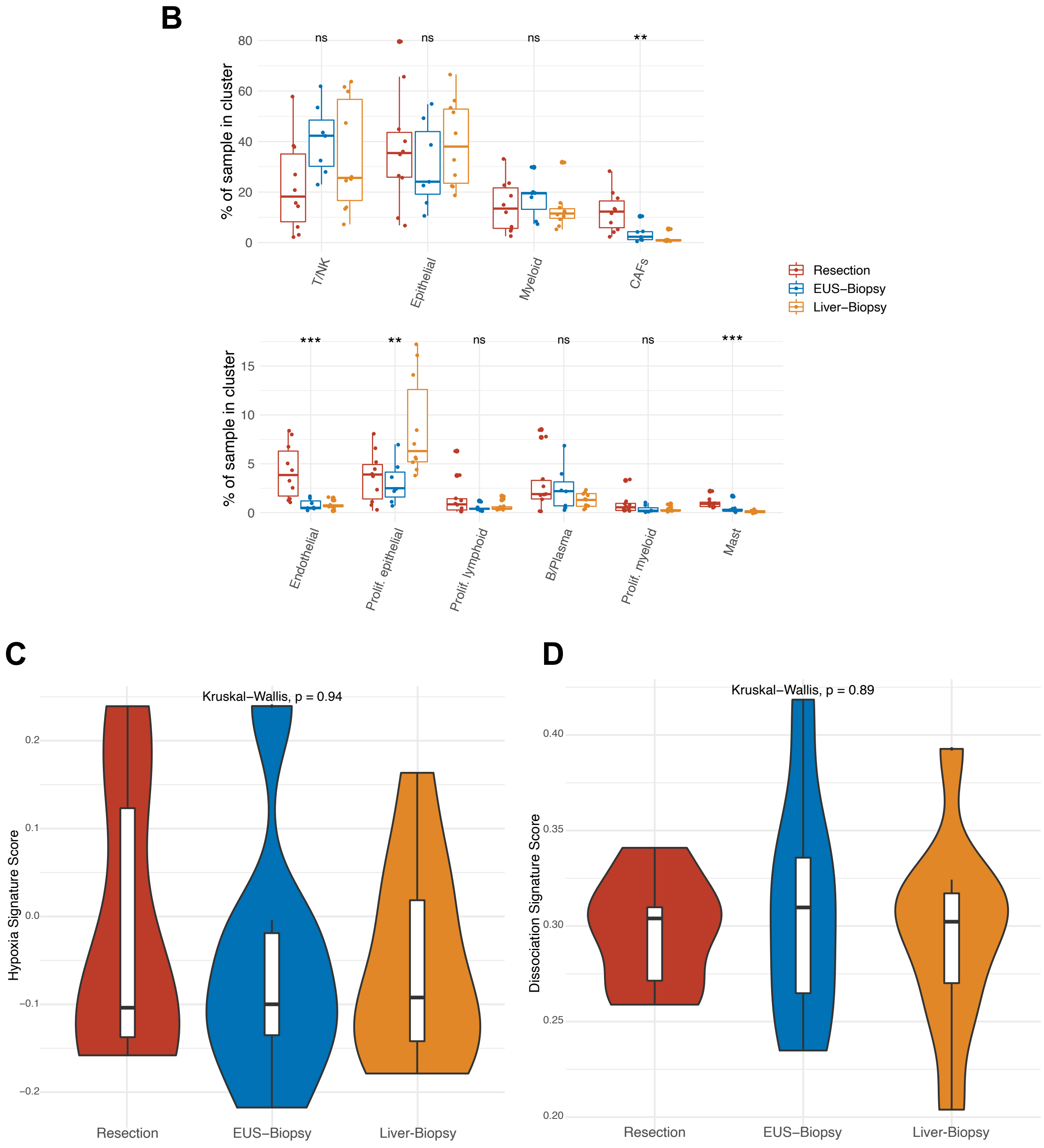
B
% of sample in cluster
80
60
40
20
0
ns
ns
ns
**
T/NK
Epithelial
Myeloid
CAFs
Resection
EUS–Biopsy
Liver–Biopsy
15
10
5
0
***
**
ns
ns
ns
***
Endothelial
Prolif. epithelial
Prolif. lymphoid
B/Plasma
Prolif. myeloid
Mast
C
Kruskal–Wallis, p = 0.94
Hypoxia Signature Score
0.2
0.1
0.0
−0.1
−0.2
Resection
EUS–Biopsy
Liver–Biopsy
D
Kruskal–Wallis, p = 0.89
Dissociation Signature Score
0.40
0.35
0.30
0.25
0.20
Resection
EUS–Biopsy
Liver–Biopsy

Supplementary Figure 1.

A. UMAP embeddings of canonical marker gene expression, utilized in the identification of clusters. UMAP, uniform manifold approximation and projection.

B. Proportional distribution of cell types per procedure (Resection [n=10], EUS [n=7], IR [n=10]; all box plots centered around the median with hinges at 1st and 3rd quartiles and whiskers from hinge to max value or 1.5*IQR, whichever is smallest; Kruskal-Wallis test ** = $p<0.01$, *** = $p<0.001$).

C. Quantification of hypoxia signature per procedure (Resection [n=10], EUS [n=7], IR [n=10]; Kruskal-Wallis test p=0.94).

D. Quantification of dissociation signature per procedure (Resection [n=10], EUS [n=7], IR [n=10] ; Kruskal-Wallis test p=0.89).

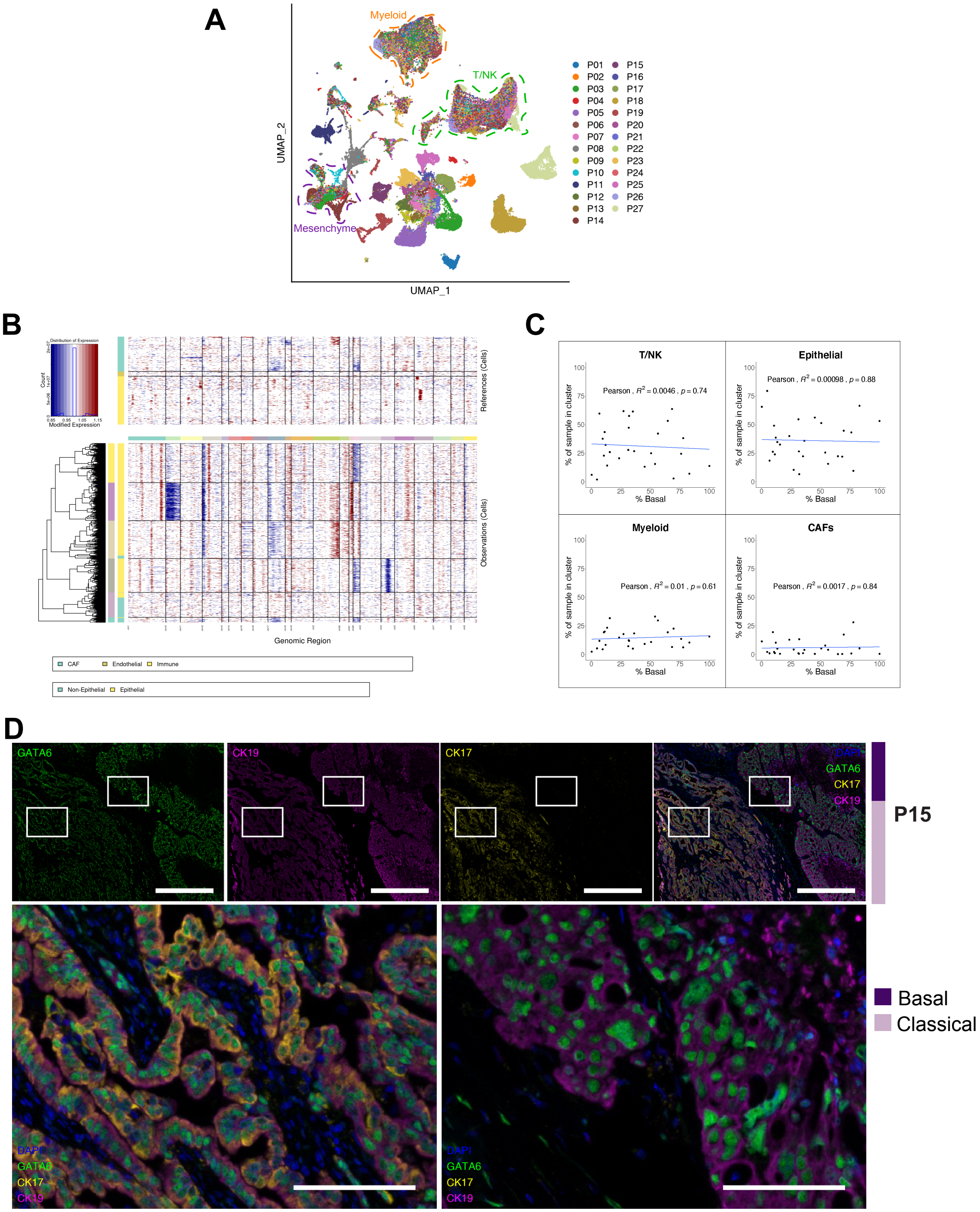
A
Myeloid
T/NK
Mesenchyme
UMAP_1
UMAP_2
P01 P02 P03 P04 P05 P06 P07 P08 P09 P10 P11 P12 P13 P14
P15 P16 P17 P18 P19 P20 P21 P22 P23 P24 P25 P26 P27
B
Distribution of Expression
Count
Modified Expression
References (Cells)
Observations (Cells)
Genomic Region
CAF
Endothelial
Immune
Non-Epithelial
Epithelial
C
T/NK
Pearson , $R^2 = 0.0046$, $p = 0.74$
Epithelial
Pearson , $R^2 = 0.00098$, $p = 0.88$
Myeloid
Pearson , $R^2 = 0.01$, $p = 0.61$
CAFs
Pearson , $R^2 = 0.0017$, $p = 0.84$
% of sample in cluster
% Basal
D
GATA6
CK19
CK17
DAPI
P15
Basal
Classical

E

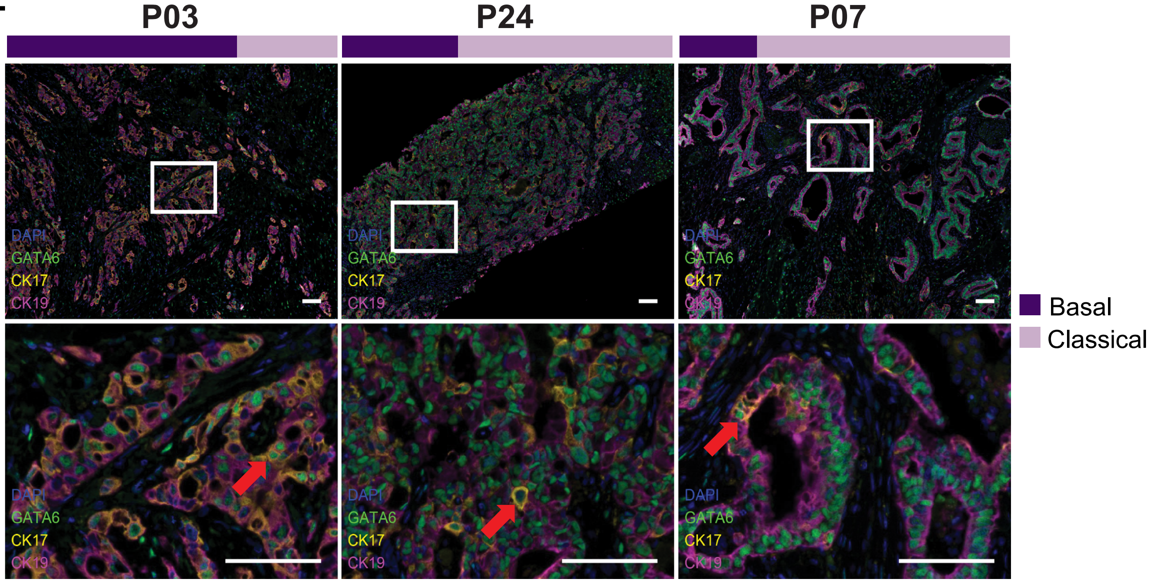
P03
P24
P07
DAPI
GATA6
CK17
CK19
Basal
Classical

F

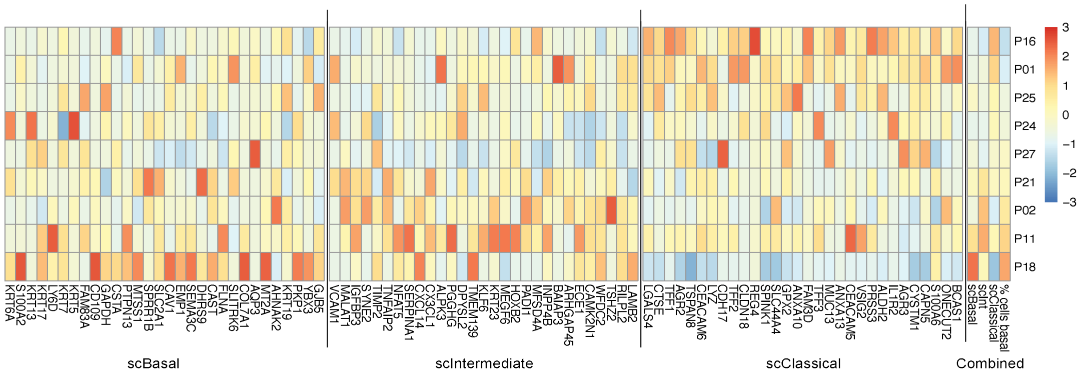
P16
P01
P25
P24
P27
P21
P02
P11
P18
3
2
1
0
−1
−2
−3
scBasal
scIntermediate
scClassical
Combined

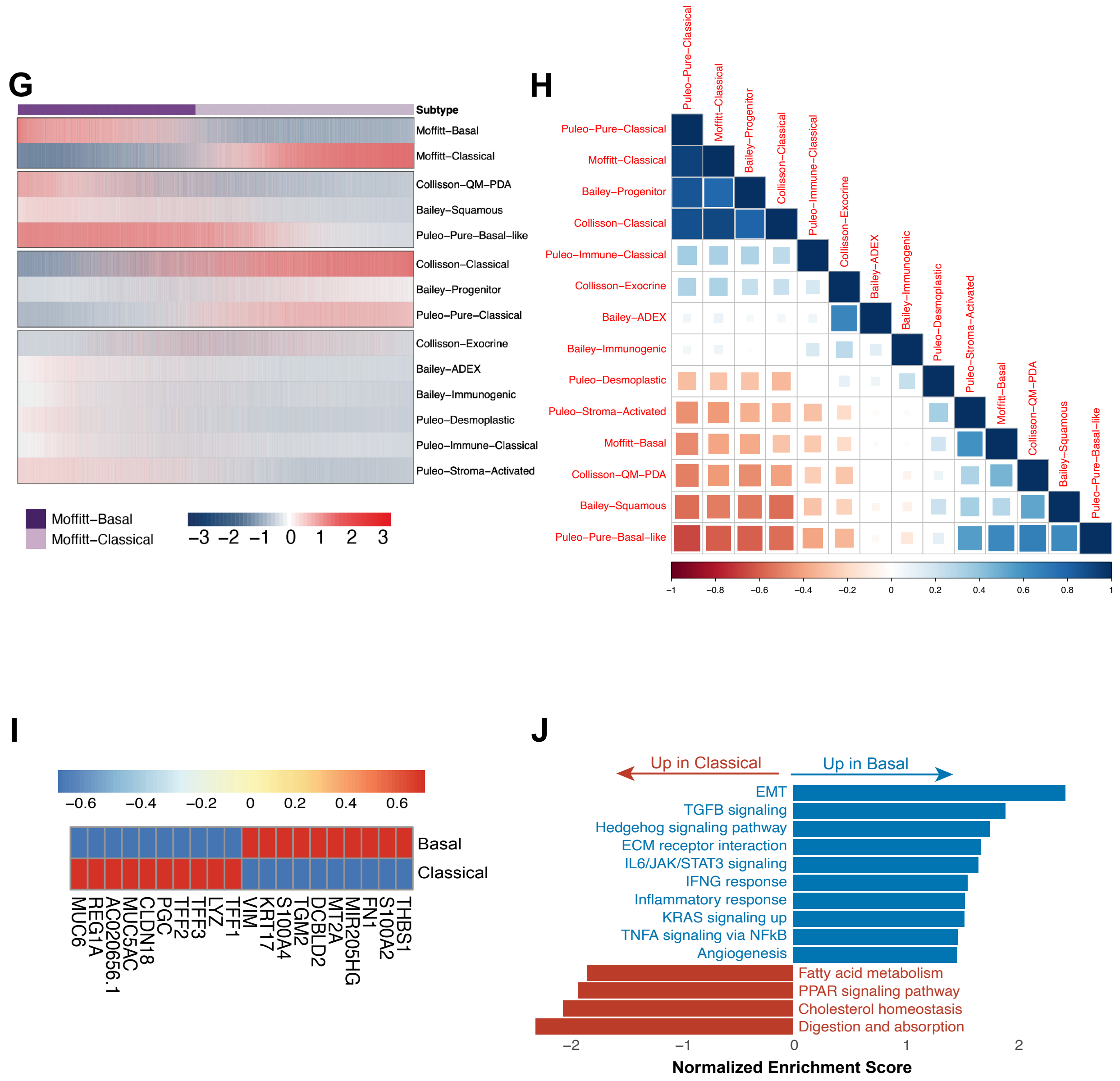

Supplementary Figure 2.

A. Non-batch corrected UMAP embedding reveals distinct transcriptomic landscapes in patient-specific tumor compartments, while cells of other lineages evince significant overlap. Clusters with non-epithelial cells are labeled with the majority cell type. UMAP, uniform manifold approximation and projection.

B. inferCNV output for a patient sample (P19) with more than one subclone indicated in the CNV analysis.

C. Correlation (two-sided Pearson correlation) between proportion of malignant epithelial cells in a sample that are labeled as basal and the percentage of the sample that is in each of four major clusters.

D. Representative images of multiplex immunofluorescence from a case with two distinct growth patterns (P15). Basal to classical ratio by scRNA-seq transcriptional analysis is shown on the right as colored bars (dark = basal, light = classical). Channels (always including DAPI (blue)): GATA6 (green), CK19 (cytokeratin 19) (violet), CK17 (cytokeratin 17) (yellow), and merged. Scale bar = 500µm. Corresponding high power merged images of two distinct regions are indicated in the low power image by white boxes, highlighting the different growth pattern and marker expression. Scale bar = 100µm.

E. Multiplex immunofluorescence images corresponding to main Fig. 2E (n=27). For each case, the merged low power image is shown (scale bar = 100µm), along with the corresponding high power image of the region indicated in a white box in the low power image (scale bar = 100µm). Red arrows indicate cells that express both GATA6 and CK17.

F. Reproduction of the scBasal, scIntermediate, and scClassical subtypes from Raghavan *et al.*[1] in our untreated liver samples (n=9). Samples are sorted from low to high by the percentage of cells determined to be basal using the Moffitt signature.

G. Heatmap showing subtype analyses per cell in correlation to subtype distribution in our dataset. Analyzed classifications were Moffitt (basal, classical)[2], Collisson (quasi-mesenchymal, classical, exocrine)[3], Bailey (squamous, progenitor, ADEX, immunogenic)[4], and Puleo (pure basal like, pure classical, desmoplastic, immune classical, stroma activated)[5].

H. Correlations between each subtype classification.

I. The top ten differentially expressed genes between the basal and classical malignant epithelial cells.

J. Selected gene set enrichment analysis results from a comparison between the classical and basal cancer cell subtypes.

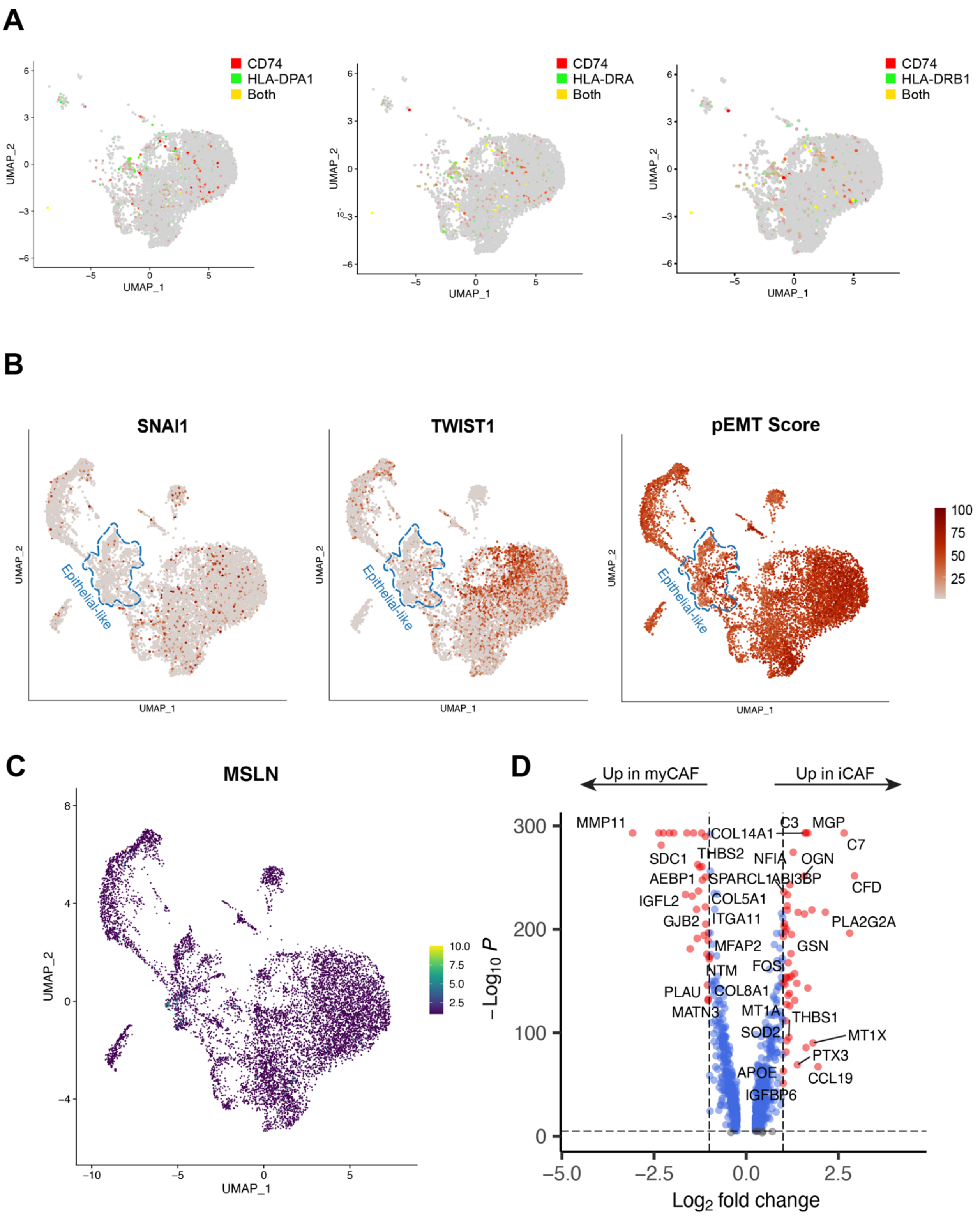
A
CD74
HLA-DPA1
Both
CD74
HLA-DRA
Both
CD74
HLA-DRB1
Both
UMAP_1
UMAP_2
B
SNAI1
TWIST1
pEMT Score
Epithelial-like
100
75
50
25
C
MSLN
10.0
7.5
5.0
2.5
D
Up in myCAF
Up in iCAF
MMP11
COL14A1
C3
MGP
C7
SDC1
THBS2
NFIA
OGN
AEBP1
SPARCL1
ABI3BP
CFD
IGFL2
COL5A1
GJB2
ITGA11
PLA2G2A
MFAP2
GSN
FOS
NTM
PLAU
COL8A1
MATN3
MT1A
THBS1
SOD2
MT1X
PTX3
APOE
CCL19
IGFBP6
$-\log_{10} P$
$\log_2$ fold change

Supplementary Figure 3.

A. UMAP of *CD74* and *HLA-DPA1*, *HLA-DRA*, and *HLA-DRB1* co-expression in CAFs. UMAP, uniform manifold approximation and projection.

B. Canonical EMT markers (*SNAI1* and *TWIST1*) and pEMT score[6] in the mesenchymal compartment suggest that the epithelial-like cluster does not consist of cells undergoing EMT. EMT, epithelial-mesenchymal transition.

C. UMAP of mesothelin gene expression in overall mesenchymal cells.

D. Differentially expressed genes between our iCAFs and myCAFs (n=16; Wilcoxon rank-sum test with Bonferroni correction). CAF, cancer-associated fibroblast; iCAF, inflammatory CAF; myCAF, myofibroblastic CAF.

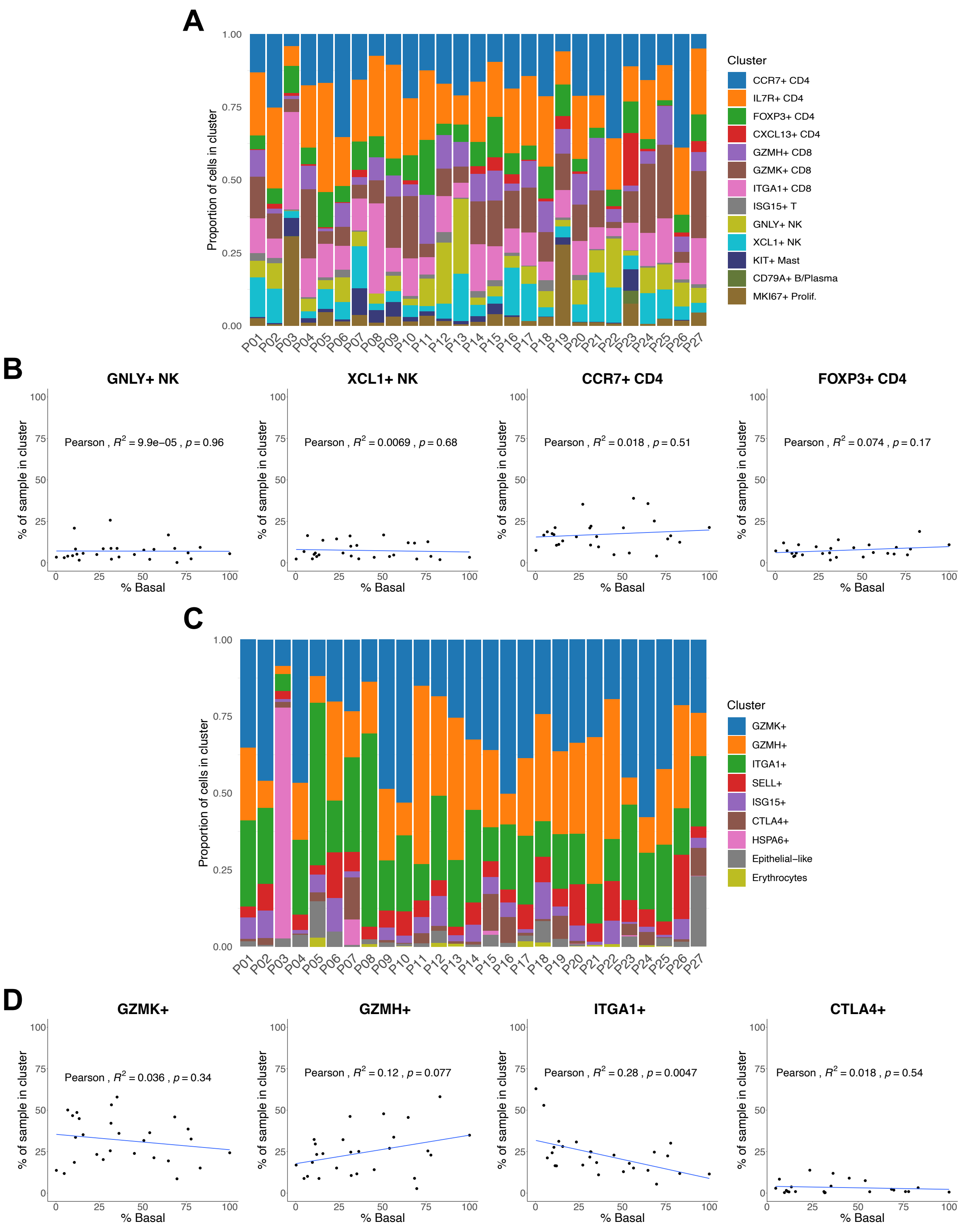
A
Proportion of cells in cluster
1.00
0.75
0.50
0.25
0.00
P01 P02 P03 P04 P05 P06 P07 P08 P09 P10 P11 P12 P13 P14 P15 P16 P17 P18 P19 P20 P21 P22 P23 P24 P25 P26 P27
Cluster
CCR7+ CD4
IL7R+ CD4
FOXP3+ CD4
CXCL13+ CD4
GZMH+ CD8
GZMK+ CD8
ITGA1+ CD8
ISG15+ T
GNLY+ NK
XCL1+ NK
KIT+ Mast
CD79A+ B/Plasma
MKI67+ Prolif.
B
GNLY+ NK
Pearson , R^2 = 9.9e−05 , p = 0.96
XCL1+ NK
Pearson , R^2 = 0.0069 , p = 0.68
CCR7+ CD4
Pearson , R^2 = 0.018 , p = 0.51
FOXP3+ CD4
Pearson , R^2 = 0.074 , p = 0.17
% of sample in cluster
% Basal
0 25 50 75 100
C
Proportion of cells in cluster
1.00
0.75
0.50
0.25
0.00
P01 P02 P03 P04 P05 P06 P07 P08 P09 P10 P11 P12 P13 P14 P15 P16 P17 P18 P19 P20 P21 P22 P23 P24 P25 P26 P27
Cluster
GZMK+
GZMH+
ITGA1+
SELL+
ISG15+
CTLA4+
HSPA6+
Epithelial−like
Erythrocytes
D
GZMK+
Pearson , R^2 = 0.036 , p = 0.34
GZMH+
Pearson , R^2 = 0.12 , p = 0.077
ITGA1+
Pearson , R^2 = 0.28 , p = 0.0047
CTLA4+
Pearson , R^2 = 0.018 , p = 0.54
% of sample in cluster
% Basal
0 25 50 75 100

Supplementary Figure 4.

A. Proportional distribution of each T/NK cell cluster by individual sample. NK, natural killer.

B. Correlation (two-sided Pearson correlation) between proportion of malignant epithelial cells in a sample that are labeled as basal and the percentage of the sample that is in each of four exemplary T/NK clusters (n=27).

C. Proportional distribution of each *CD8*+ T cell cluster by individual sample.

D. Correlation (two-sided Pearson correlation) between proportion of malignant epithelial cells in a sample that are labeled as basal and the percentage of the sample that is in each of four exemplary *CD8*+ T cell clusters (n=27).

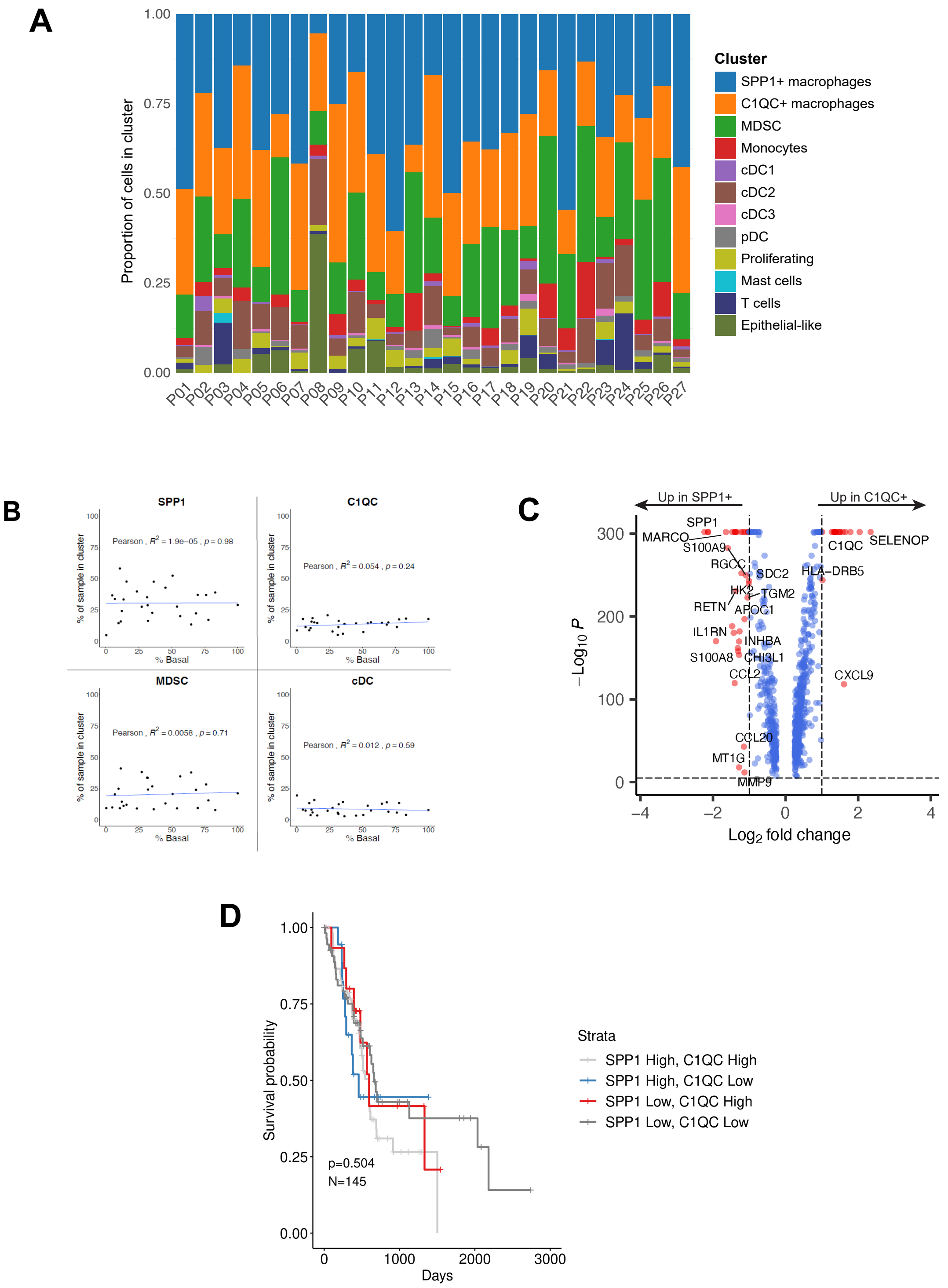
A
Proportion of cells in cluster
1.00
0.75
0.50
0.25
0.00
P01 P02 P03 P04 P05 P06 P07 P08 P09 P10 P11 P12 P13 P14 P15 P16 P17 P18 P19 P20 P21 P22 P23 P24 P25 P26 P27
Cluster
SPP1+ macrophages
C1QC+ macrophages
MDSC
Monocytes
cDC1
cDC2
cDC3
pDC
Proliferating
Mast cells
T cells
Epithelial-like
B
SPP1
Pearson , R^2 = 1.9e−05 , p = 0.98
C1QC
Pearson , R^2 = 0.054 , p = 0.24
MDSC
Pearson , R^2 = 0.0058 , p = 0.71
cDC
Pearson , R^2 = 0.012 , p = 0.59
% of sample in cluster
% Basal
C
Up in SPP1+
Up in C1QC+
SPP1
MARCO
S100A9
RGCC
SDC2
HK2
TGM2
RETN
APOC1
IL1RN
INHBA
S100A8
CHI3L1
CCL2
CCL20
MT1G
MMP9
C1QC
SELENOP
HLA−DRB5
CXCL9
$-\text{Log}_{10}$ P
300
200
100
0
−4 −2 0 2 4
Log_2 fold change
D
Survival probability
1.00
0.75
0.50
0.25
0.00
p=0.504
N=145
0 1000 2000 3000
Days
Strata
SPP1 High, C1QC High
SPP1 High, C1QC Low
SPP1 Low, C1QC High
SPP1 Low, C1QC Low

Supplementary Figure 5.

A. Proportional distribution of each myeloid cluster in each individual patient. DC, dendritic cell; MDSC, myeloid-derived suppressor cell.

B. Correlation (two-sided Pearson correlation, n=27) between the proportion of malignant epithelial cells in a sample that are labeled as basal and the percentage of the sample that is in each of the four exemplary myeloid populations.

C. Differentially expressed genes between *SPP1*+ and *C1QC*+ TAMs (n=27, Wilcoxon rank-sum test with Bonferroni correction).

D. PDAC TCGA data[7] on overall survival of patients with different combinations of *C1QC*+ and *SPP1*+ TAM signatures (n=53 SPP1 High/C1QC High, 18 SPP1 High/C1QC Low, 16 SPP1 Low/C1QC High, 57 SPP1 Low/C1QC Low; Cox proportional hazards model).

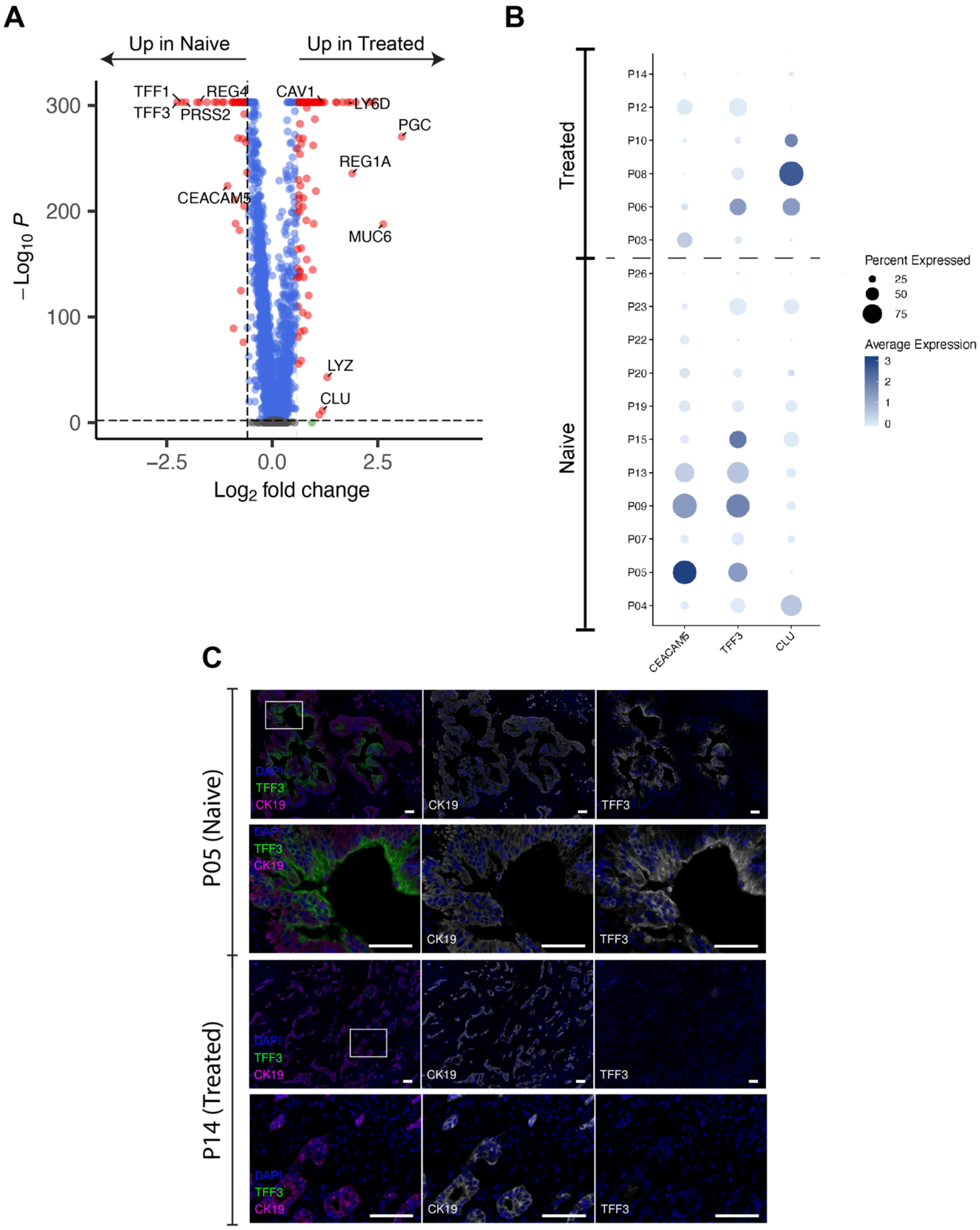
A
Up in Naive
Up in Treated
TFF1
REG4
TFF3
PRSS2
CAV1
LY6D
PGC
REG1A
CEACAM5
MUC6
LYZ
CLU
−Log10 P
Log2 fold change
B
Treated
Naive
P14
P12
P10
P08
P06
P03
P26
P23
P22
P20
P19
P15
P13
P09
P07
P05
P04
CEACAM5
TFF3
CLU
Percent Expressed
Average Expression
C
P05 (Naive)
P14 (Treated)
DAPI
TFF3
CK19

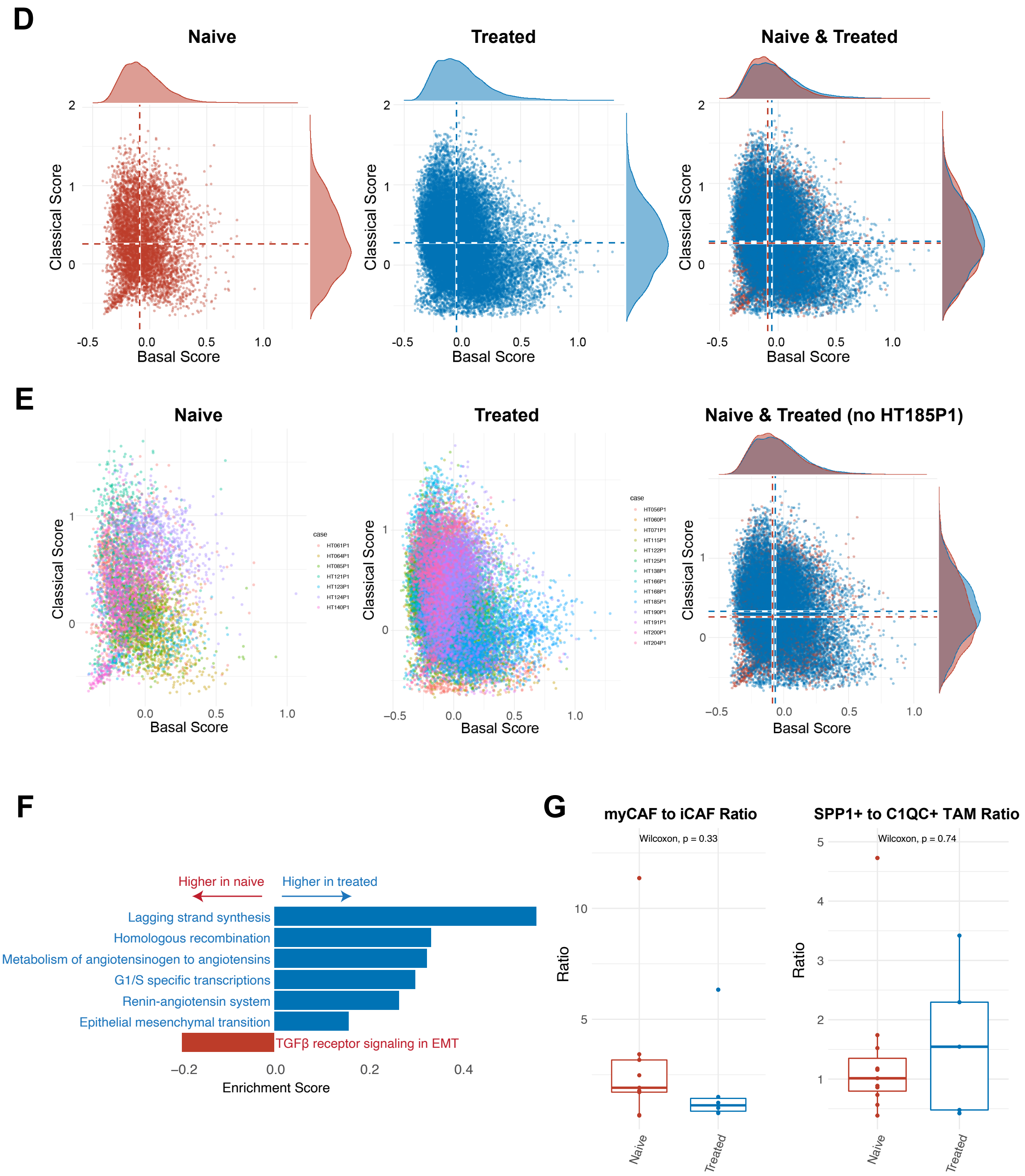
D
Naive
Treated
Naive & Treated
Classical Score
Basal Score
E
Naive
Treated
Naive & Treated (no HT185P1)
case
Classical Score
Basal Score
F
Higher in naive
Higher in treated
Lagging strand synthesis
Homologous recombination
Metabolism of angiotensinogen to angiotensins
G1/S specific transcriptions
Renin-angiotensin system
Epithelial mesenchymal transition
TGFβ receptor signaling in EMT
Enrichment Score
G
myCAF to iCAF Ratio
Wilcoxon, p = 0.33
SPP1+ to C1QC+ TAM Ratio
Wilcoxon, p = 0.74
Ratio
Naive
Treated

Supplementary Figure 6.

A. Volcano plot of differentially expressed genes between treated and treatment-naive cancer cells (Wilcoxon rank-sum test with Bonferroni correction).

B. Dotplot of top differentially expressed individual genes by sample.

C. Representative images (n=11 naive, 6 treated) of multiplex immunofluorescence from cases high and low in *TFF3* at two different magnifications (upper panel P05 (naive), lower panel P14 (treated)). High power images correspond to demarcated areas in low power images. Channels (always including DAPI (blue)): CK19 (cytokeratin 19) (violet), TFF3 (Trefoil Factor 3) (green) merged and individual channels depicted in white. Scale bar = 100 µm.

D. Basal and classical signature expression scatterplot of cancer cells (treated, naive and combined) in the confirmatory dataset.

E. Basal and classical signature expression scatterplot of cancer cells (treated and naïve by patient, combined by treatment) in the confirmatory dataset; sample HT185P1 has been removed from the combined plot.

F. Gene set enrichment analysis of overall cancer cells, treated and untreated, in the confirmatory dataset.

G. Ratio of CAF (n=9 naive, 6 treated) and TAM (n=11 naive, 5 treated) subpopulations in treated and untreated groups (two-sided Wilcoxon rank-sum test; box plots centered around the median with hinges at 1st and 3rd quartiles and whiskers from hinge to max value or 1.5*IQR, whichever is smallest).

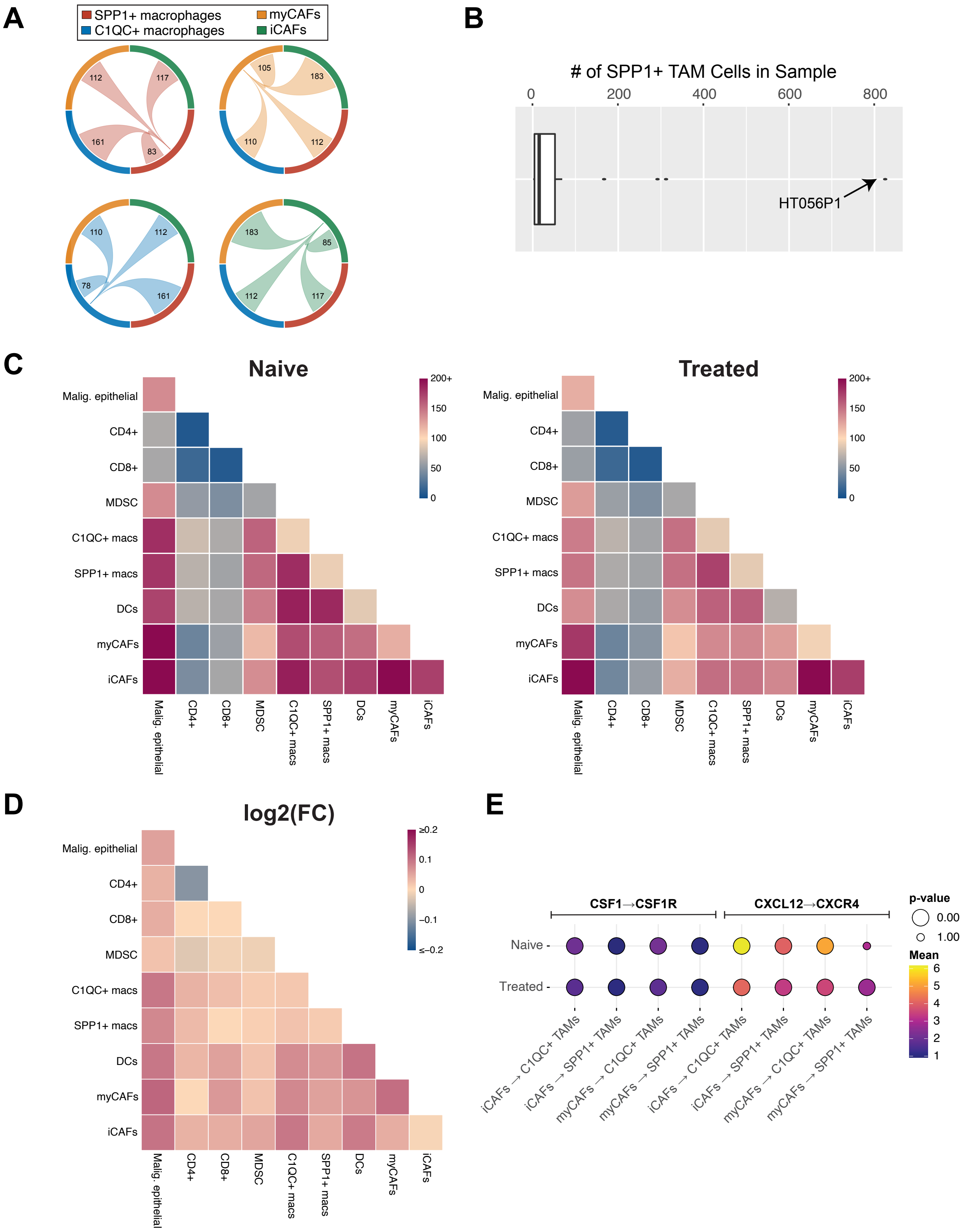
A
SPP1+ macrophages
C1QC+ macrophages
myCAFs
iCAFs
B
# of SPP1+ TAM Cells in Sample
0
200
400
600
800
HT056P1
C
Naive
Treated
Malig. epithelial
CD4+
CD8+
MDSC
C1QC+ macs
SPP1+ macs
DCs
myCAFs
iCAFs
200+
150
100
50
0
D
log2(FC)
≥0.2
0.1
0
−0.1
≤−0.2
E
CSF1→CSF1R
CXCL12→CXCR4
Naive
Treated
iCAFs → C1QC+ TAMs
iCAFs → SPP1+ TAMs
myCAFs → C1QC+ TAMs
myCAFs → SPP1+ TAMs
p-value
0.00
1.00
Mean
6
5
4
3
2
1

F

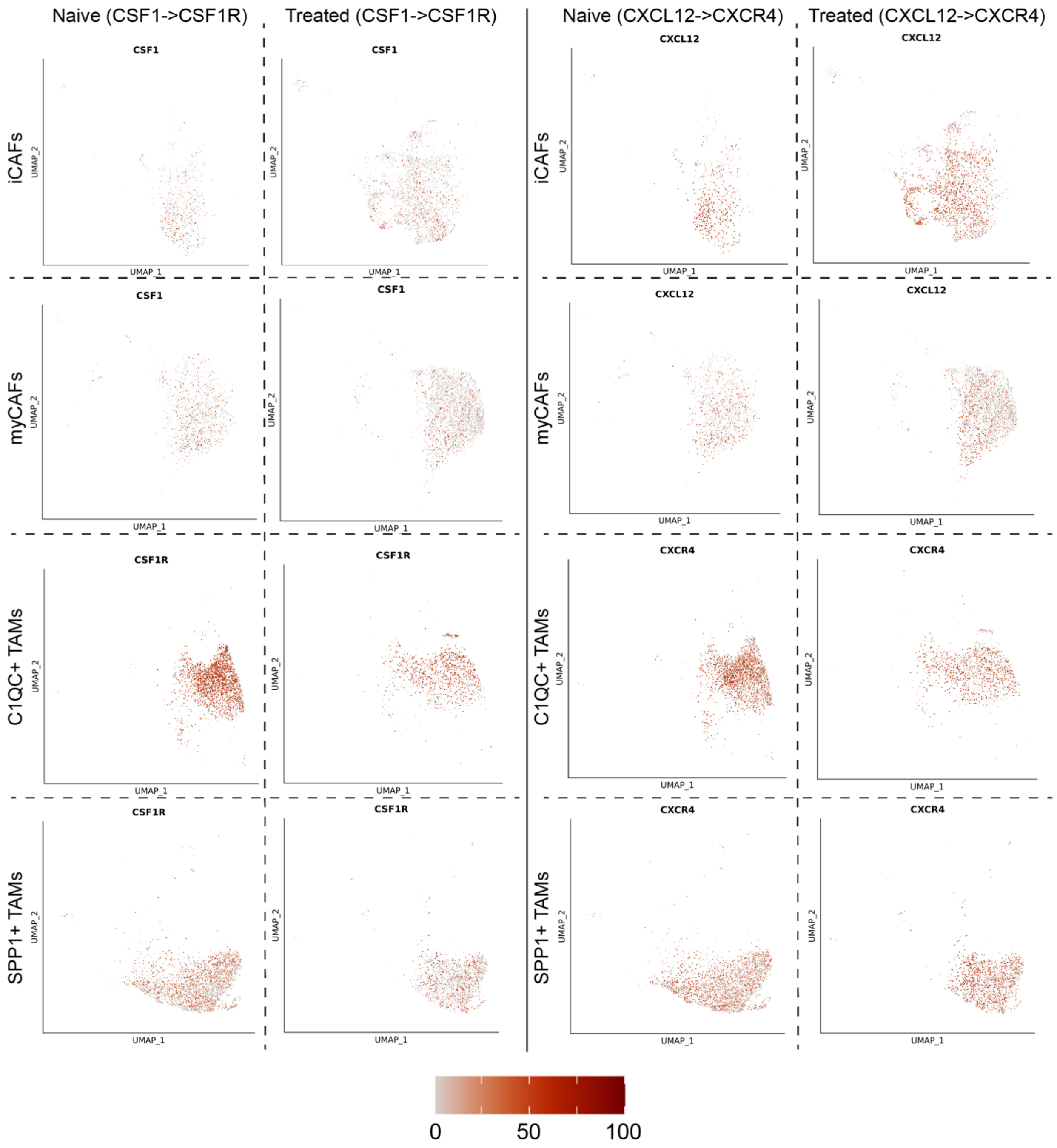

Naive (CSF1->CSF1R)
Treated (CSF1->CSF1R)
Naive (CXCL12->CXCR4)
Treated (CXCL12->CXCR4)
iCAFs
myCAFs
C1QC+ TAMs
SPP1+ TAMs
CSF1
CSF1R
CXCL12
CXCR4
UMAP_1
UMAP_2
0
50
100

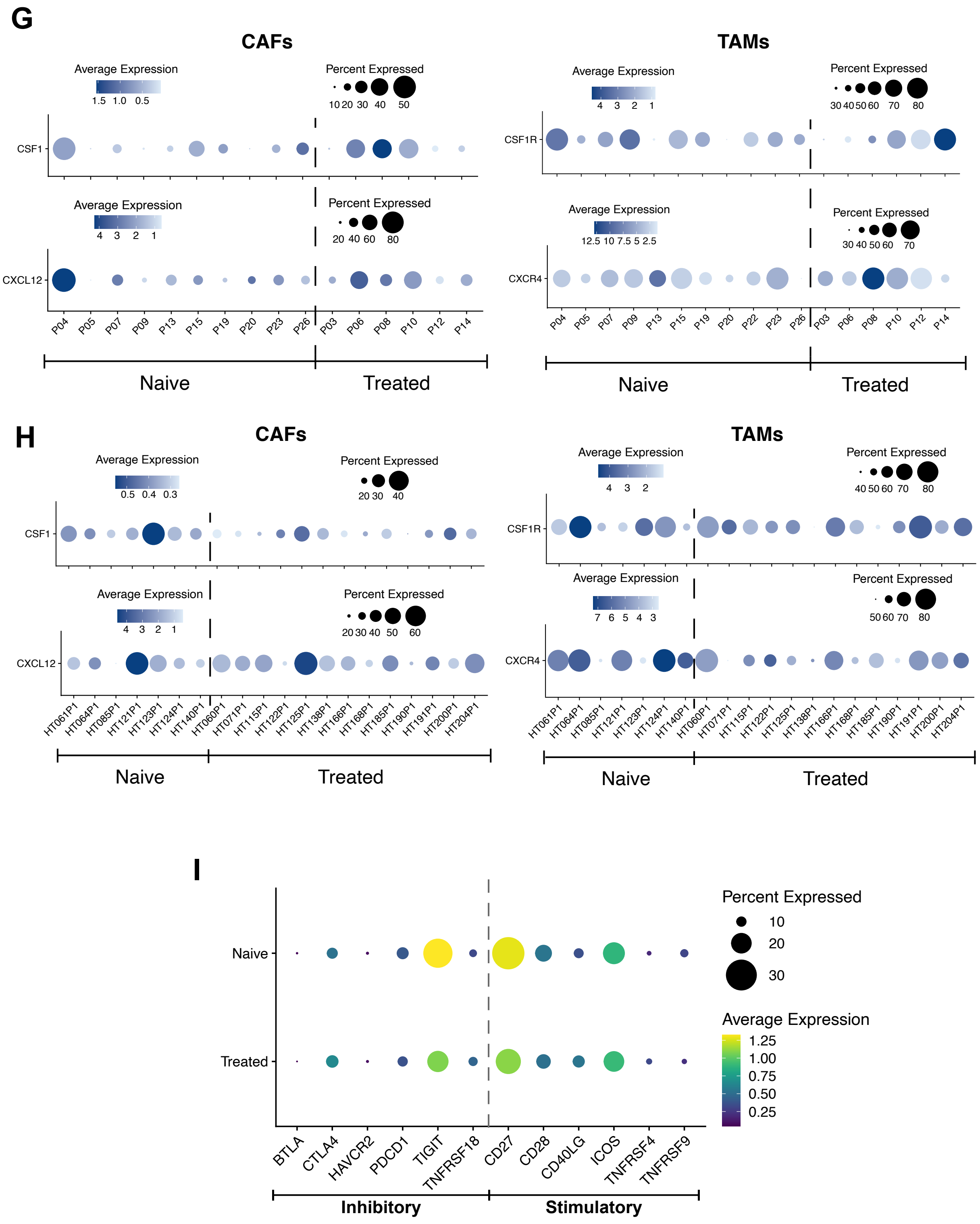
G
CAFs
TAMs
Average Expression
Percent Expressed
CSF1
CXCL12
CSF1R
CXCR4
P04
P05
P07
P09
P13
P15
P19
P20
P23
P26
P03
P06
P08
P10
P12
P14
P22
Naive
Treated
H
CAFs
TAMs
HT061P1
HT064P1
HT085P1
HT121P1
HT123P1
HT124P1
HT140P1
HT060P1
HT071P1
HT115P1
HT122P1
HT125P1
HT138P1
HT166P1
HT168P1
HT185P1
HT190P1
HT191P1
HT200P1
HT204P1
Naive
Treated
I
Naive
Treated
BTLA
CTLA4
HAVCR2
PDCD1
TIGIT
TNFRSF18
CD27
CD28
CD40LG
ICOS
TNFRSF4
TNFRSF9
Inhibitory
Stimulatory
Percent Expressed
10
20
30
Average Expression
1.25
1.00
0.75
0.50
0.25

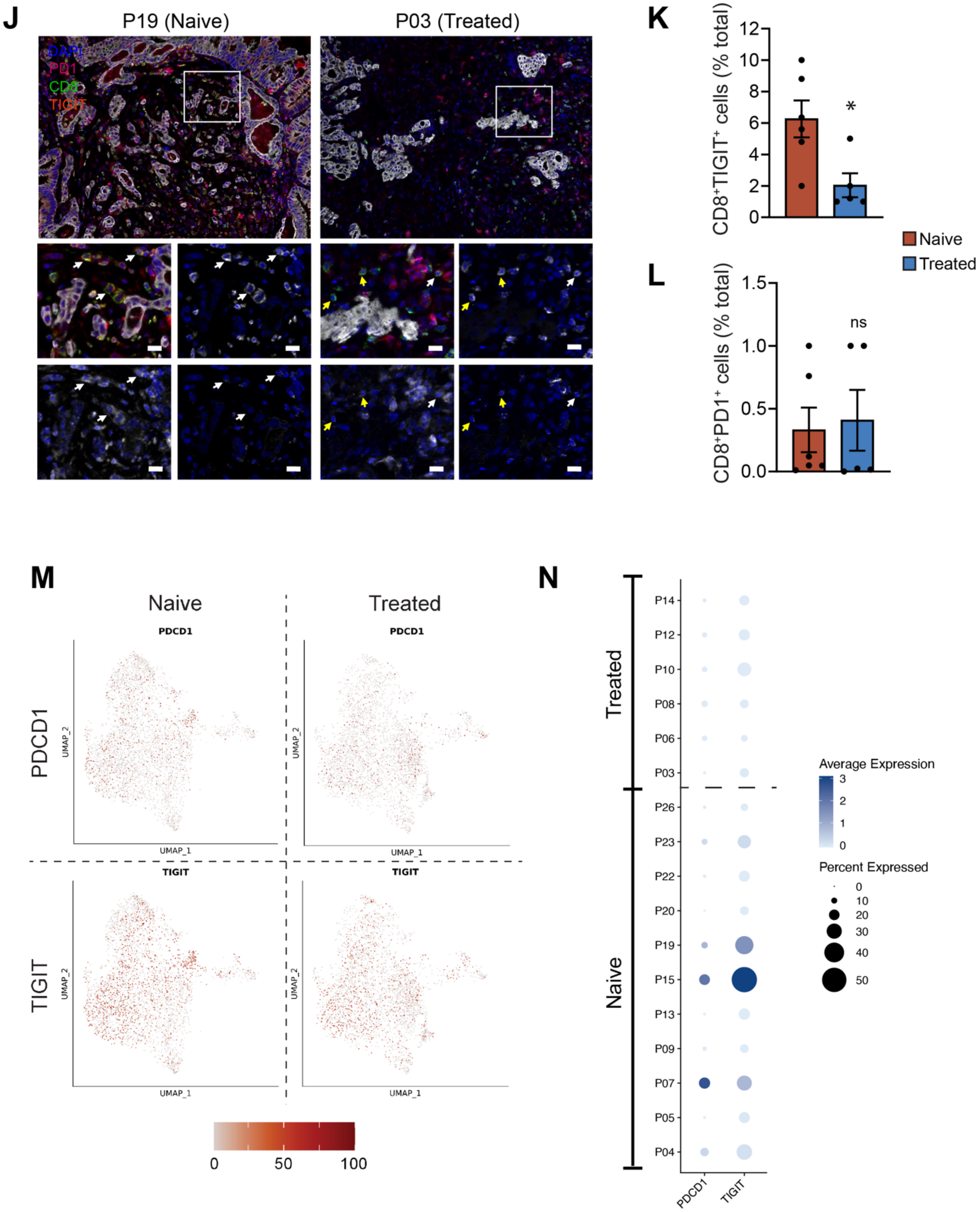
J
P19 (Naive)
P03 (Treated)
DAPI
PD1
CD8
TIGIT
K
CD8+TIGIT+ cells (% total)
*
Naive
Treated
L
CD8+PD1+ cells (% total)
ns
M
Naive
Treated
PDCD1
TIGIT
UMAP_1
UMAP_2
0
50
100
N
Treated
Naive
P14
P12
P10
P08
P06
P03
P26
P23
P22
P20
P19
P15
P13
P09
P07
P05
P04
PDCD1
TIGIT
Average Expression
Percent Expressed

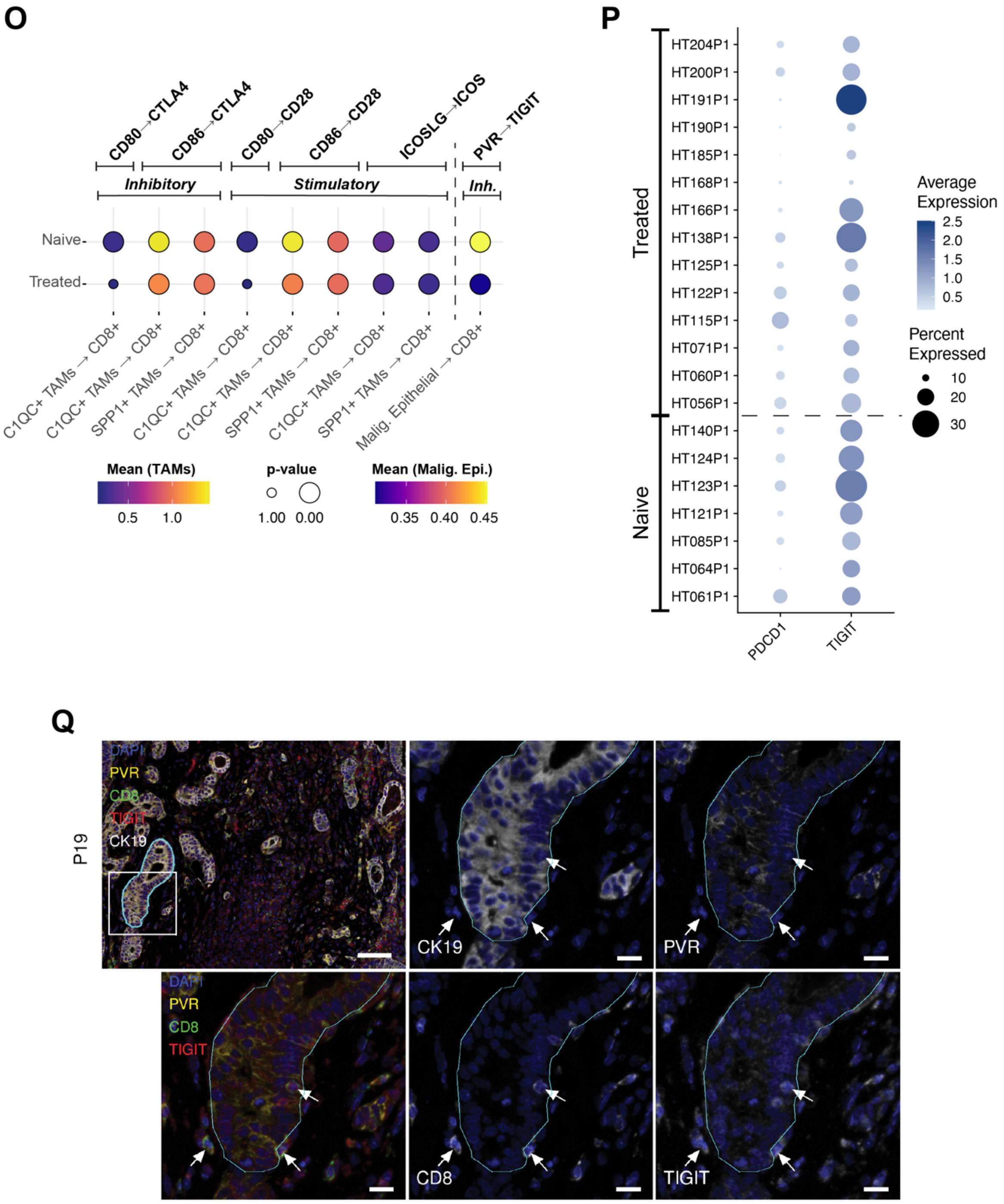

Supplementary Figure 7.

A. Interaction network for *SPP1*+ and *C1QC*+ TAMs, iCAFs and myCAFs.

B. The number of *SPP1*+ TAMs in each sample in the confirmatory dataset[8]. HT056P1 is an extreme outlier. (n=21)

C. Heatmap of all inferred LRIs between displayed cell types (cancer cells, MDSCs, DCs, *C1QC*+ TAMs, *SPP1*+ TAMs, iCAFs, myCAFs, *CD8*+ T cells, *CD4*+ T cells) in the

confirmatory dataset. Left: untreated samples, right: treated samples. CAF, cancer-associated fibroblast; DC, dendritic cell; iCAF, inflammatory CAF; LRI, ligand-receptor interaction; MDSC, myeloid-derived suppressor cell; myCAF, myofibroblastic CAF; TAM, tumor-associated macrophage.

D. Heatmap displaying the fold change between the number of inferred LRIs in naïve and treated samples for each pair of cell types in the confirmatory dataset. Red: more inferred LRIs in naïve samples; blue: more inferred LRIs in treated samples.

E. Dotplot of CellphoneDB output (see Methods) for recruitment-associated LRIs between iCAFs/myCAFs and *SPP1*+/*C1QC*+ TAMs comparing untreated and treated samples in the confirmatory dataset.

F. UMAPs showing expression of recruitment-related LRI genes for iCAFs/myCAFs and *SPP1*+/*C1QC*+ TAMs in untreated and treated samples.

G. Dotplot of recruitment-associated LRI gene expression between CAFs and TAMs per sample in our dataset.

H. Dotplot of recruitment-associated LRI gene expression between CAFs and TAMs per sample in the confirmatory dataset.

I. Differential gene expression of checkpoint molecules in *CD8*+ T cells between treated and untreated samples in the confirmatory dataset.

J. Representative multiplex immunofluorescence images from cases high and low in *TIGIT* expression in *CD8*+ T cells at two different magnifications (upper panel P19 (naive), lower panel P03 (treated); n=11 naive, 6 treated). High power images correspond to demarcated areas in low power images. Channels: DAPI (blue), CK19 (white), TIGIT (red), PD1 (magenta), CD8 (green) merged and individual channels depicted in white. White arrowheads indicate CD8+ T cells with high TIGIT expression, yellow arrowheads indicate CD8+ T cells with low TIGIT expression. Scale bar=100µm.

K. Percent of CD8+ cells that express TIGIT in representative naïve (n=5) and treated (n=6) samples. Data are presented as mean values +/- SE. (*: p=0.02; two-tailed t-test)

L. Percent of CD8+ cells that express PD1 in representative naïve (n=5) and treated (n=6) samples. Data are presented as mean values +/- SE. (ns=not significant; two-tailed t-test)

M. UMAPs showing expression of two checkpoint inhibitory genes, *PDCD1* and *TIGIT*, in *CD8*+ T cells in untreated and treated samples.

N. Dotplot of gene expression of two checkpoint inhibitory genes, *PDCD1* and *TIGIT*, in *CD8*+ T cells by sample in our dataset.

O. Dotplot of CellphoneDB output (see Methods) for checkpoint molecule LRIs between *CD8*+ T cells, *SPP1*+, *C1QC*+ TAMs, and cancer cells comparing untreated and treated samples in the confirmatory dataset.

P. Dotplot of gene expression of two checkpoint inhibitory genes, *PDCD1* and *TIGIT*, in *CD8*+ T cells by sample in the confirmatory dataset.

Q. Representative multiplex immunofluorescence images (n=11) showing colocalization of TIGIT+ CD8+ T cells and PVR+ CK19+ cancer cells at two different magnifications (P19

(naive)). High power images correspond to demarcated areas in low power images. Channels: DAPI (blue), CK19 (white), TIGIT (red), PVR (yellow), CD8 (green) merged and individual channels depicted in white. Cyan outline marks the border of cancer cells. White arrows indicate TIGIT+ CD8+ T cells in close proximity to cancer cells. Scale bar=100µm.

Supplementary Table 1. Aggregated patient characteristics (age in years, stage by AJCC [American Joint Committee on Cancer] criteria, treatment with FFX [FOLFIRINOX] or G/A [gemcitabine/abraxane]).

**Age**		68 ± 10
**Gender**	Female	15
	Male	12
**Procedure**	Resection	10
	Biopsy	17
**Stage (AJCC)**	IB	6
	IIB	2
	III	5
	IV	14
**Treatment**	Naïve	20
	FFX - based	4
	G/A	3
**Mutations**	KRAS	19
	TP53	16

Supplementary Table 2. Clinical outcomes for treated patients.

Patient	Treatment	Treatment Duration	RECIST Criteria Response
**P03**	FFX	7M	PR
**P06**	FFX, G/A	17M, 3M	PD
**P08**	G/A	4M	SD
**P10**	FFX/SBRT	5M	SD
**P11**	FFX	6M	PD
**P12**	FFX, G/A	20M, 2M	PD
**P14**	FFX	4M	PR
**P17**	FFX	3M	Non-evaluable

RECIST criteria response for chemotherapy-treated study patients with ScSeq pancreas biopsies
FFX = Folfirinox
G/A = Gemcitabine/Abraxane
SBRT = Sterotactic body radiation therapy

PD = Progressive disease
PR = Partial Response
SD = Stable Disease
*P17 was not evaluable for response as patient had initial chemotherapy at an outside hospital and outside images were not available to evaluate for RECIST measurements

Supplementary Table 3. Antibodies used for multiplex IHC.

**Moffitt Subtype Panel**							
**Target**	**Vendor/ Cat#**	**Host**	**Clone**	**Dilution**	**2 HRP-polymer**	**Fluoro-phore**	**Vendor/Cat#**
GATA6	R&D, AF1700	Goat	Poly	1:200	Biocare Med, GHP516	Opal 480	Akoya FP1500001KT
CK17	Proteintech 17516-1-AP	Rabbit	Poly	1:100	Akoya ARH1001	Opal 520	Akoya FP1487001KT
CK19	Biocare Med, CD242A	Mouse	Ks19.1	1:100	Akoya ARH1001	Opal 780	Akoya FP1501001KT TSA-DIG & OP780
**Checkpoint Panel**							
**Target**	**Vendor/ Cat#**	**Host**	**Clone**	**Dilution**	**2 HRP-polymer**	**Fluoro-phore**	**Vendor/Cat#**
CD8	Dako, M710301-2 / M7103	Mouse	C8/144B	1:100	Akoya ARH1001	Opal 620	Akoya FP1495001KT
TIGIT	Abcam, ab243903	Rabbit	BLR047F	1:200	Akoya ARH1001	Opal 690	Akoya FP1497001KT
PD-1	CST, 86163S	Rabbit	D4W2J	1:200	Akoya ARH1001	Opal 520	Akoya FP1487001KT
PVR	CST, 81254S	Rabbit	D8A5G	1:300	Akoya ARH1001	Opal 480	Akoya FP1500001KT
CK19	Biocare Med, CD242A	Mouse	Ks19.1	1:100	Akoya ARH1001	Opal 780	Akoya FP1501001KT TSA-DIG & OP780
**Cancer DEG Panel**							
**Target**	**Vendor/ Cat#**	**Host**	**Clone**	**Dilution**	**2 HRP-polymer**	**Fluoro-phore**	**Vendor/Cat#**
TFF3 (ITF)	Santa Cruz, sc-398651	Mouse	B-1	1:400	Akoya ARH1001	Opal 520	Akoya FP1487001KT
CK19	Biocare Med, CD242A	Mouse	Ks19.1	1:100	Akoya ARH1001	Opal 620	Akoya FP1495001KT

Supplementary References

1. Raghavan, S. *et al.* Microenvironment drives cell state, plasticity, and drug response in pancreatic cancer. *Cell* **184**, 6119–6137.e26 (2021).
2. Moffitt, R. A. *et al.* Virtual microdissection identifies distinct tumor- and stroma-specific subtypes of pancreatic ductal adenocarcinoma. *Nat. Genet.* **47**, 1168–1178 (2015).
3. Collisson, E. A. *et al.* Subtypes of pancreatic ductal adenocarcinoma and their differing responses to therapy. *Nat. Med.* **17**, 500–503 (2011).
4. Bailey, P. *et al.* Genomic analyses identify molecular subtypes of pancreatic cancer. *Nature* **531**, 47–52 (2016).
5. Puleo, F. *et al.* Stratification of Pancreatic Ductal Adenocarcinomas Based on Tumor and Microenvironment Features. *Gastroenterology* **155**, 1999–2013.e3 (2018).
6. Puram, S. V. *et al.* Single-Cell Transcriptomic Analysis of Primary and Metastatic Tumor Ecosystems in Head and Neck Cancer. *Cell* **171**, 1611–1624.e24 (2017).
7. Cancer Genome Atlas Research Network. Integrated Genomic Characterization of Pancreatic Ductal Adenocarcinoma. *Cancer Cell* **32**, 185–203.e13 (2017).
8. Cui Zhou, D. *et al.* Spatially restricted drivers and transitional cell populations cooperate with the microenvironment in untreated and chemo-resistant pancreatic cancer. *Nat. Genet.* **54**, 1390–1405 (2022).
9. Elyada, E. *et al.* Cross-Species Single-Cell Analysis of Pancreatic Ductal Adenocarcinoma Reveals Antigen-Presenting Cancer-Associated Fibroblasts. *Cancer Discov.* **9**, 1102–1123 (2019).
10. Li, H. *et al.* Dysfunctional CD8 T cells form a proliferative, dynamically regulated compartment within human melanoma. *Cell* **176**, 775–789.e18 (2019).
11. Azizi, E. *et al.* Single-Cell Map of Diverse Immune Phenotypes in the Breast Tumor

Microenvironment. *Cell* **174**, 1293–1308.e36 (2018).

12. Cheng, H.-W. *et al.* CCL19-producing fibroblastic stromal cells restrain lung carcinoma growth by promoting local antitumor T-cell responses. *J. Allergy Clin. Immunol.* **142**, 1257–1271.e4 (2018).
