# Supplementary material for: Single-cell RNA sequencing reveals the effects of chemotherapy on human pancreatic adenocarcinoma and its tumor microenvironment
Source: Nat Commun. 2023 Feb 13;14:797. doi: 10.1038/s41467-023-36296-4 (PMC9925748; doi:10.1038/s41467-023-36296-4)
Supplement: Supplementary file 2 — Description of Additional Supplementary Files [file 41467_2023_36296_MOESM2_ESM.pdf]

File Name: Supplementary Data 1
Description: Individual genomic mutations per sample.

File Name: Supplementary Data 2
Description: Individual clinical information, corresponding H&E staining at low (scale bar: 1 mm) and high power (scale bar: 50 µm) magnification, and UMAPs of all cells that passed quality control.

File Name: Supplementary Data 3
Description: Gene signatures used in Figures 2, 3, 4, 5, and Supplementary Figures 2 & 3.

File Name: Supplementary Data 4
Description: Differential expression used for volcano plots in Supplementary Figures 3D, 5C, 6A.

File Name: Supplementary Data 5
Description: CPDB output (significant_means.txt) for treatment-naive and treated primary pancreatic tumors.
