# Supplementary material for: Single-cell RNA sequencing reveals the effects of chemotherapy on human pancreatic adenocarcinoma and its tumor microenvironment
Source: Nat Commun. 2023 Feb 13;14:797. doi: 10.1038/s41467-023-36296-4 (PMC9925748; doi:10.1038/s41467-023-36296-4)

**Patient Number**	P01
**Age**	78
**Gender**	Male
**Stage at Diagnosis**	IV
**Treatment before tissue collection**	No
**Tissue site**	Liver
**Procedure**	Biopsy
**Pathology**	Moderately differentiated adenocarcinoma
**Mutations**	KRAS G12R, TP53 Y327*

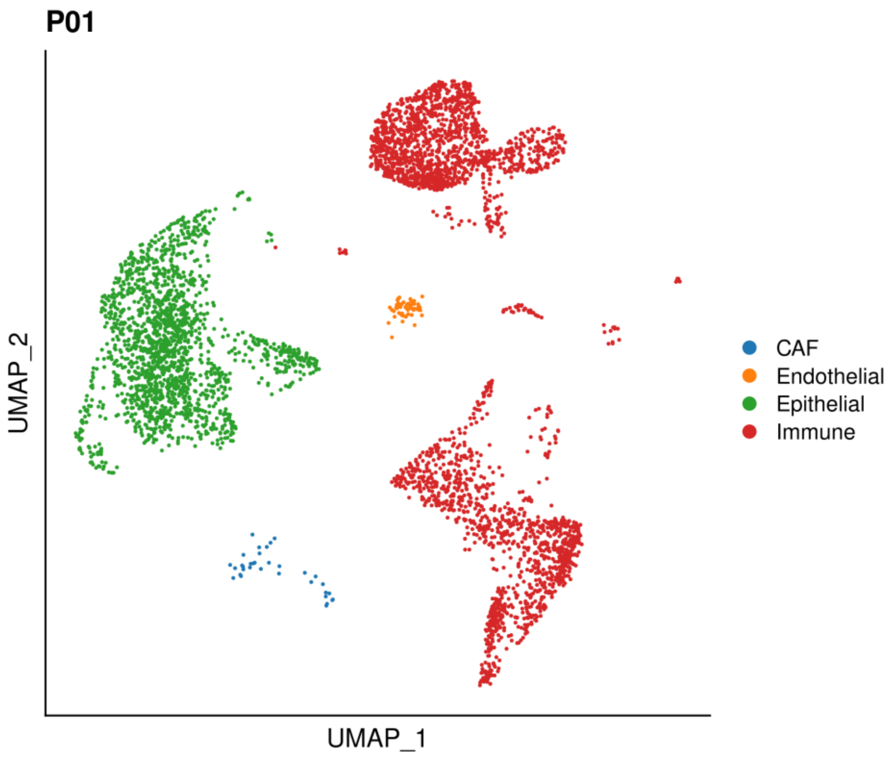

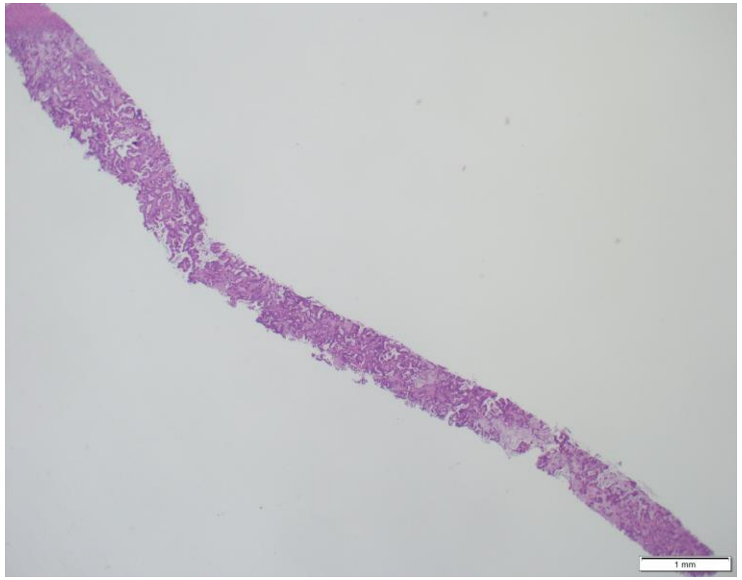

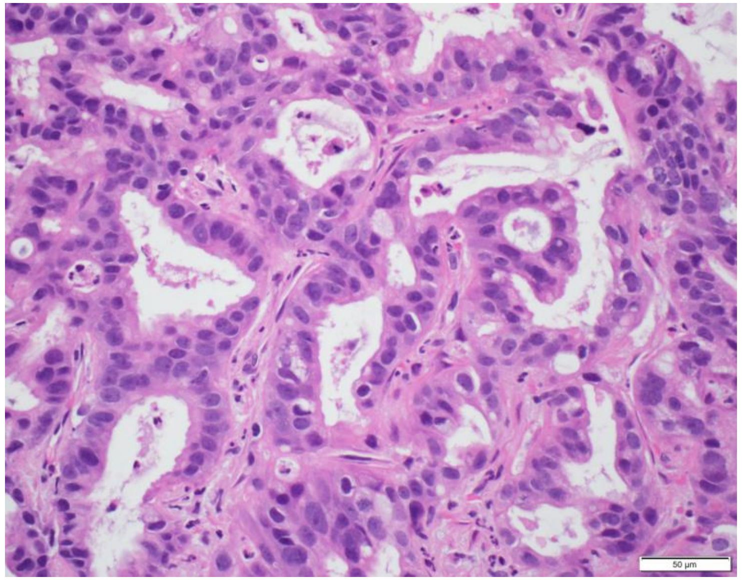

**Patient Number**	P02
**Age**	74
**Gender**	Male
**Stage at Diagnosis**	IV
**Treatment before tissue collection**	No
**Tissue site**	Liver
**Procedure**	Biopsy
**Pathology**	Moderately differentiated adenocarcinoma
**Mutations**	KRAS G12D, TP53 c.673-1G>A

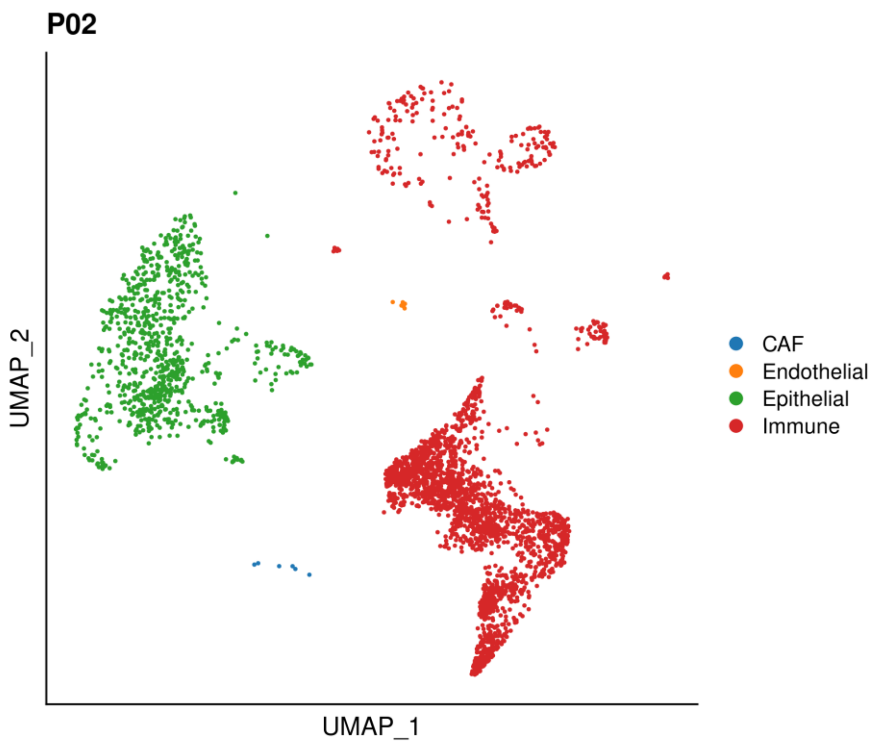

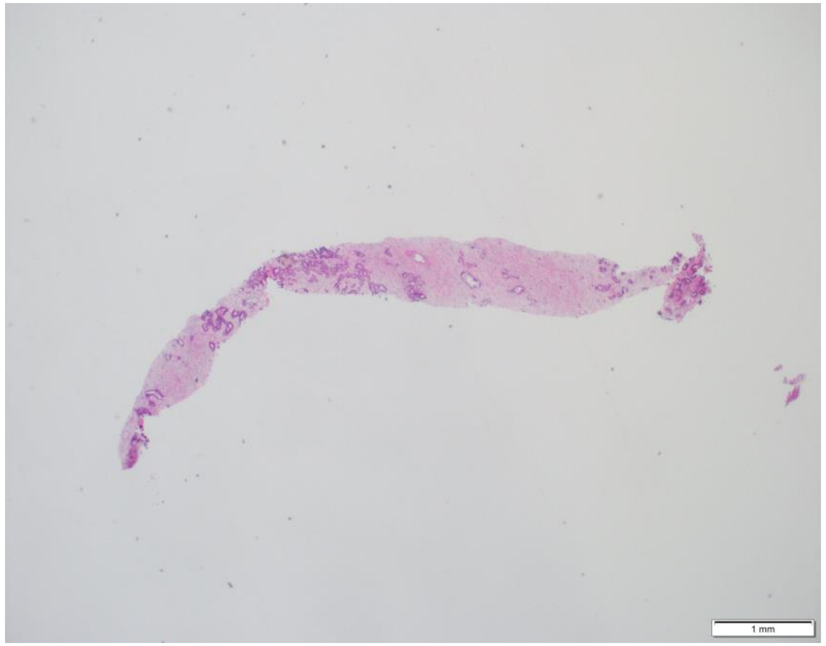

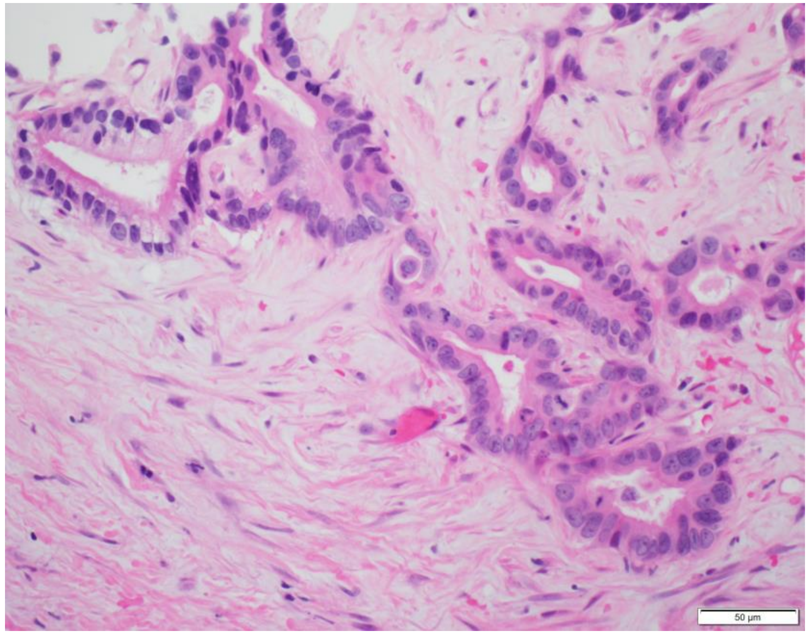

**Patient Number**	P03
**Age**	81
**Gender**	Male
**Stage at Diagnosis**	IV
**Treatment before tissue collection**	Yes
**Therapeutics**	FFX-based
**Tissue site**	Pancreas
**Procedure**	Resection
**Pathology**	Poorly differentiated adenocarcinoma
**Mutations**	WT

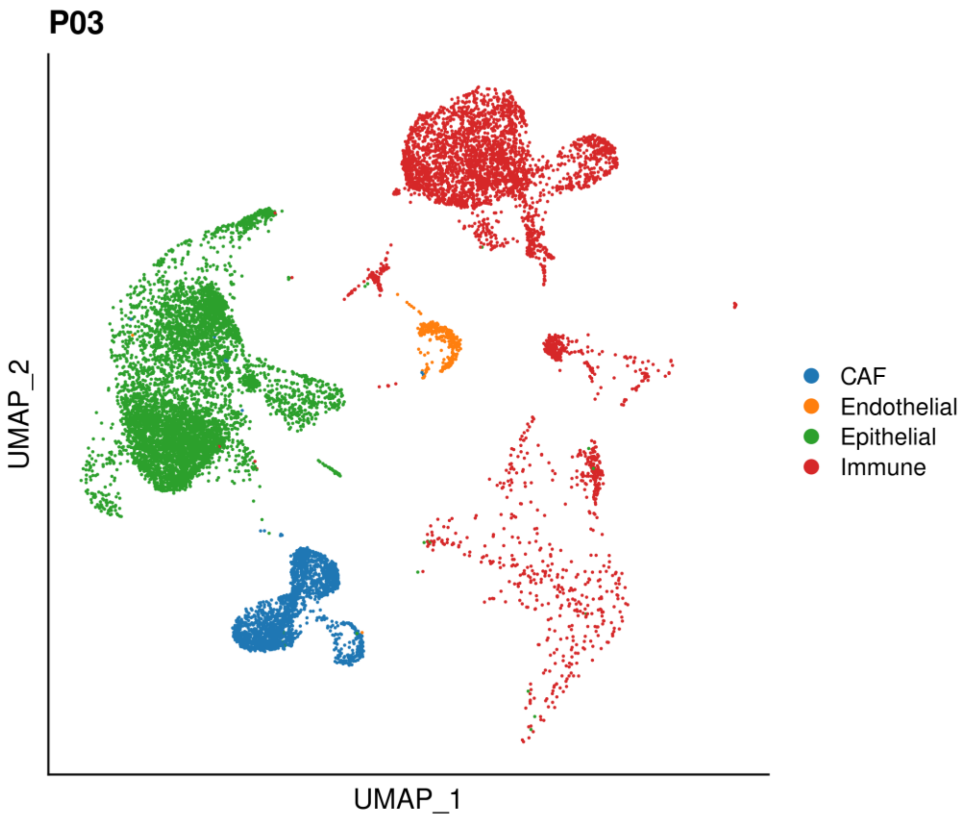

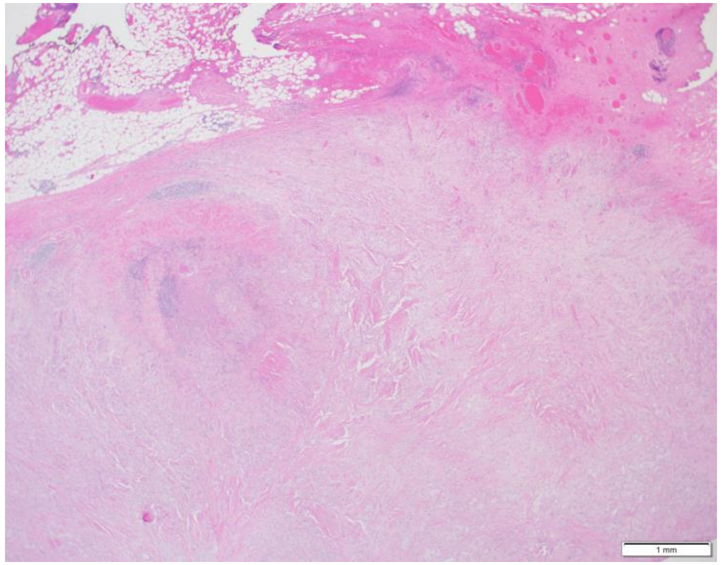

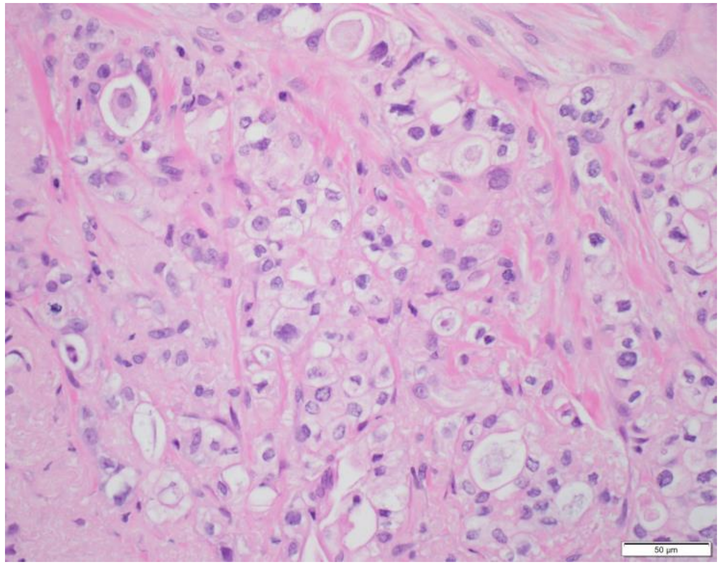

**Patient Number**	P04
**Age**	85
**Gender**	Female
**Stage at Diagnosis**	IB
**Treatment before tissue collection**	No
**Tissue site**	Pancreas
**Procedure**	Resection
**Pathology**	Moderately to poorly differentiated adenocarcinoma
**Mutations**	KRAS G12D, TP53 V147G

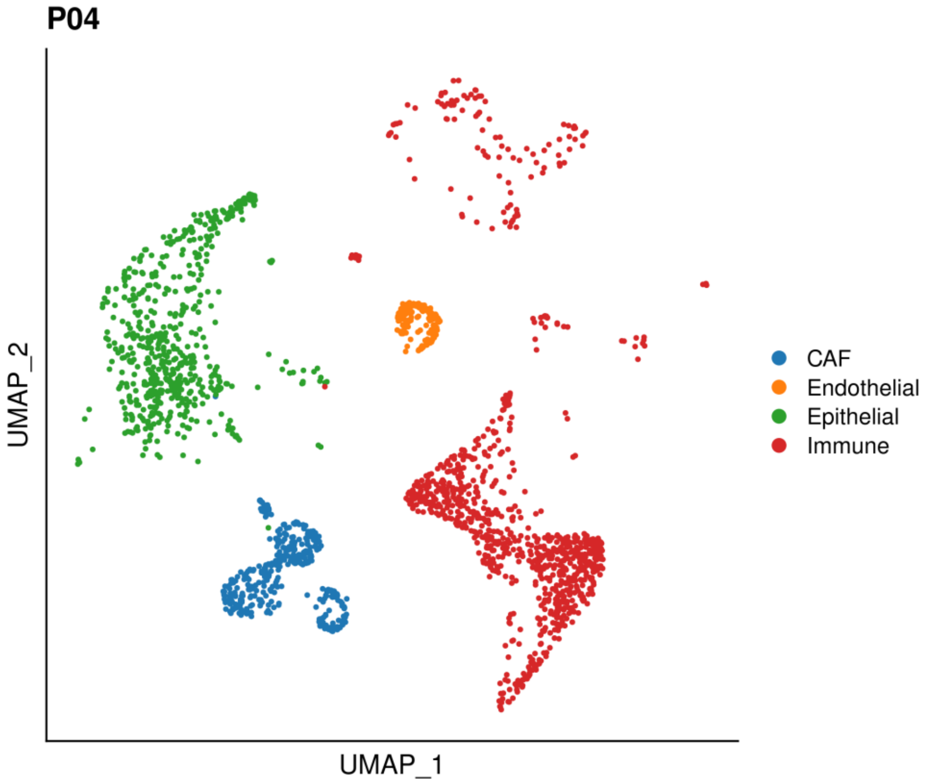

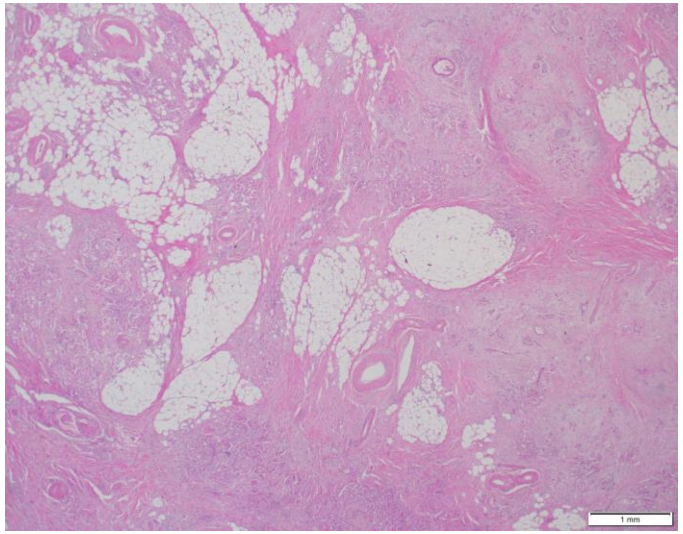

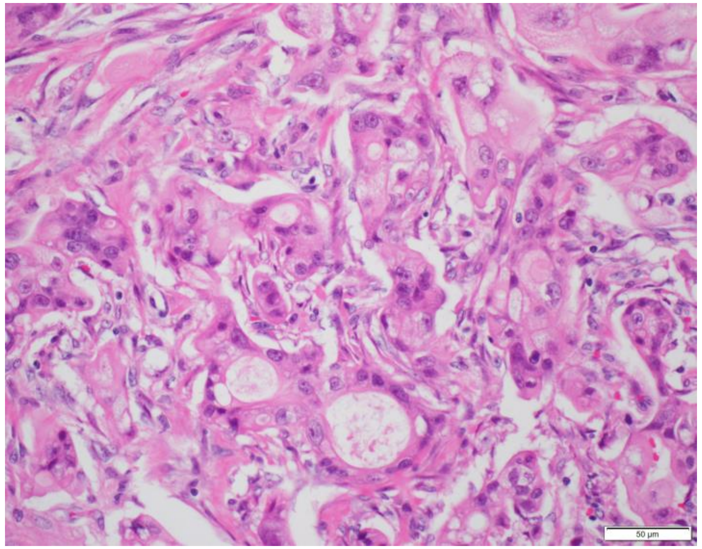

**Patient Number**	P05
**Age**	69
**Gender**	Female
**Stage at Diagnosis**	III
**Treatment before tissue collection**	No
**Tissue site**	Pancreas
**Procedure**	Resection
**Pathology**	Moderately differentiated adenocarcinoma
**Mutations**	KRAS G12D, TP53 H193L, SMAD4 R135*

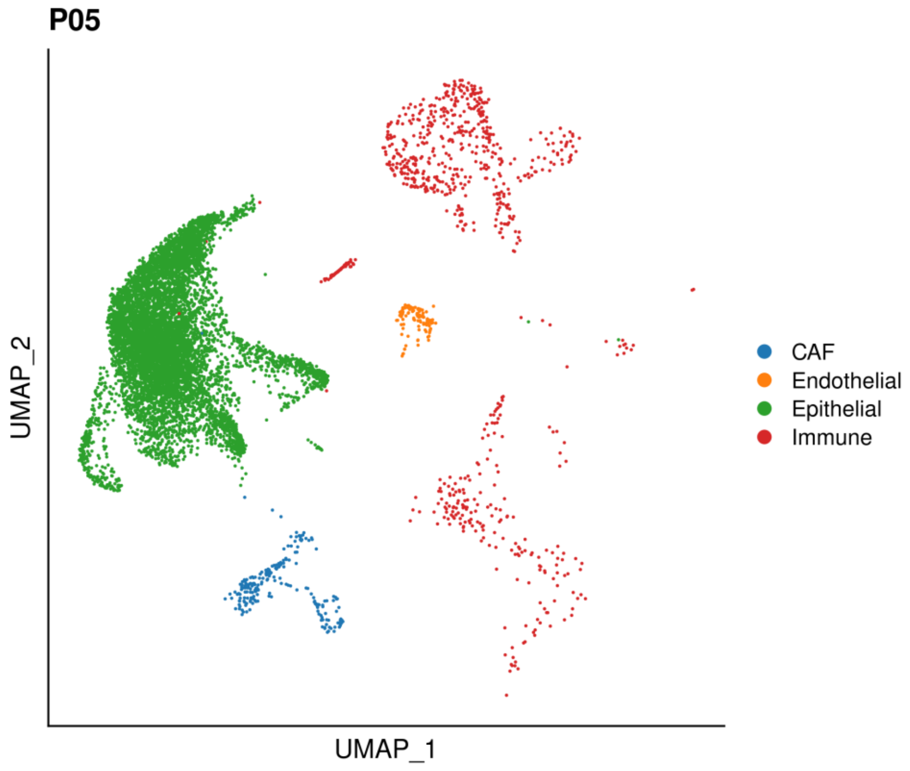

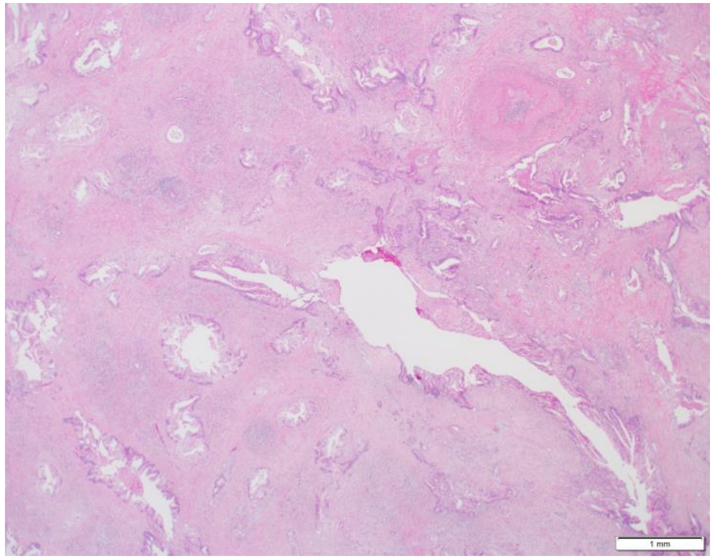

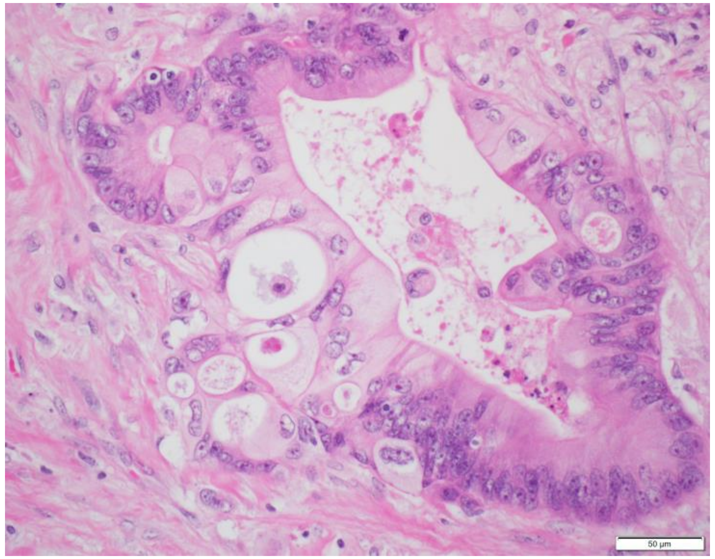

**Patient Number**	P06
**Age**	55
**Gender**	Male
**Stage at Diagnosis**	III
**Treatment before tissue collection**	Yes
**Therapeutics**	G/A
**Tissue site**	Pancreas
**Procedure**	Biopsy
**Pathology**	Moderately to poorly differentiated adenocarcinoma
**Mutations**	N/A

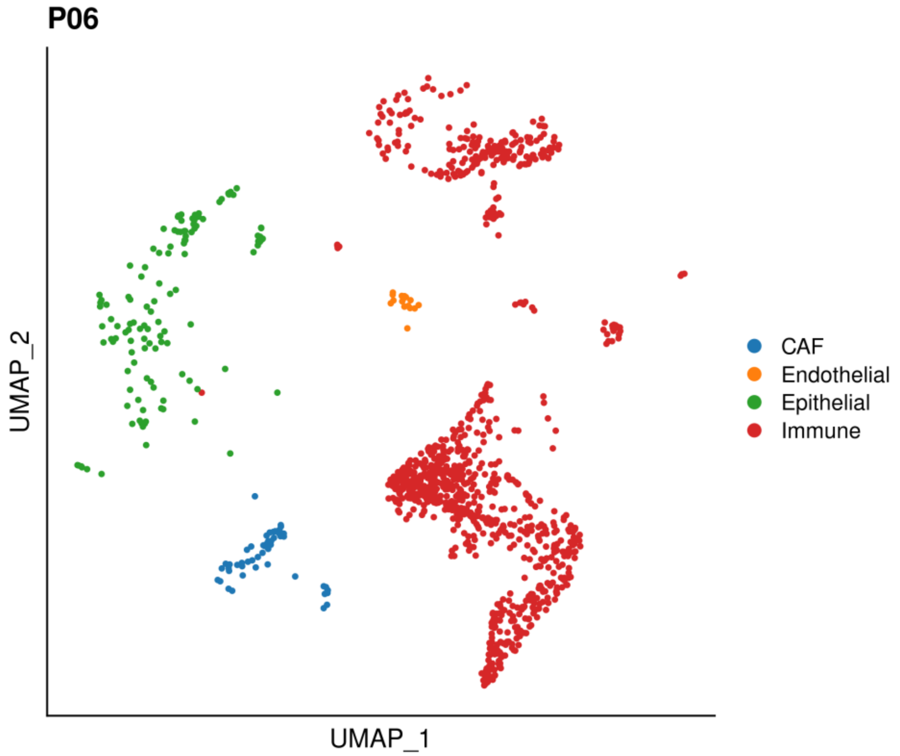

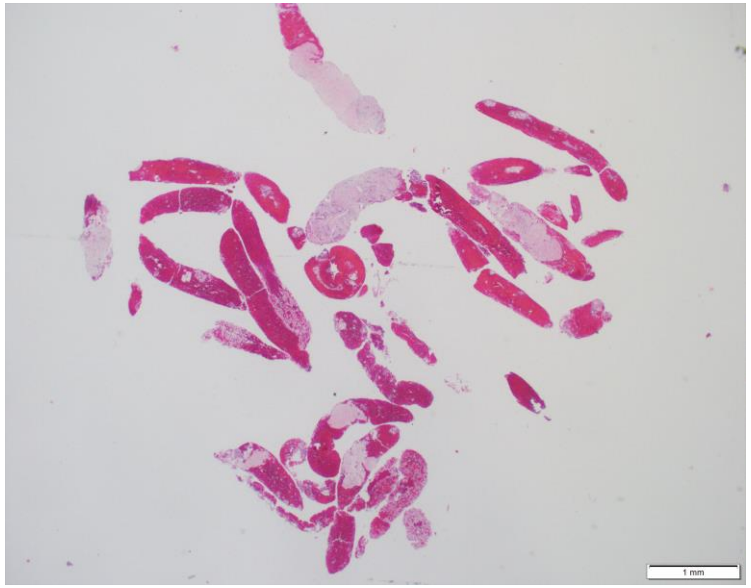

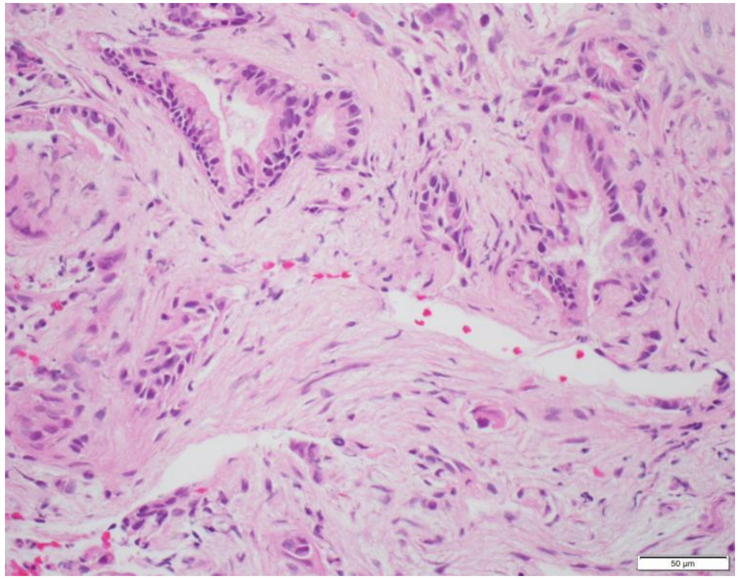

**Patient Number**	P07
**Age**	70
**Gender**	Female
**Stage at Diagnosis**	IB
**Treatment before tissue collection**	No
**Tissue site**	Pancreas
**Procedure**	Resection
**Pathology**	Well to moderately differentiated adenocarcinoma
**Mutations**	KRAS G12V

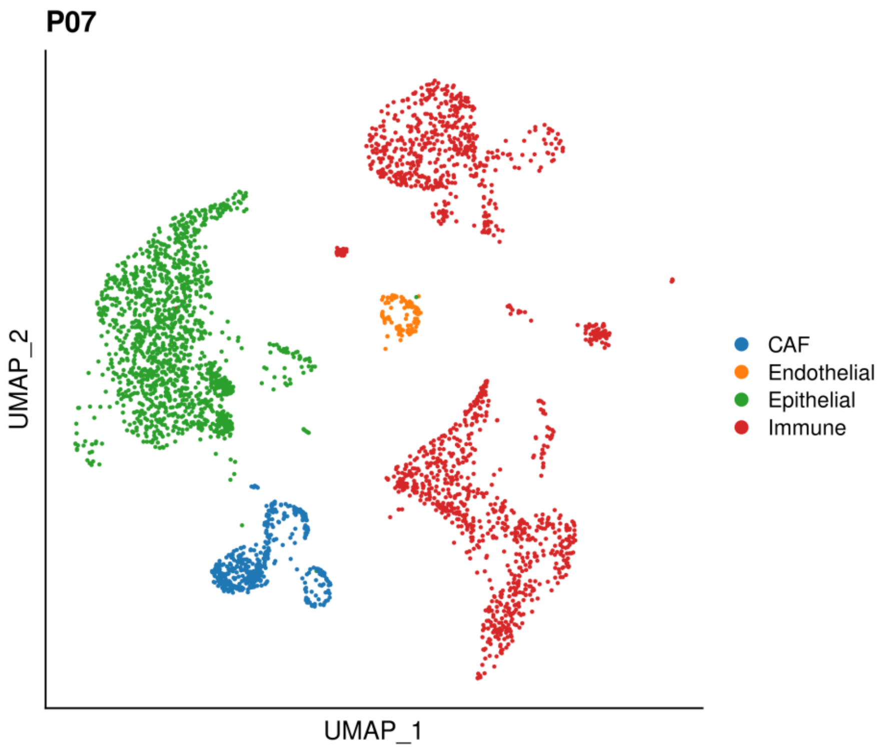

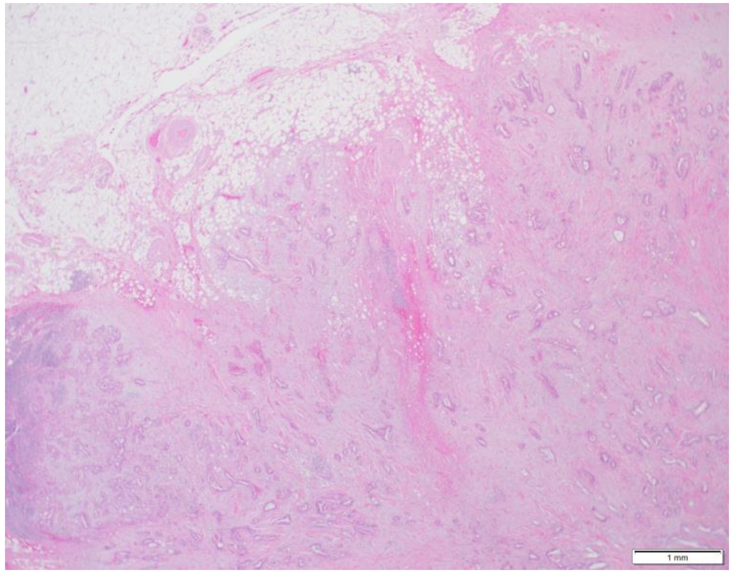

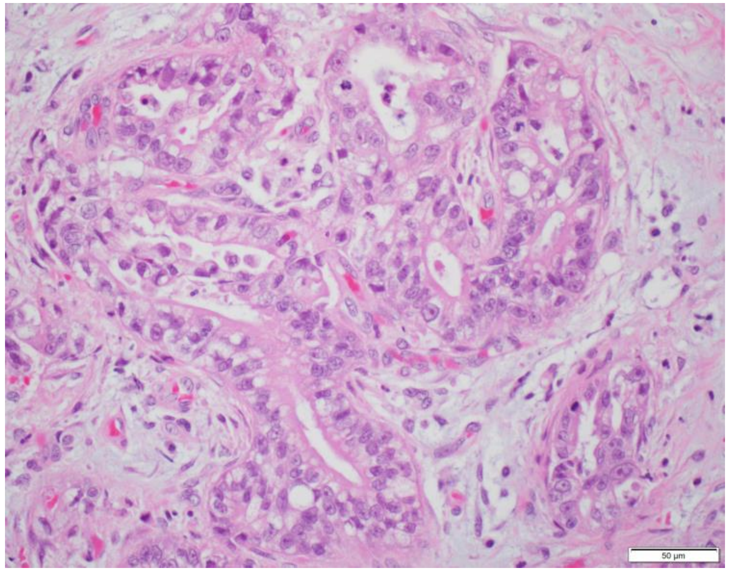

**Patient Number**	P08
**Age**	87
**Gender**	Female
**Stage at Diagnosis**	IIB
**Treatment before tissue collection**	Yes
**Therapeutics**	G/A
**Tissue site**	Pancreas
**Procedure**	Resection
**Pathology**	Moderately differentiated adenocarcinoma
**Mutations**	KRAS G12D, TP53 C141Y

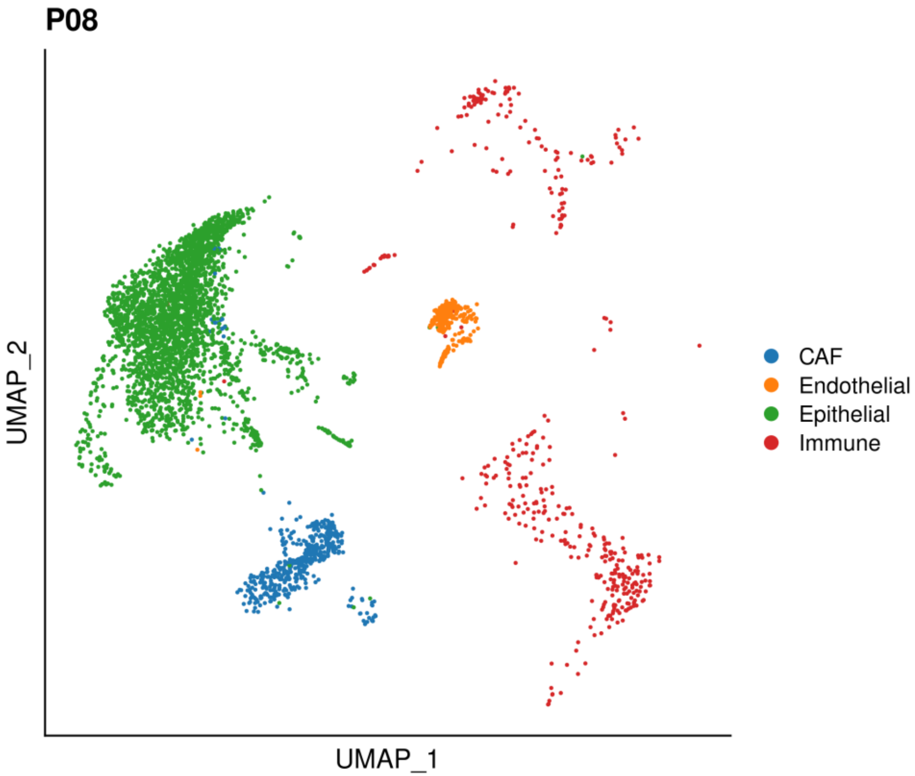

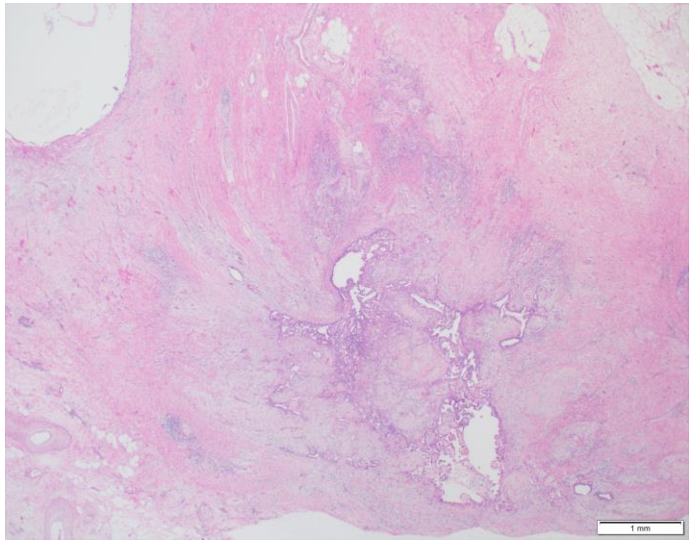

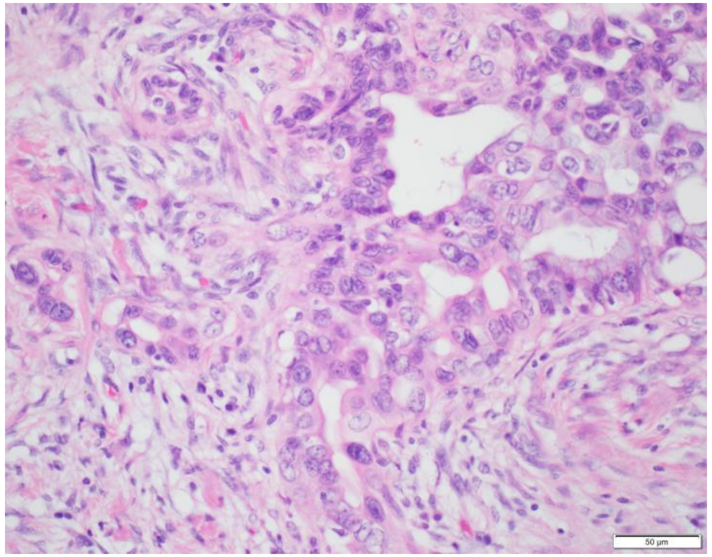

**Patient Number**	P09
**Age**	63
**Gender**	Male
**Stage at Diagnosis**	IV
**Treatment before tissue collection**	No
**Tissue site**	Pancreas
**Procedure**	Biopsy
**Pathology**	Well differentiated adenocarcinoma
**Mutations**	N/A

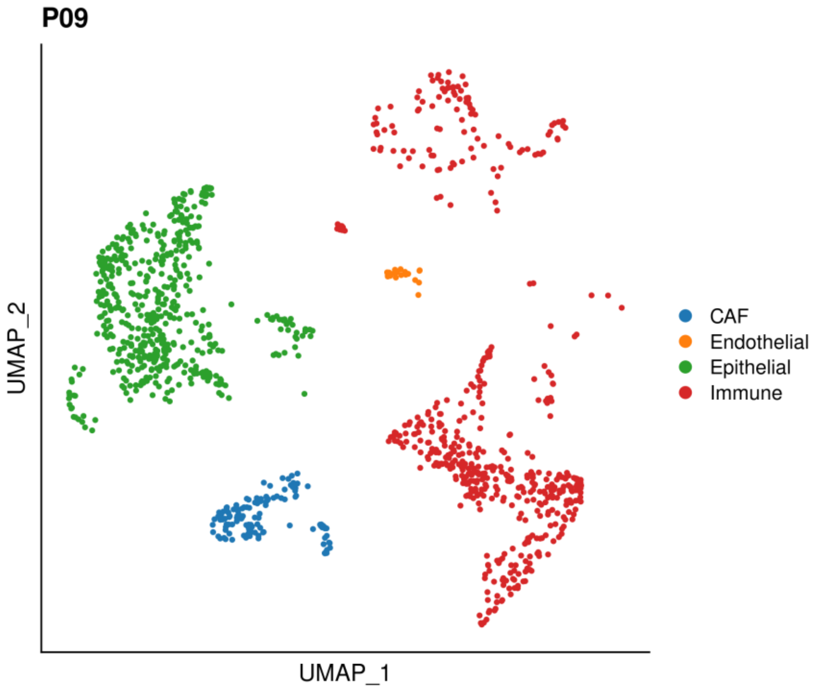

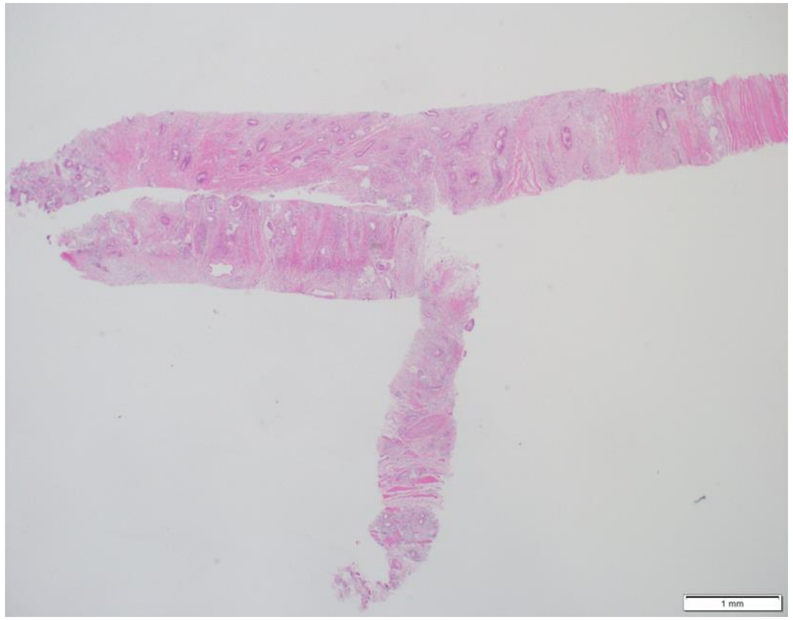

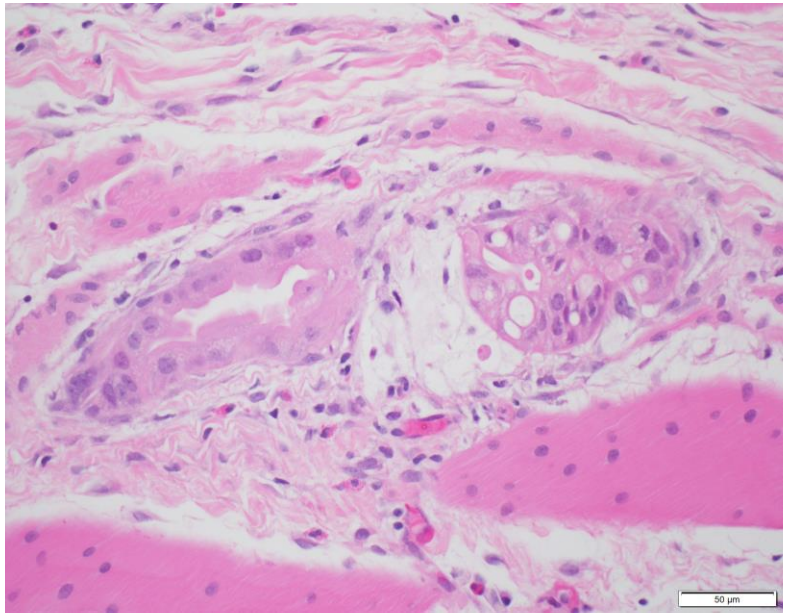

**Patient Number**	P10
**Age**	48
**Gender**	Female
**Stage at Diagnosis**	IIB
**Treatment before tissue collection**	Yes
**Therapeutics**	FFX-based
**Tissue site**	Pancreas
**Procedure**	Resection
**Pathology**	Well differentiated adenocarcinoma
**Mutations**	KRAS G12V

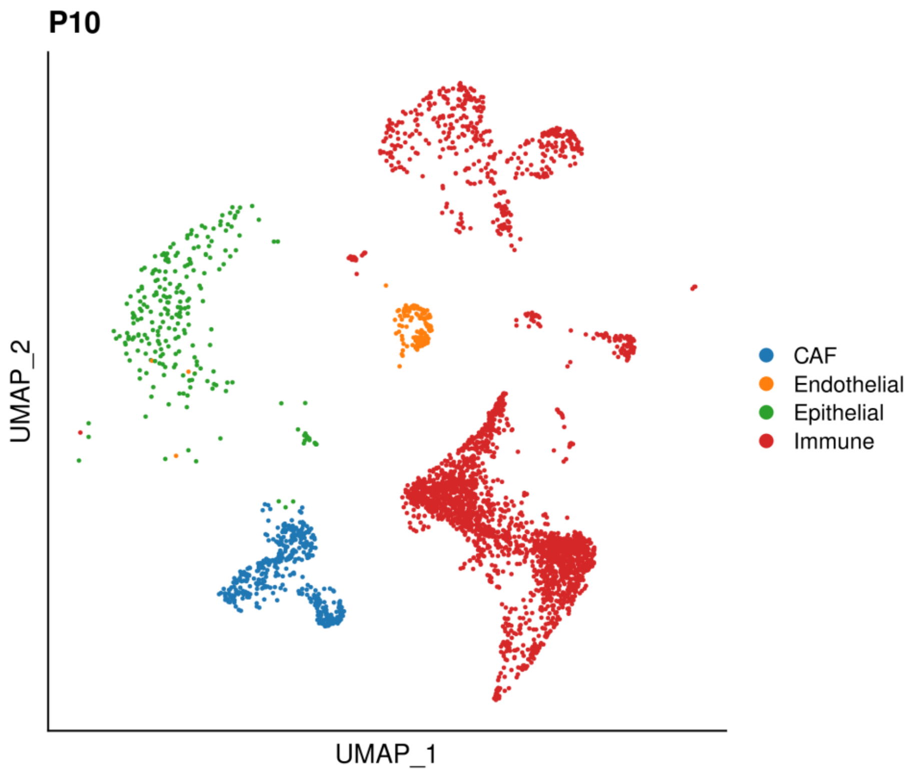

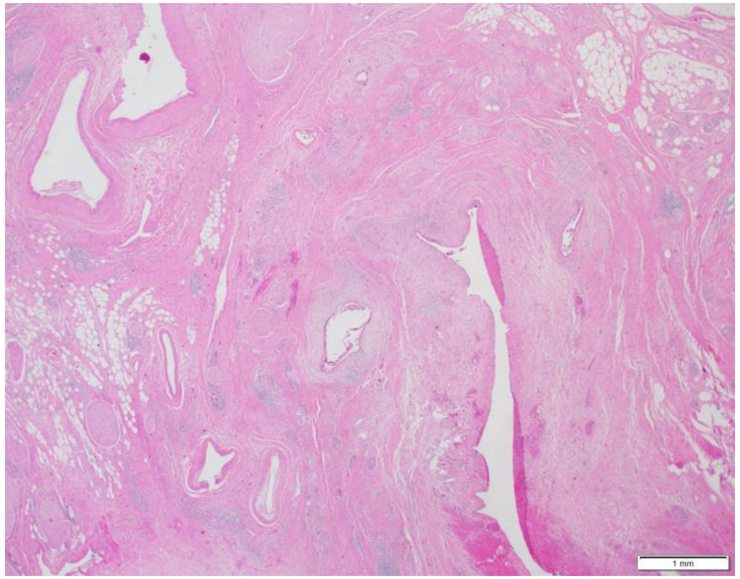

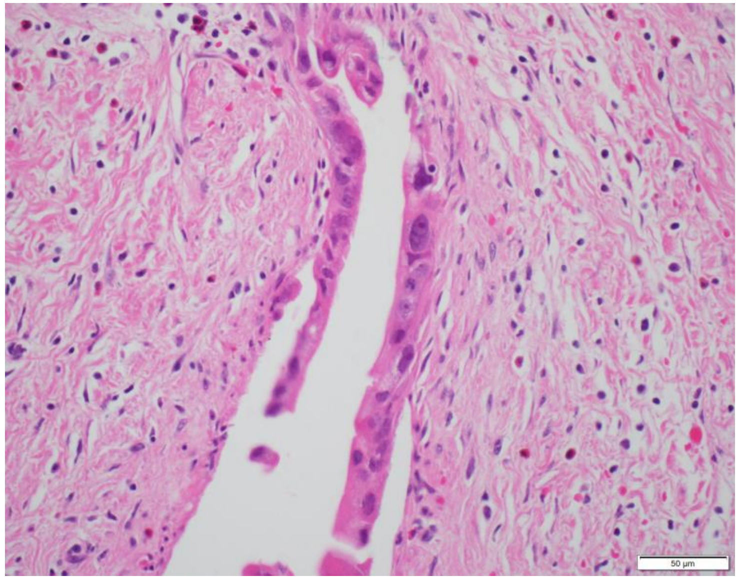

**Patient Number**	P11
**Age**	73
**Gender**	Female
**Stage at Diagnosis**	IV
**Treatment before tissue collection**	No
**Tissue site**	Liver
**Procedure**	Biopsy
**Pathology**	Moderately differentiated adenocarcinoma
**Mutations**	KRAS G12L, TP53 R175H, CDKN2A intron 1 rearrangement

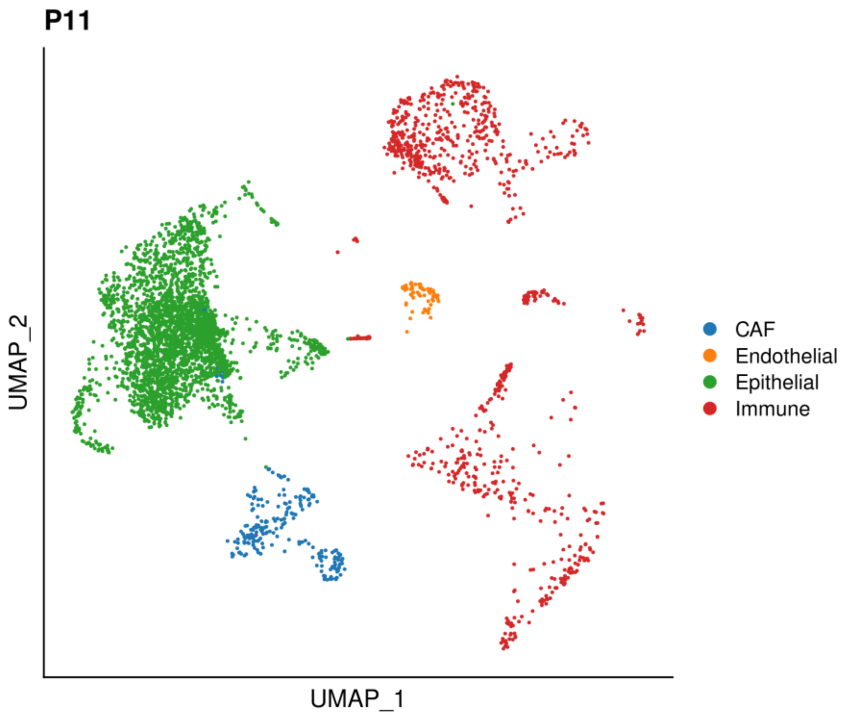

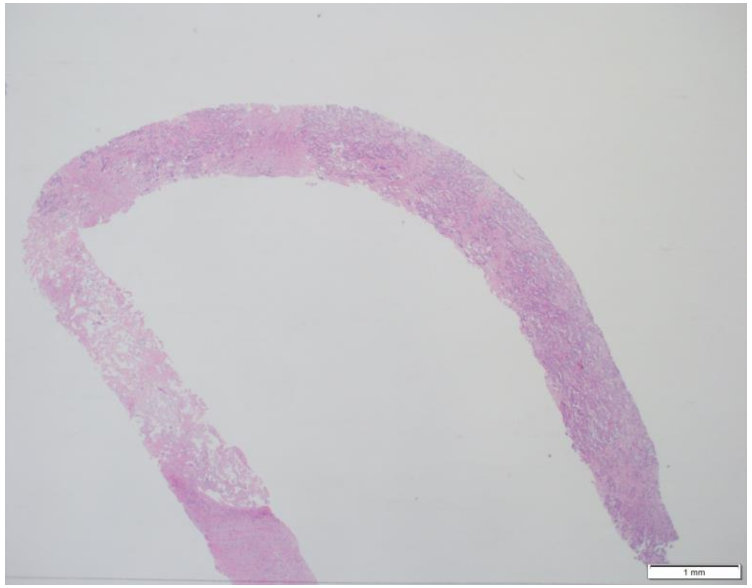

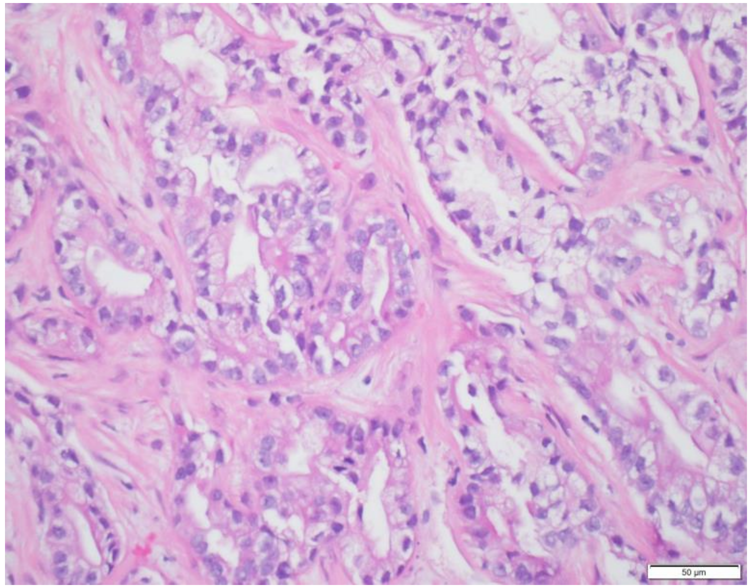

**Patient Number**	P12
**Age**	68
**Gender**	Male
**Stage at Diagnosis**	IV
**Treatment before tissue collection**	Yes
**Therapeutics**	GA
**Tissue site**	Pancreas
**Procedure**	Biopsy
**Pathology**	Moderately differentiated adenocarcinoma
**Mutations**	KRAS G12V, TP53 R306*, SMAD4 R361C, CDKN2A W110*

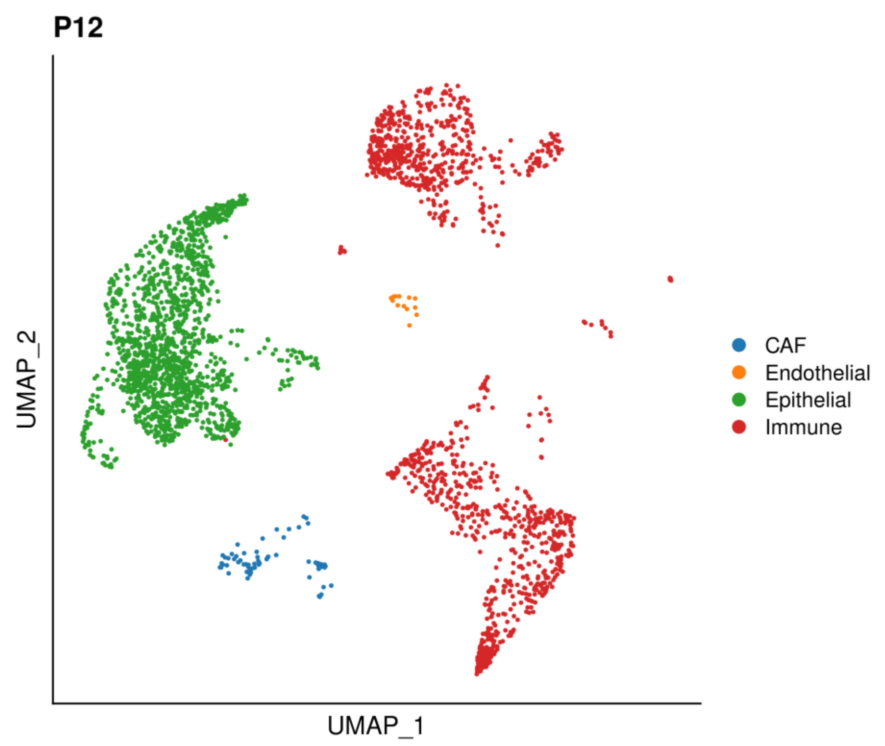

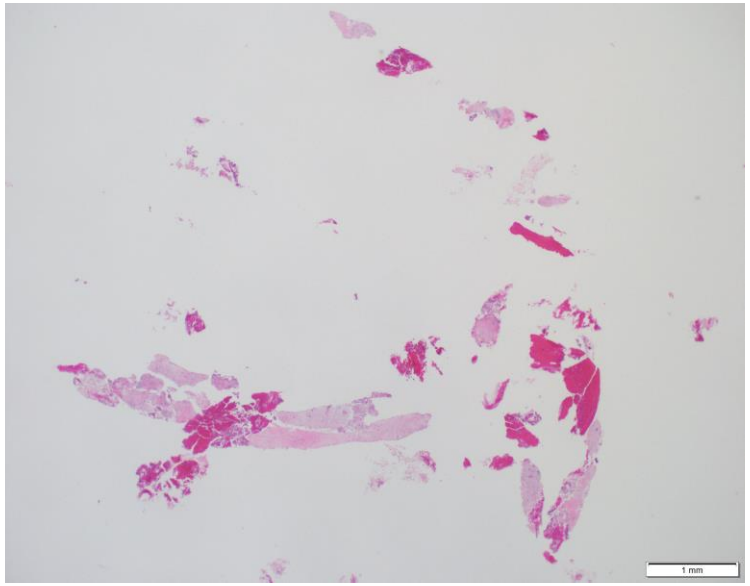

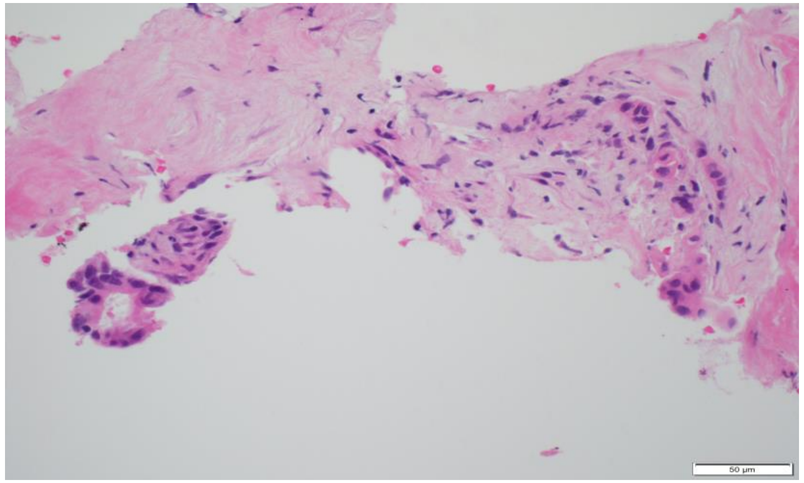

**Patient Number**	P13
**Age**	78
**Gender**	Male
**Stage at Diagnosis**	III
**Treatment before tissue collection**	No
**Tissue site**	Pancreas
**Procedure**	Biopsy
**Pathology**	Poorly differentiated adenocarcinoma
**Mutations**	TP53 c.880del

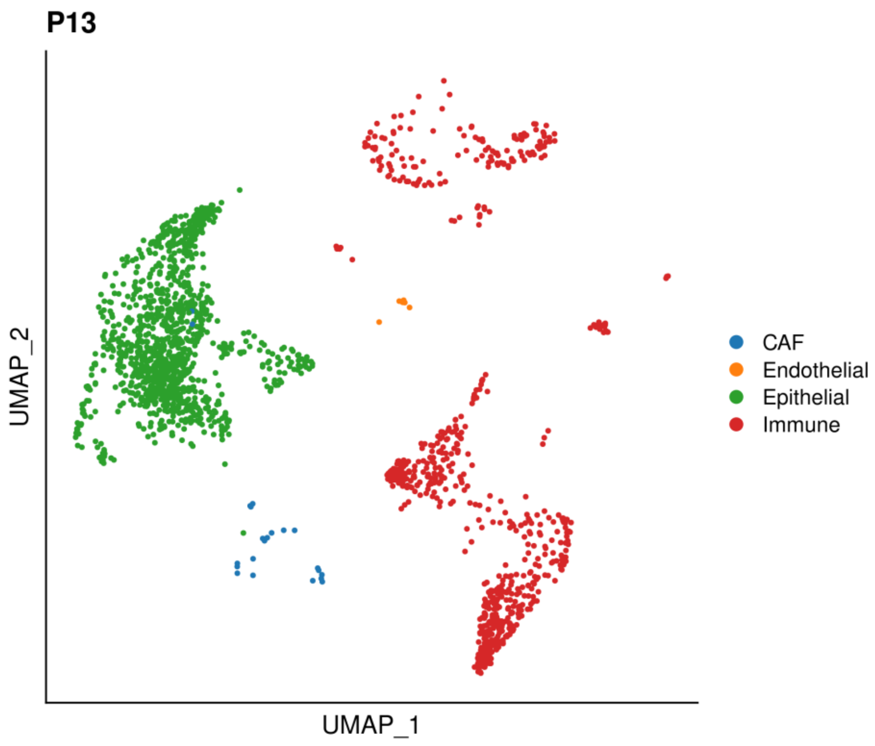

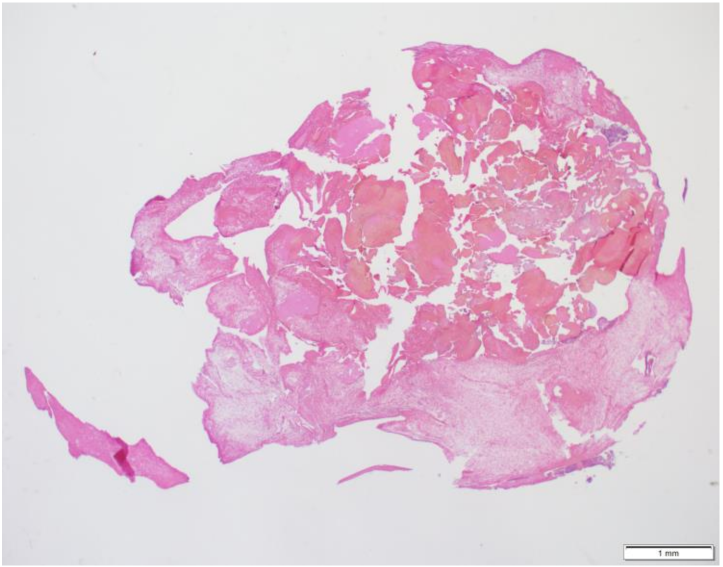

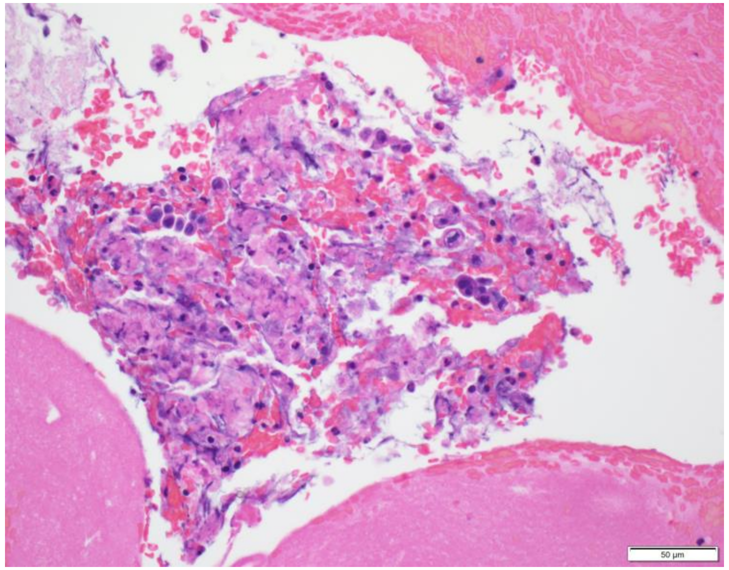

**Patient Number**	P14
**Age**	67
**Gender**	Female
**Stage at Diagnosis**	IB
**Treatment before tissue collection**	Yes
**Therapeutics**	FFX-based
**Tissue site**	Pancreas
**Procedure**	Resection
**Pathology**	Well differentiated adenocarcinoma
**Mutations**	WT

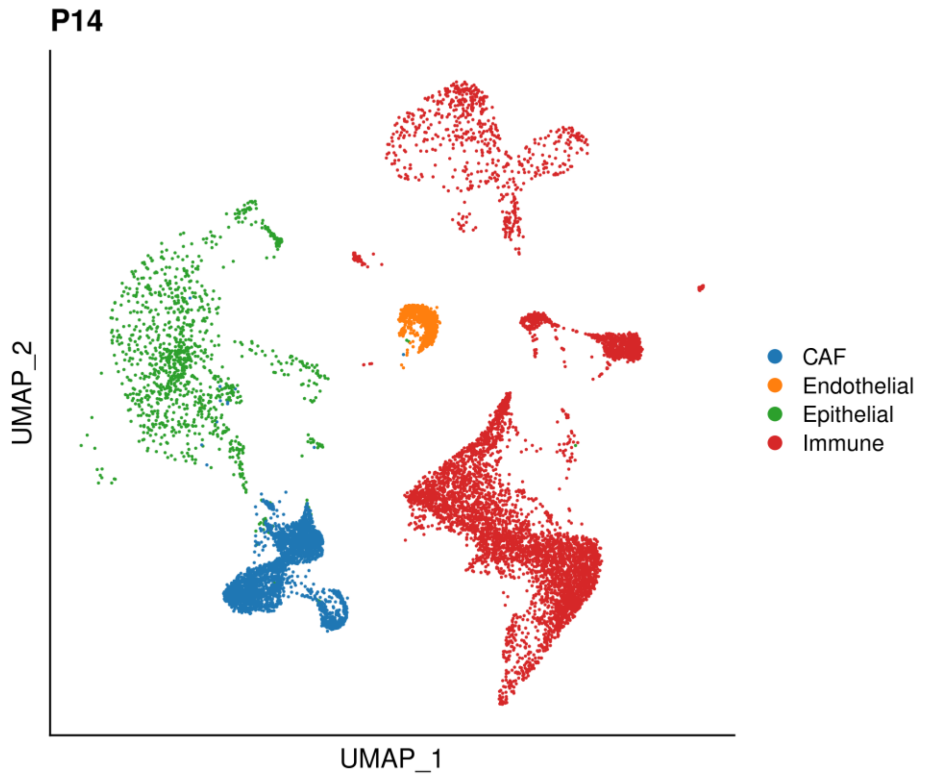

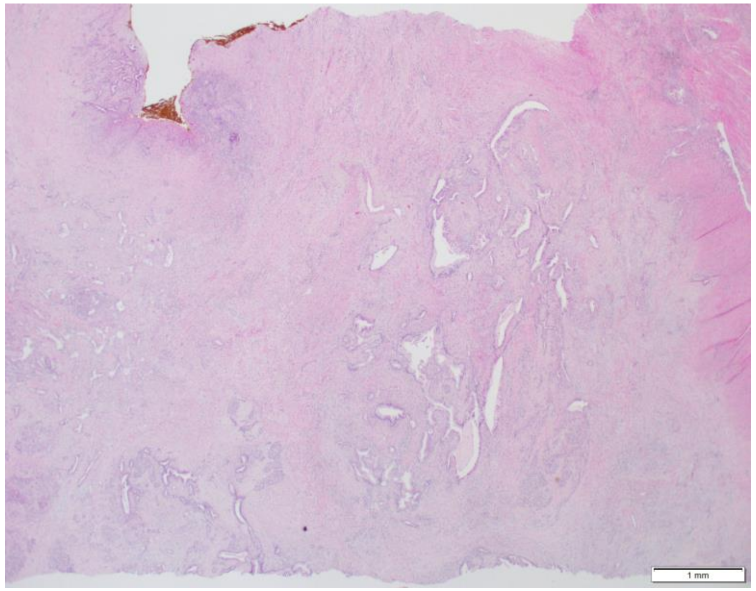

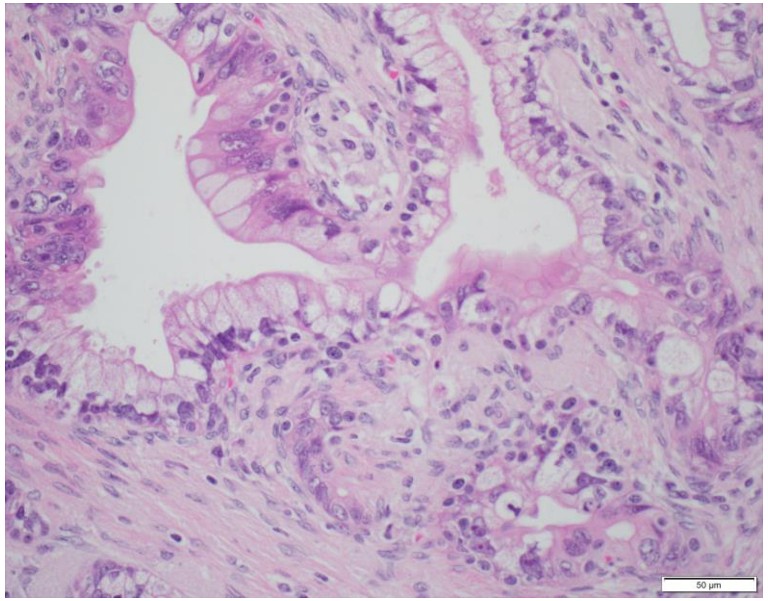

**Patient Number**	P15
**Age**	58
**Gender**	Female
**Stage at Diagnosis**	III
**Treatment before tissue collection**	No
**Tissue site**	Pancreas
**Procedure**	Resection
**Pathology**	Poorly differentiated adenocarcinoma
**Mutations**	KRAS G12V, TP53 C242Y, SMAD4 c.454+1G>A, CDKN2A R80*

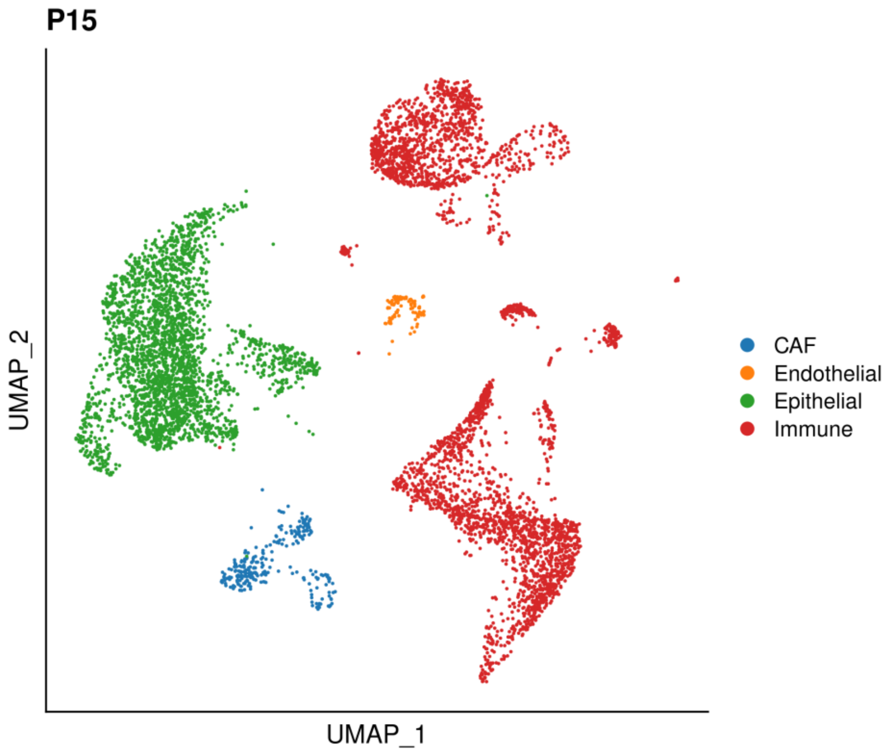

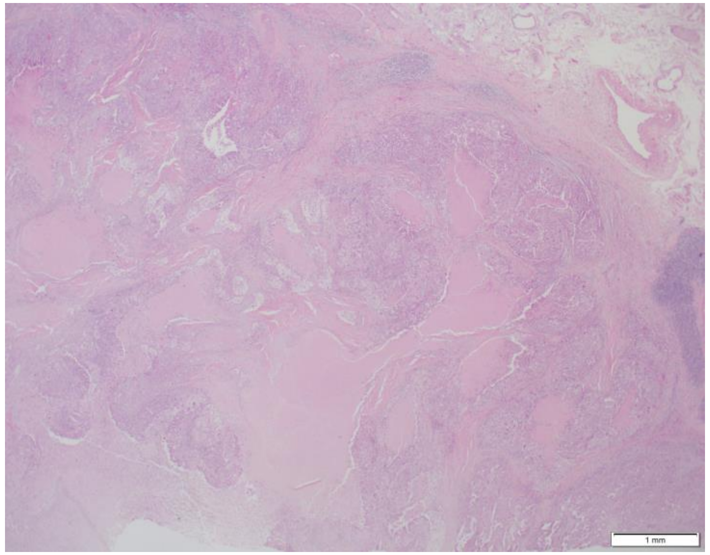

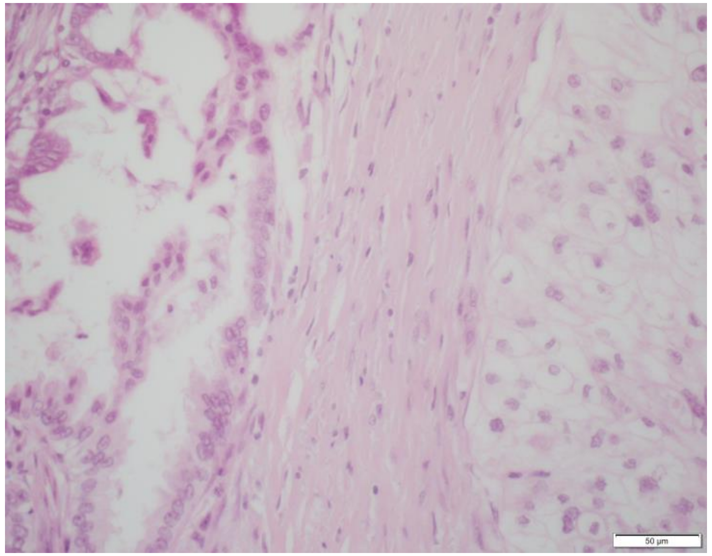

**Patient Number**	P16
**Age**	69
**Gender**	Female
**Stage at Diagnosis**	IV
**Treatment before tissue collection**	No
**Tissue site**	Liver
**Procedure**	Biopsy
**Pathology**	Moderately to poorly differentiated adenocarcinoma
**Mutations**	KRAS G12V, TP53 C135F

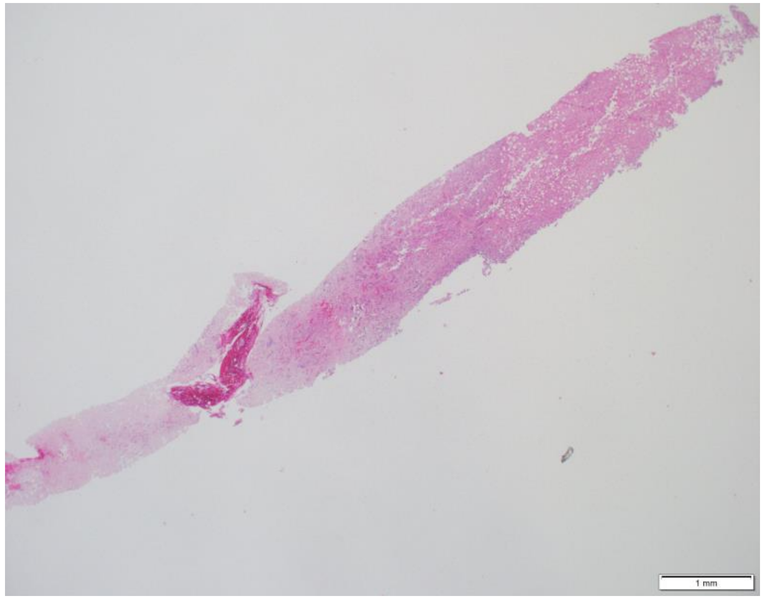

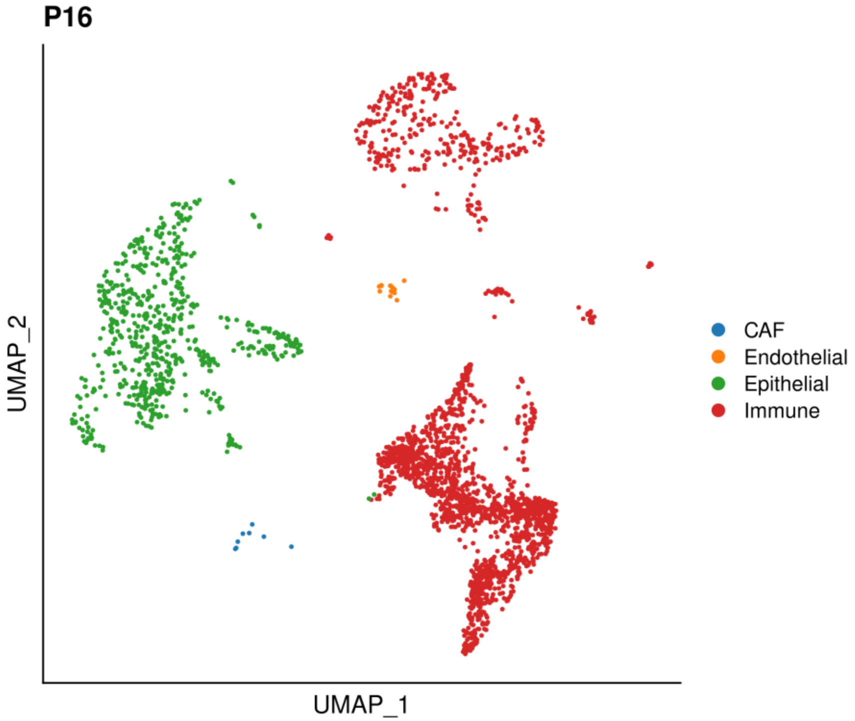

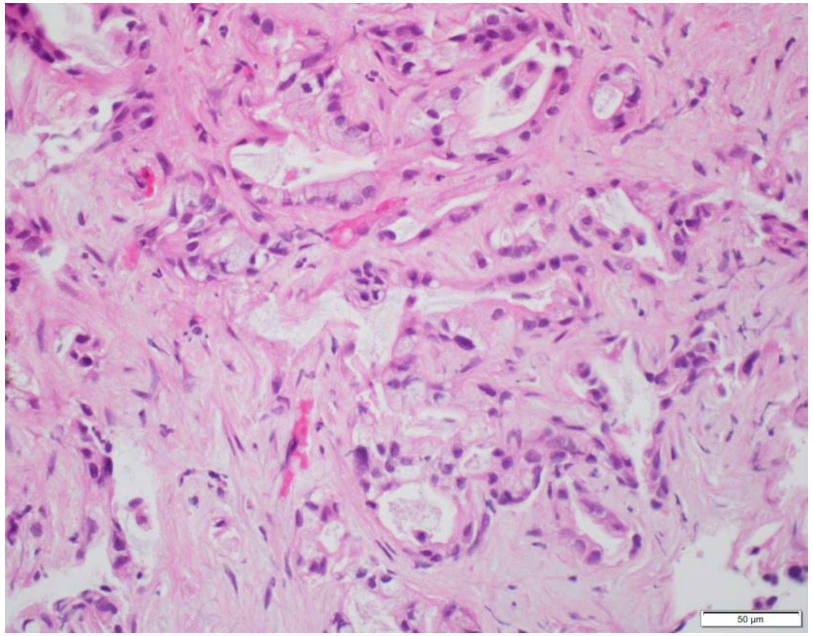

**Patient Number**	P17
**Age**	64
**Gender**	Male
**Stage at Diagnosis**	IV
**Treatment before tissue collection**	Yes
**Therapeutics**	FFX-based
**Tissue site**	Liver
**Procedure**	Biopsy
**Pathology**	Moderately differentiated adenocarcinoma
**Mutations**	N/A

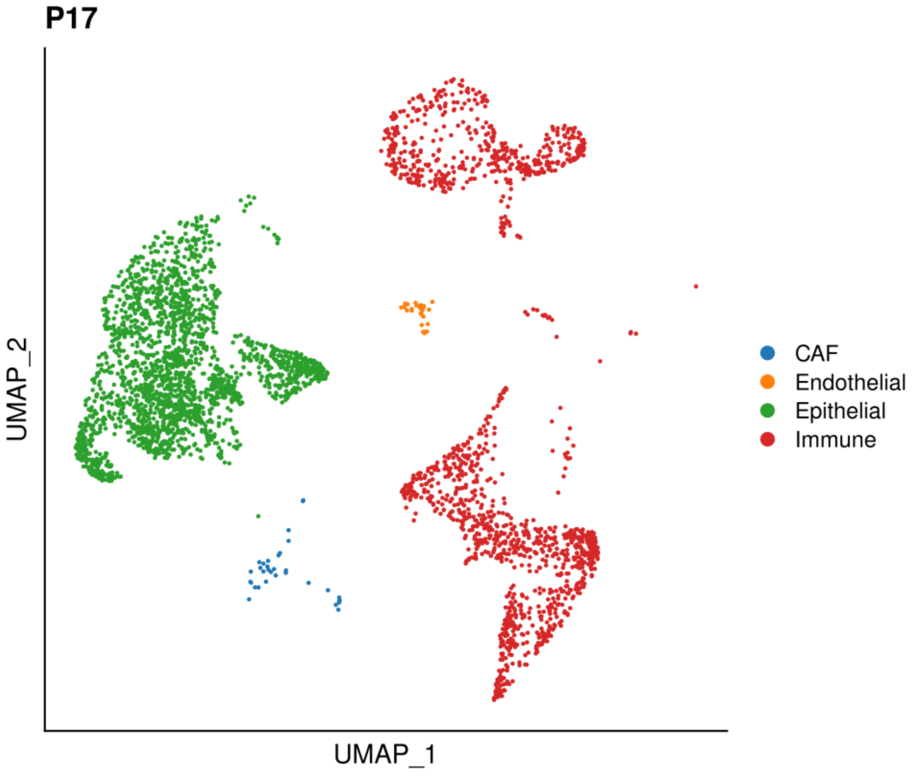

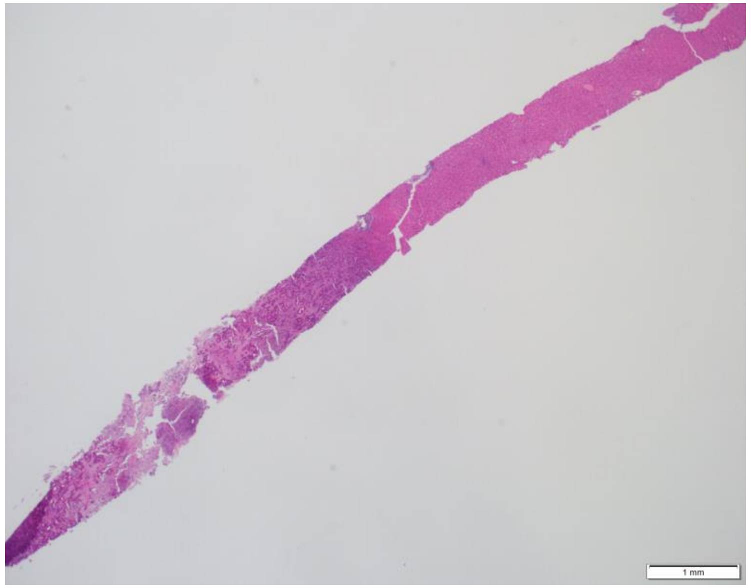

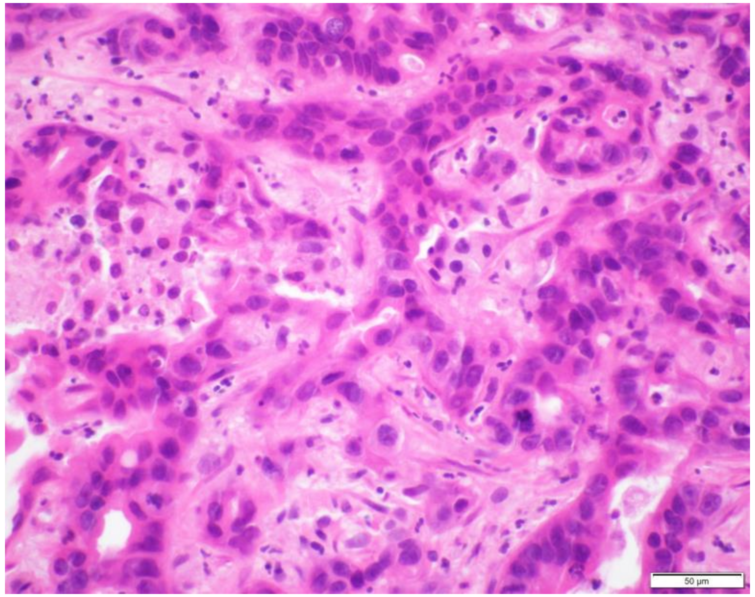

**Patient Number**	P18
**Age**	75
**Gender**	Male
**Stage at Diagnosis**	IV
**Treatment before tissue collection**	No
**Tissue site**	Liver
**Procedure**	Biopsy
**Pathology**	Poorly differentiated adenocarcinoma
**Mutations**	KRAS G12D, TP53 R248Q, CDKN2A loss

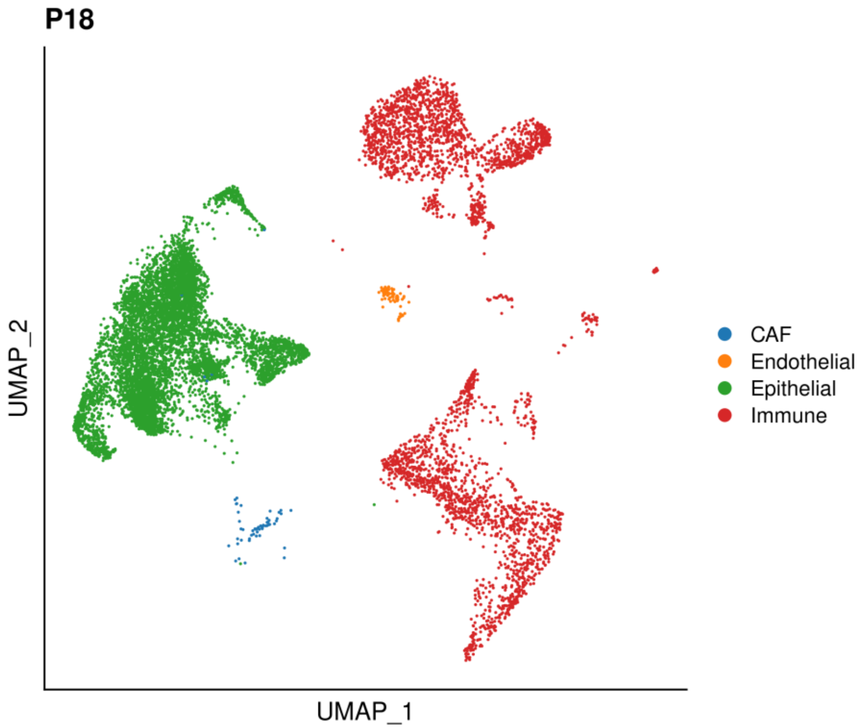

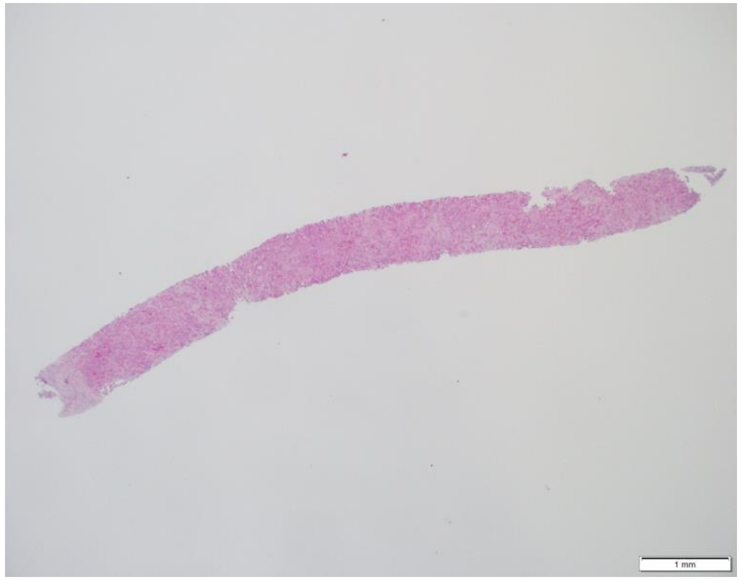

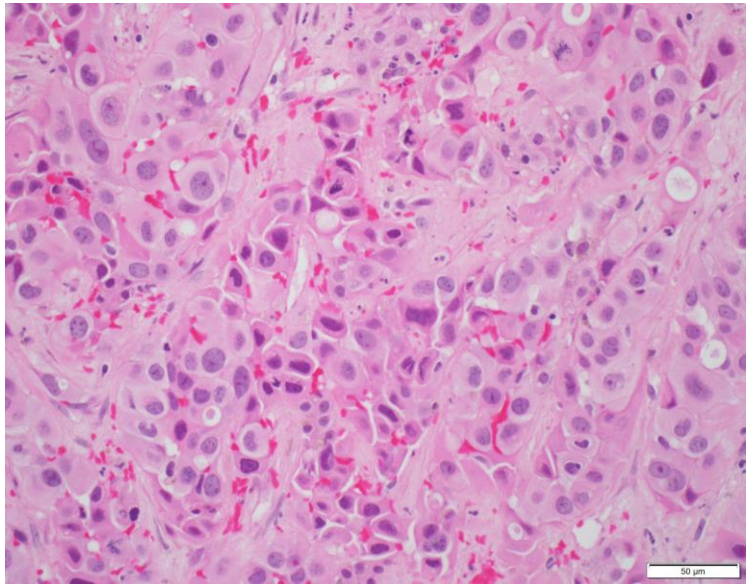

**Patient Number**	P19
**Age**	60
**Gender**	Female
**Stage at Diagnosis**	IB
**Treatment before tissue collection**	No
**Tissue site**	Pancreas
**Procedure**	Resection
**Pathology**	Moderately to poorly differentiated adenocarcinoma
**Mutations**	KRAS G12V, TP53 c.993+1G>A

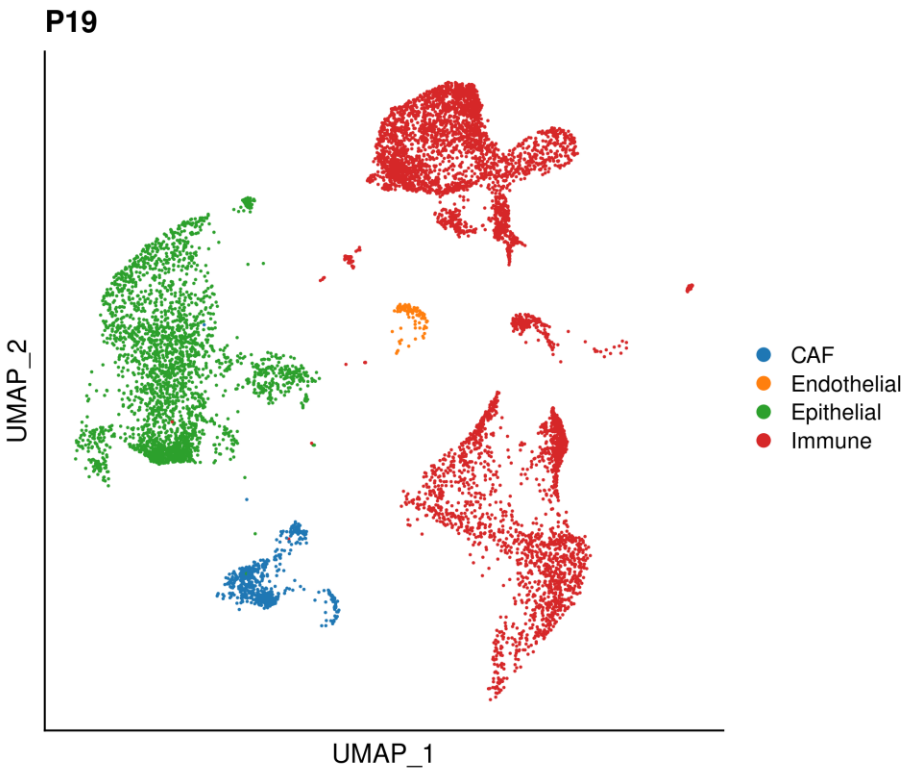

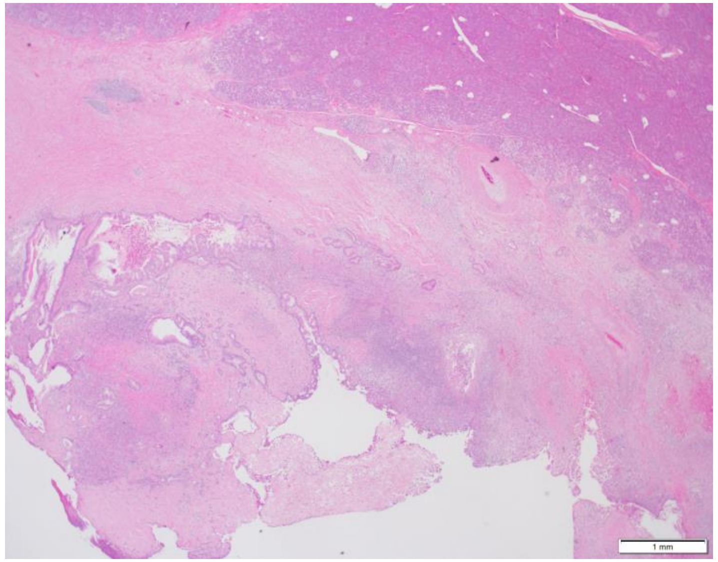

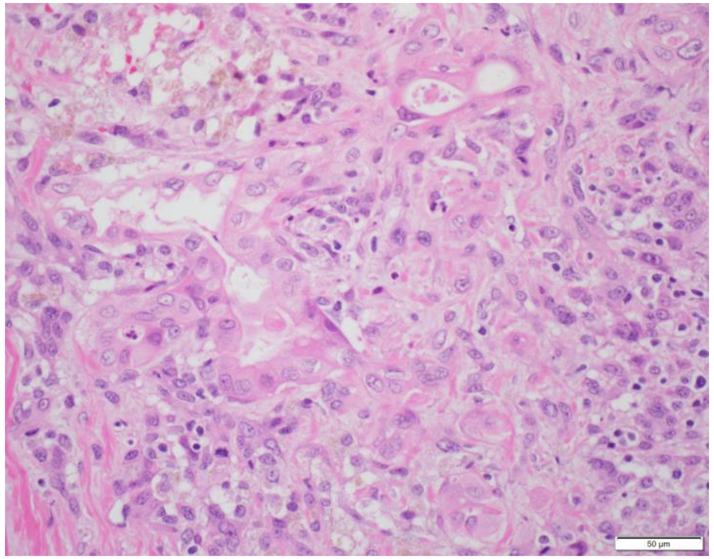

**Patient Number**	P20
**Age**	66
**Gender**	Female
**Stage at Diagnosis**	IV
**Treatment before tissue collection**	No
**Tissue site**	Pancreas
**Procedure**	Biopsy
**Pathology**	Moderately differentiated adenocarcinoma
**Mutations**	KRAS G12D, TP53 R249I

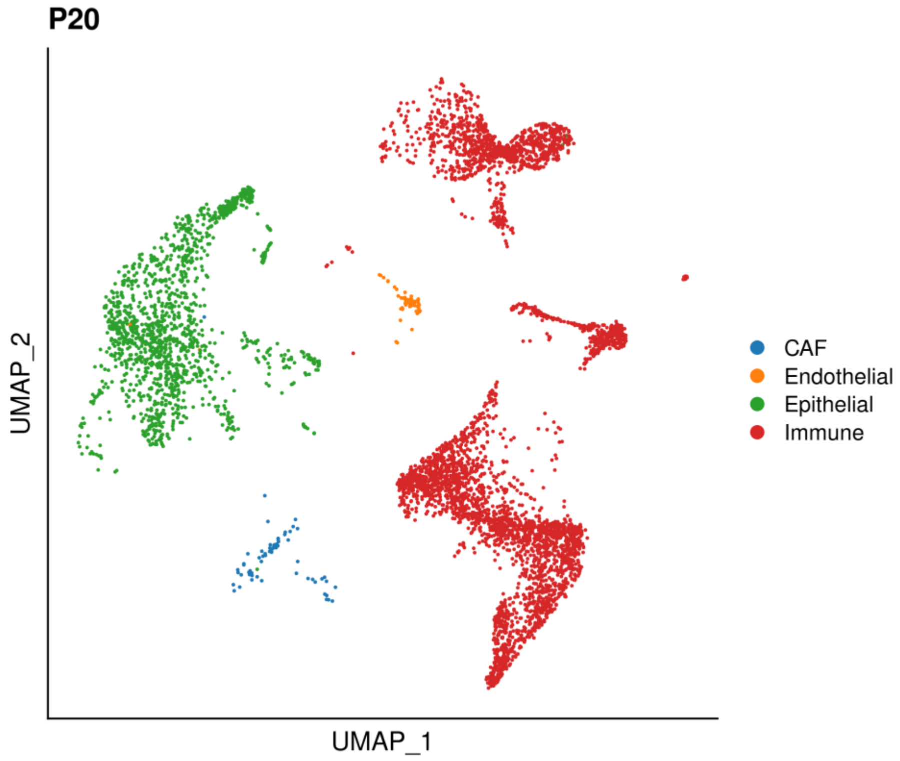

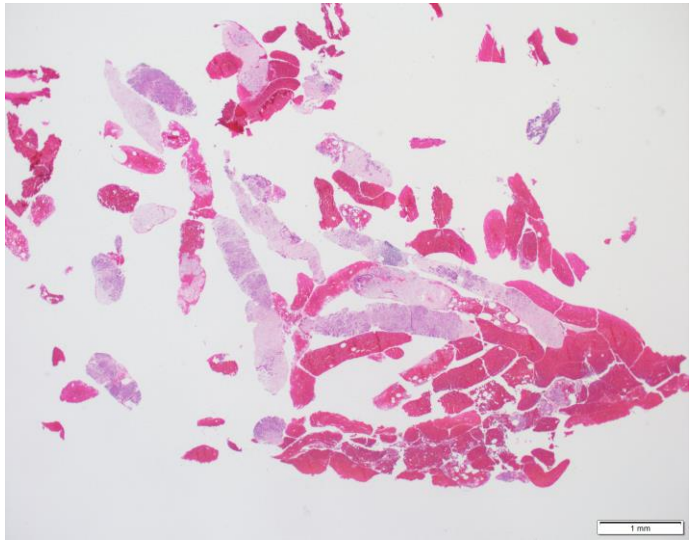

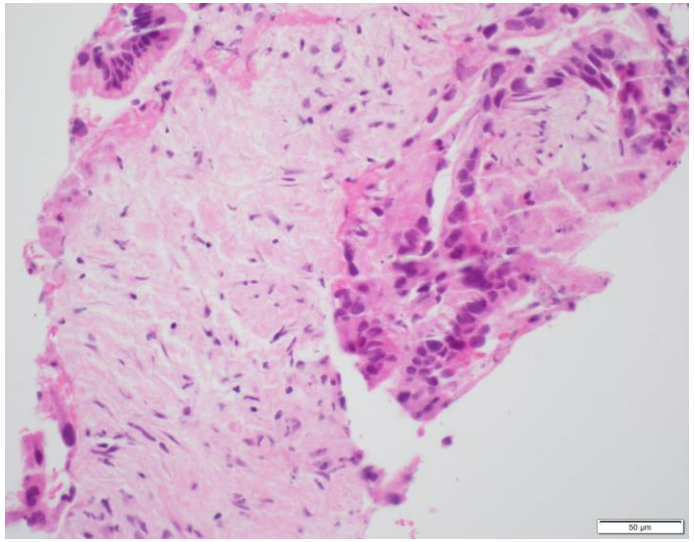

**Patient Number**	P21
**Age**	69
**Gender**	Male
**Stage at Diagnosis**	IV
**Treatment before tissue collection**	No
**Tissue site**	Liver
**Procedure**	Biopsy
**Pathology**	Moderately to poorly differentiated adenocarcinoma
**Mutations**	KRAS G12D, TP53 H168L

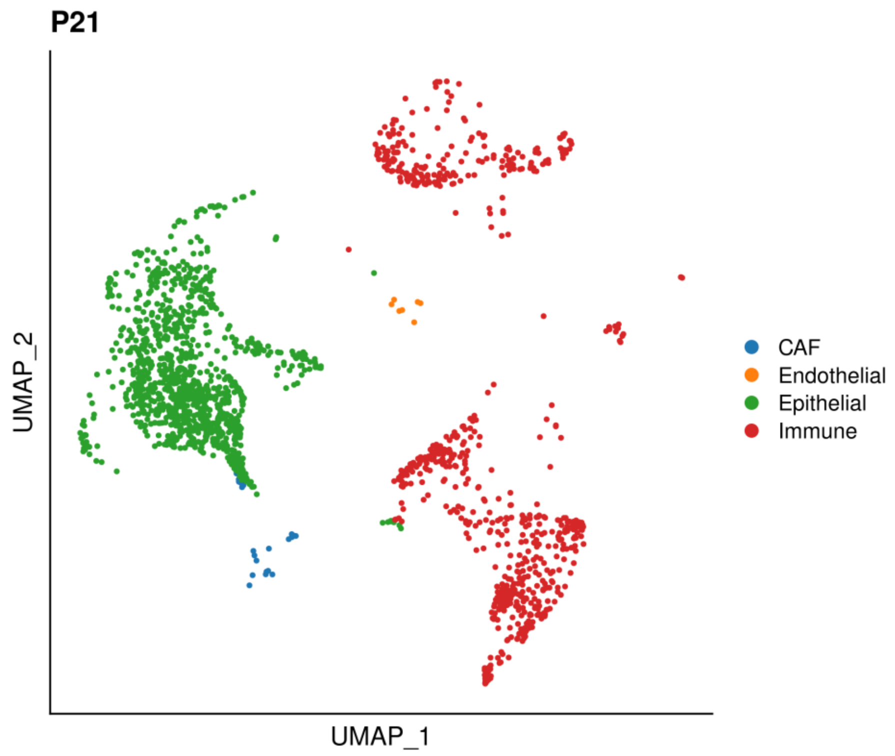

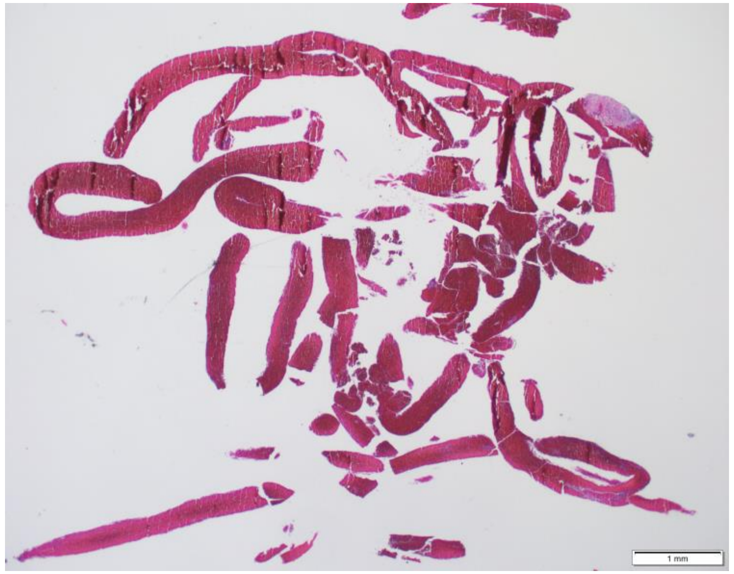

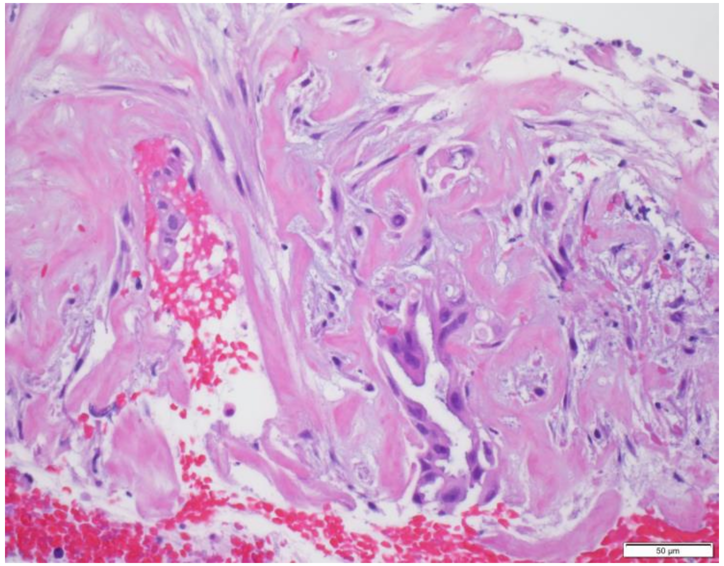

**Patient Number**	P22
**Age**	78
**Gender**	Male
**Stage at Diagnosis**	III
**Treatment before tissue collection**	No
**Tissue site**	Pancreas
**Procedure**	Biopsy
**Pathology**	Moderate differentiated adenocarcinoma
**Mutations**	N/A

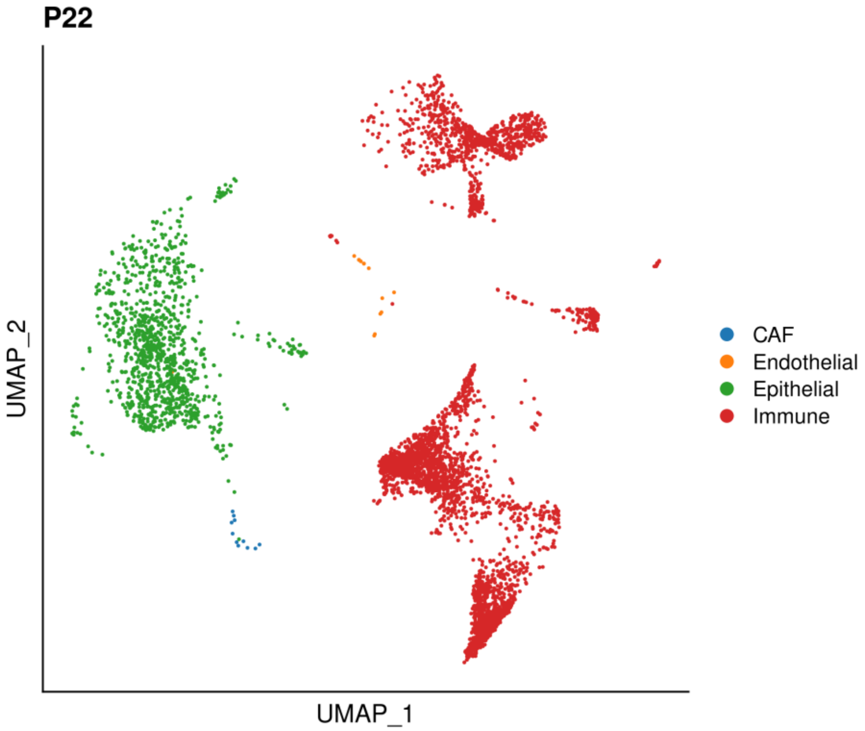

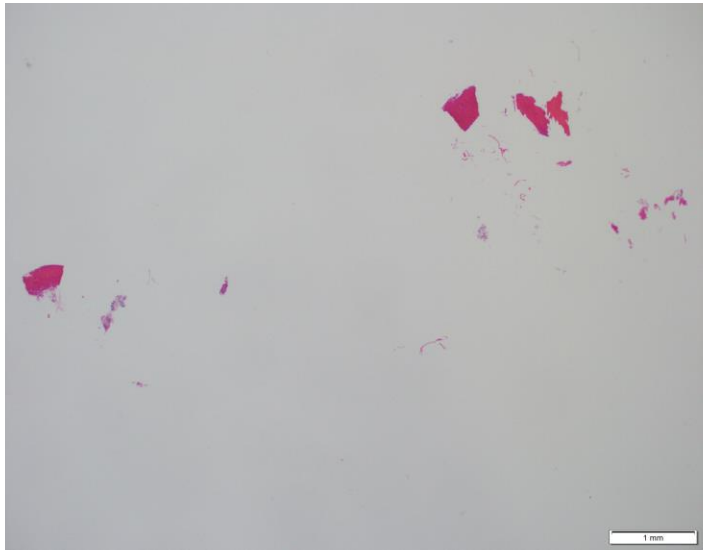

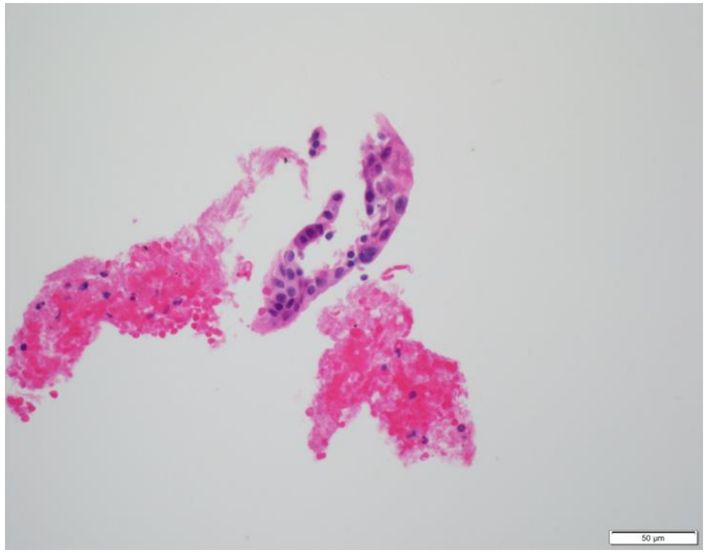

**Patient Number**	P23
**Age**	66
**Gender**	Female
**Stage at Diagnosis**	IB
**Treatment before tissue collection**	No
**Tissue site**	Pancreas
**Procedure**	Resection
**Pathology**	Well to moderately differentiated adenocarcinoma
**Mutations**	KRAS G12D, TP53 I195S

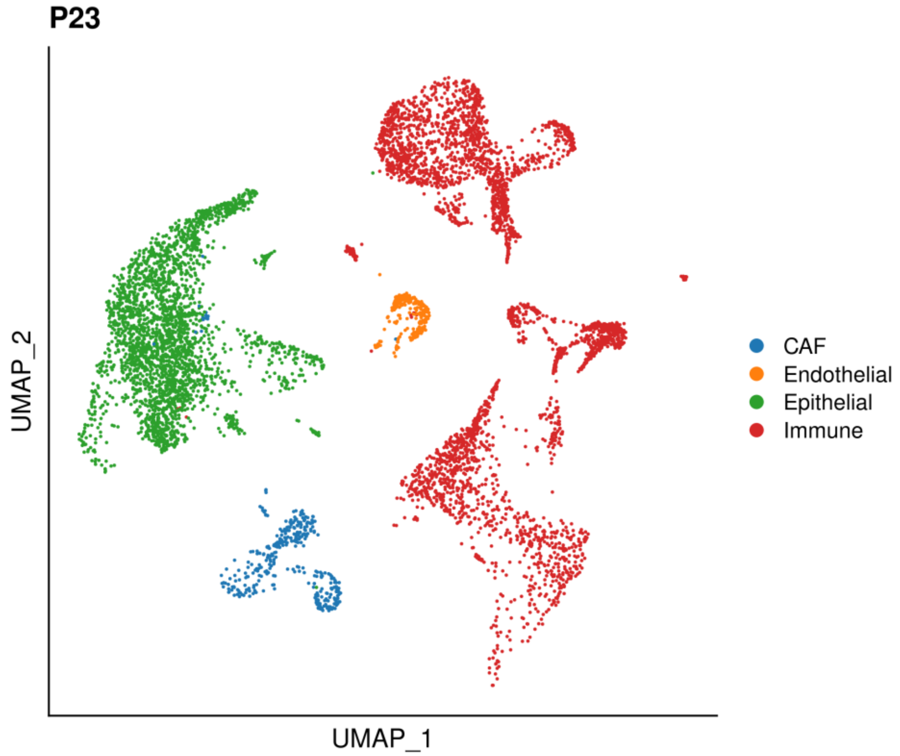

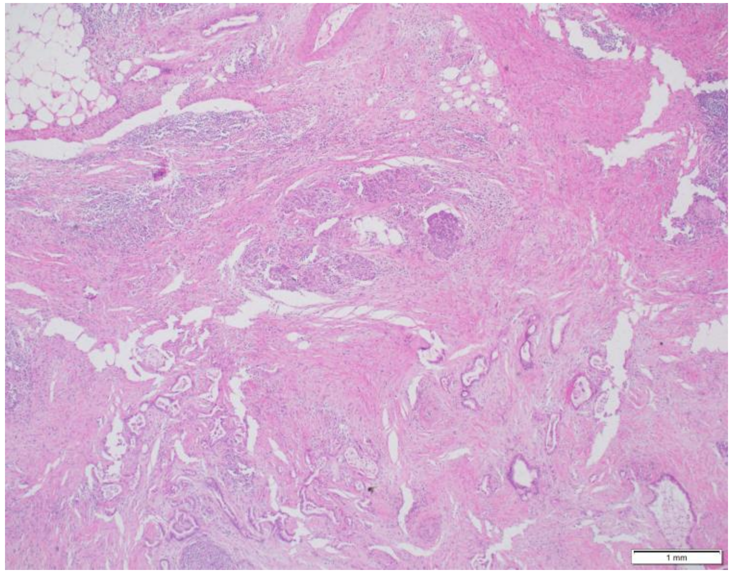

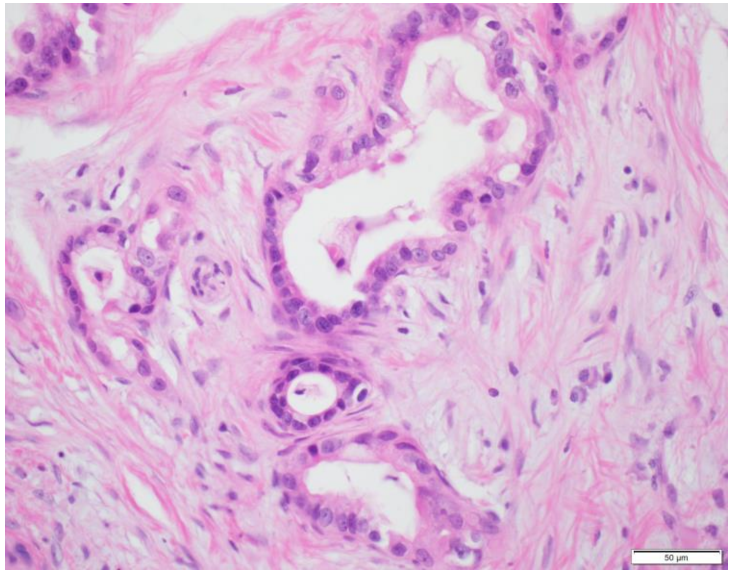

**Patient Number**	P24
**Age**	65
**Gender**	Female
**Stage at Diagnosis**	IV
**Treatment before tissue collection**	No
**Tissue site**	Liver
**Procedure**	Biopsy
**Pathology**	Moderately to poorly differentiated
**Mutations**	KRAS Q61K, TP53 C176Y

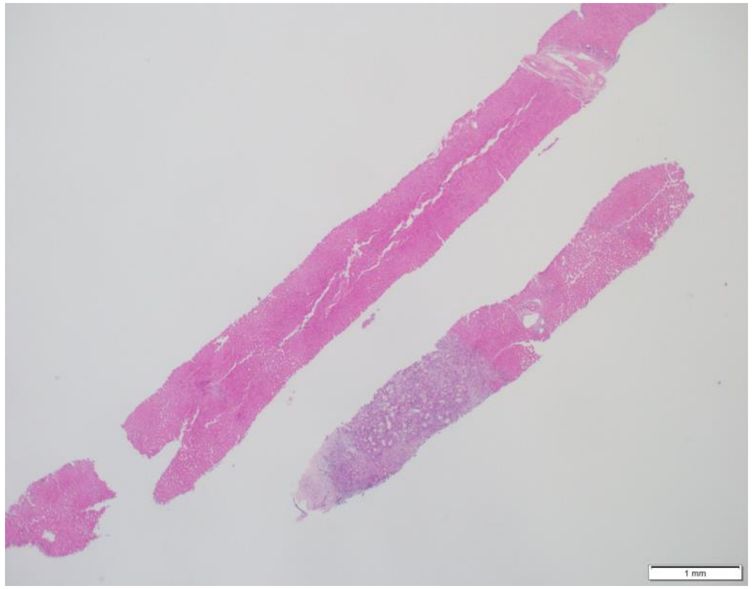

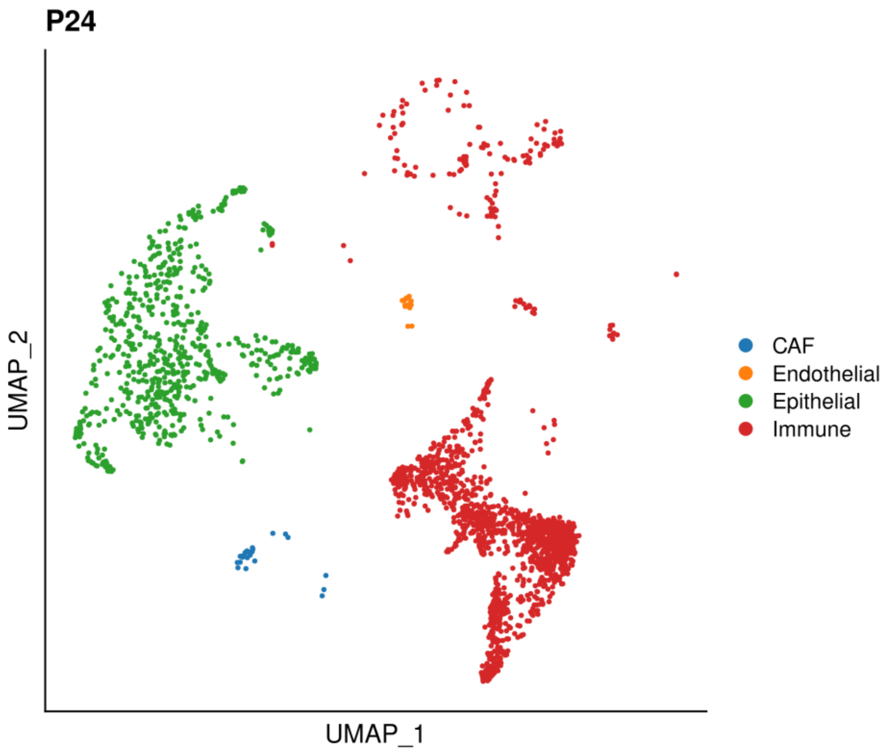

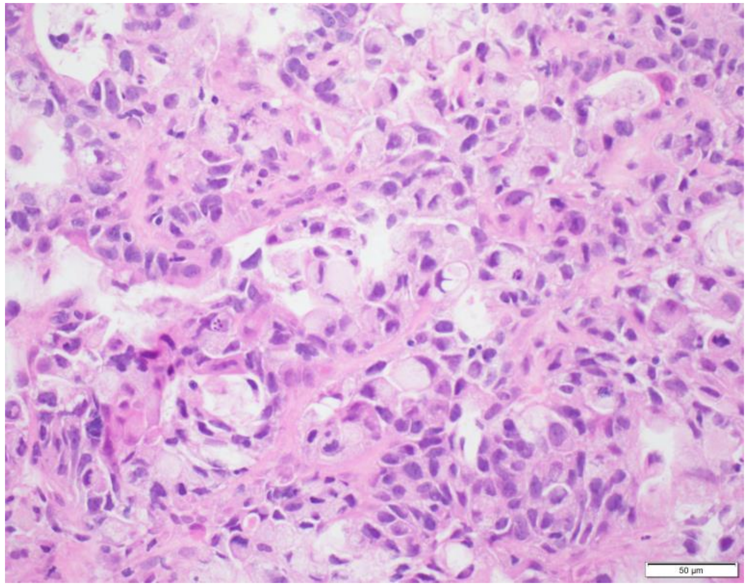

**Patient Number**	P25
**Age**	48
**Gender**	Male
**Stage at Diagnosis**	IV
**Treatment before tissue collection**	No
**Tissue site**	Liver
**Procedure**	Biopsy
**Pathology**	Poorly differentiated adenocarcinoma
**Mutations**	KRAS G12V

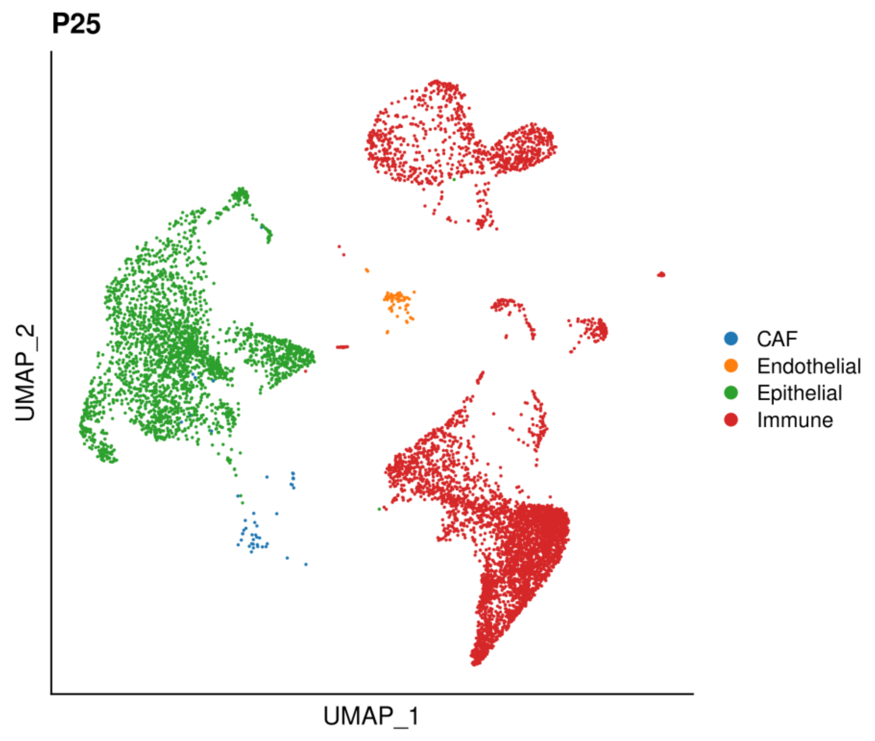

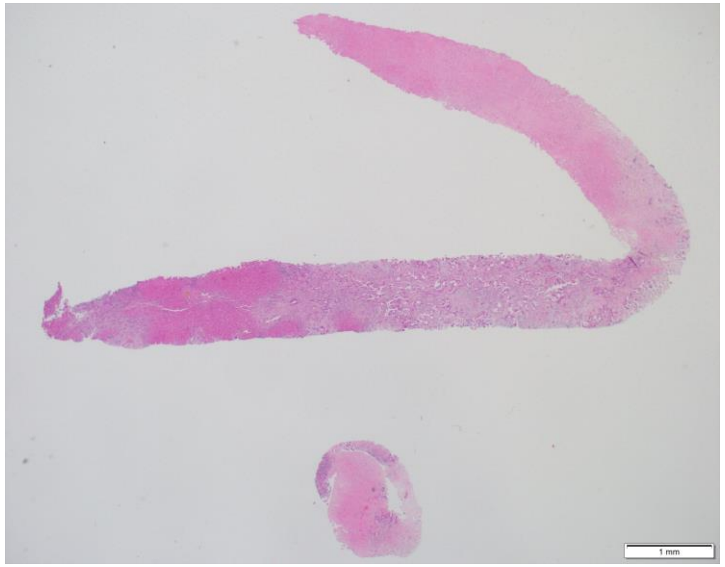

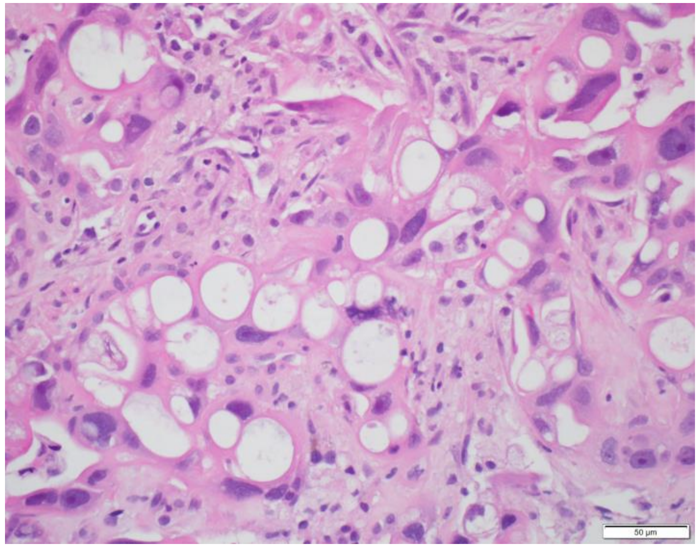

**Patient Number**	P26
**Age**	64
**Gender**	Male
**Stage at Diagnosis**	IV
**Treatment before tissue collection**	No
**Tissue site**	Pancreas
**Procedure**	Biopsy
**Pathology**	Moderately differentiated adenocarcinoma
**Mutations**	KRAS G12V

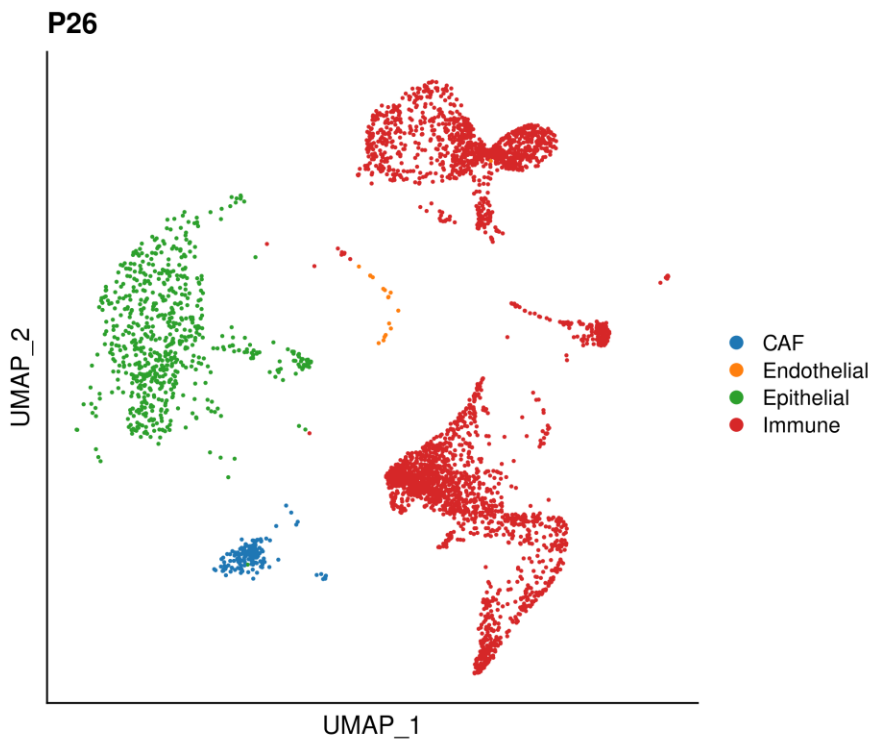

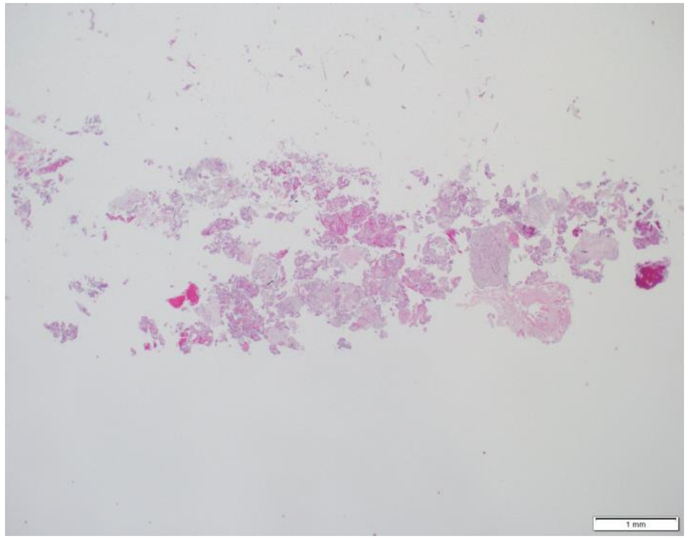

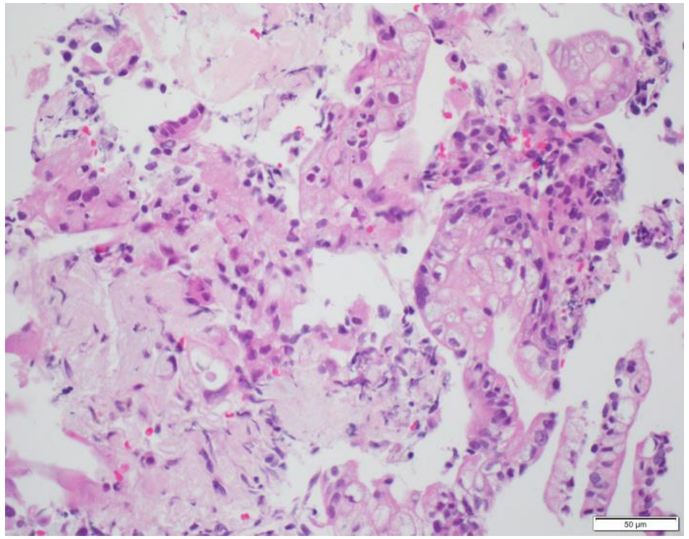

**Patient Number**	P27
**Age**	72
**Gender**	Female
**Stage at Diagnosis**	IV
**Treatment before tissue collection**	No
**Tissue site**	Liver
**Procedure**	Biopsy
**Pathology**	Moderately to poorly differentiated adenocarcinoma
**Mutations**	WT

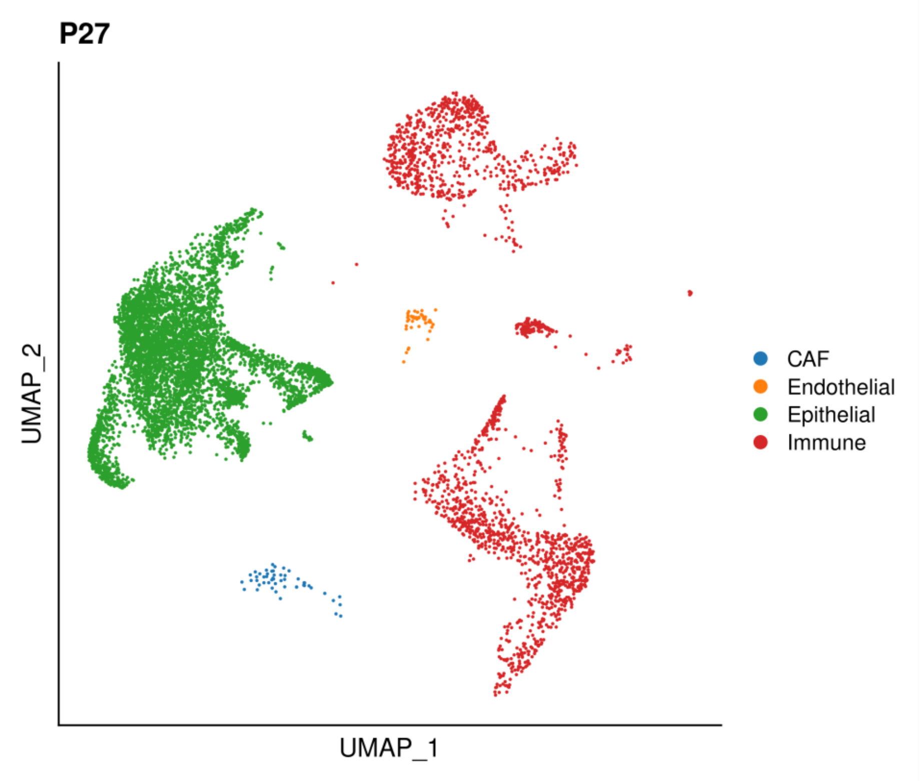

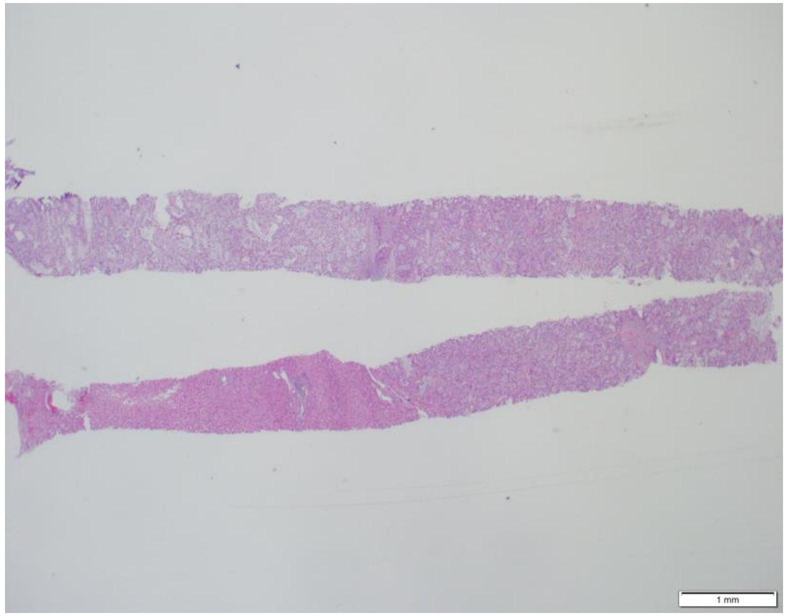

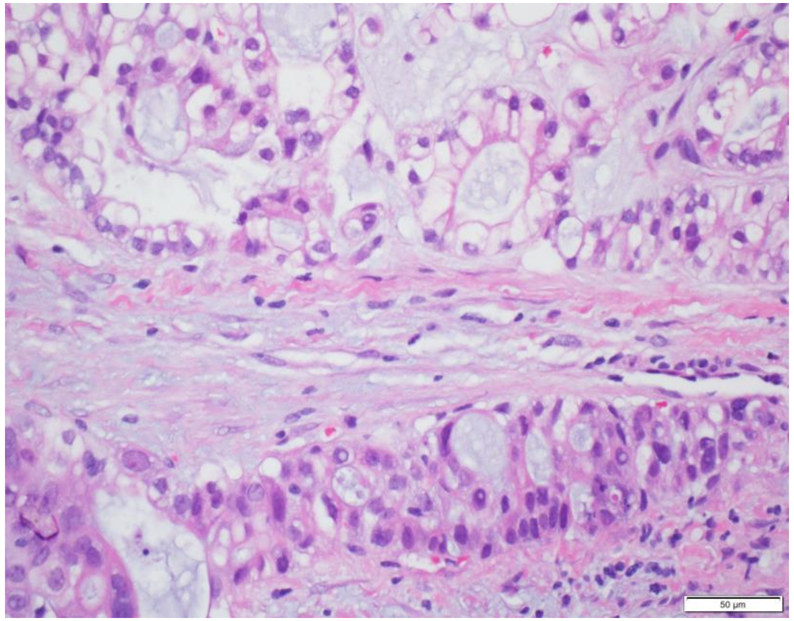

Supplement: Supplementary file 4 — Supplementary Data 2 [file 41467_2023_36296_MOESM4_ESM.pdf]
