# Supplementary material for: Single-cell RNA sequencing reveals the effects of chemotherapy on human pancreatic adenocarcinoma and its tumor microenvironment
Source: Nat Commun. 2023 Feb 13;14:797. doi: 10.1038/s41467-023-36296-4 (PMC9925748; doi:10.1038/s41467-023-36296-4)
Supplement: Supplementary file 8 — Reporting Summary [file 41467_2023_36296_MOESM8_ESM.pdf]

nature portfolio

Corresponding author(s): Diane M. Simeone

Last updated by author(s): Jan 24, 2023

Reporting Summary

Nature Portfolio wishes to improve the reproducibility of the work that we publish. This form provides structure for consistency and transparency in reporting. For further information on Nature Portfolio policies, see our Editorial Policies and the Editorial Policy Checklist.

Statistics

For all statistical analyses, confirm that the following items are present in the figure legend, table legend, main text, or Methods section.

n/a	Confirmed	
☐	☒	The exact sample size (*n*) for each experimental group/condition, given as a discrete number and unit of measurement
☐	☒	A statement on whether measurements were taken from distinct samples or whether the same sample was measured repeatedly
☐	☒	The statistical test(s) used AND whether they are one- or two-sided<br>*Only common tests should be described solely by name; describe more complex techniques in the Methods section.*
☐	☒	A description of all covariates tested
☐	☒	A description of any assumptions or corrections, such as tests of normality and adjustment for multiple comparisons
☐	☒	A full description of the statistical parameters including central tendency (e.g. means) or other basic estimates (e.g. regression coefficient) AND variation (e.g. standard deviation) or associated estimates of uncertainty (e.g. confidence intervals)
☐	☒	For null hypothesis testing, the test statistic (e.g. *F*, *t*, *r*) with confidence intervals, effect sizes, degrees of freedom and *P* value noted<br>*Give P values as exact values whenever suitable.*
☒	☐	For Bayesian analysis, information on the choice of priors and Markov chain Monte Carlo settings
☒	☐	For hierarchical and complex designs, identification of the appropriate level for tests and full reporting of outcomes
☐	☒	Estimates of effect sizes (e.g. Cohen's *d*, Pearson's *r*), indicating how they were calculated

Our web collection on statistics for biologists contains articles on many of the points above.

Software and code

Policy information about availability of computer code

Data collection	TCGABiolinks version 3.15
Data analysis	10x Genomics Cell Ranger 5.0.1<br>Seurat [R] version 4.1.0<br>scooter [R] version 0.0.0.9002<br>SoupX [R] version 1.5.2<br>scDblFinder [R] version 1.6.0<br>InferCNV version 1.8.1<br>CellPhoneDB v.2.1.7<br>GSEA version 4.2.0 [Gene chip annotation file: Human_Gene_Symbol_with_Remapping_MSigDB.v7.4.chip; Genesets: Hallmarks (h.all.v7.5.1), KEGG (c2.cp.kegg.v7.5.1), Reactome (c2.cp.reactome.v7.5.1), Canonical Pathways (c2.cp.v7.5.1), GO (c5.go.v7.5.1)]<br>survminer [R] version 0.4.9<br>survival [R] version 3.3.1<br>glmnet [R] version 4.1.6<br>phenoptrReports [R] version 0.3.2<br>InForm (Akoya Biosciences) version 2.6

For manuscripts utilizing custom algorithms or software that are central to the research but not yet described in published literature, software must be made available to editors and reviewers. We strongly encourage code deposition in a community repository (e.g. GitHub). See the Nature Portfolio guidelines for submitting code & software for further information.

Data

Policy information about availability of data

All manuscripts must include a data availability statement. This statement should provide the following information, where applicable:

- Accession codes, unique identifiers, or web links for publicly available datasets
- A description of any restrictions on data availability
- For clinical datasets or third party data, please ensure that the statement adheres to our policy

scSeq data generated for this project is available on GEO with accession number GSE205013 [https://www.ncbi.nlm.nih.gov/geo/query/acc.cgi?acc=GSE205013]. TCGA data is publicly available on the GDC Data Portal [https://portal.gdc.cancer.gov/]. The confirmatory dataset from Cui Zhou et al. is publicly available at the Human Tumor Atlas Network (HTAN) under the HTAN WUSTL Atlas [https://humantumoratlas.org/explore?selectedFilters=%5B%7B%22group%22%3A%22AtlasName%22%2C%22value%22%3A%22HTAN+WUSTL%22%7D%5D&tab=file] and on dbGaP with accession number phs002371.v1.p1 [https://www.ncbi.nlm.nih.gov/projects/gap/cgi-bin/study.cgi?study_id=phs002371.v2.p1]. The remaining data are available within the article, Supplementary Information, or Source Data file.

Human research participants

Policy information about studies involving human research participants and Sex and Gender in Research.

Reporting on sex and gender	The findings in this study should generally apply to both sexes and sex was not considered in the study design. Sex data was pulled from patient records, de-identified and is given for each sample as part of the supplementary data. Of overall n=27 patients, 12 were female and 15 male. We did not note any differences between the data from female and male patients; as pancreatic cancer is a disease of both sexes, this is not surprising, and no specific sex- or gender-based analyses were performed.
Population characteristics	A total of 27 individual patient samples were collected for this study. Standard clinicopathological variables including sex (female n=12, male n=15), age (average 68±10), tumor site (liver metastases n=10, primary PDAC n=17), disease stage (AJCC: IB n = 6, IIB n = 2, III n = 5, IV n = 14), type of procedure (resection n = 10, biopsy n = 17) and treatment type (treatment naïve n = 20, FOLFIRINOX-based n = 4, Gemcitabine/Abraxane n =3) were collected for each patient as part of our prospective PDAC database.
Recruitment	A total of 27 PDAC patients were recruited for study participation at the Perlmutter Cancer Center at New York University (NYU) Langone Health between May 1, 2020, and June 30, 2021. Informed consent for the collection of blood, tissue, and clinical information was obtained from all patients using an institutional review board-approved study protocol.
Ethics oversight	The NYU Langone Health Institutional Review Board approved the study protocol.

Note that full information on the approval of the study protocol must also be provided in the manuscript.

Field-specific reporting

Please select the one below that is the best fit for your research. If you are not sure, read the appropriate sections before making your selection.

☒ Life sciences ☐ Behavioural & social sciences ☐ Ecological, evolutionary & environmental sciences

For a reference copy of the document with all sections, see nature.com/documents/nr-reporting-summary-flat.pdf

Life sciences study design

All studies must disclose on these points even when the disclosure is negative.

Sample size	We used all samples with viable scRNA-seq data that were collected within the time period. We believe the 27 samples are enough to form the conclusions presented in the manuscript.
Data exclusions	None
Replication	All results should be replicable following the methods described.
Randomization	No randomization was performed in this study.
Blinding	No blinding was performed in this study.

Reporting for specific materials, systems and methods

We require information from authors about some types of materials, experimental systems and methods used in many studies. Here, indicate whether each material, system or method listed is relevant to your study. If you are not sure if a list item applies to your research, read the appropriate section before selecting a response.

Materials & experimental systems

n/a	Involved in the study
☐	☒ Antibodies
☒	☐ Eukaryotic cell lines
☒	☐ Palaeontology and archaeology
☒	☐ Animals and other organisms
☐	☒ Clinical data
☒	☐ Dual use research of concern

Methods

n/a	Involved in the study
☒	☐ ChIP-seq
☒	☐ Flow cytometry
☒	☐ MRI-based neuroimaging

Antibodies

Antibodies used	In this study the following antibodies were used to stain human tissue (see also Supplementary Table 3):<br>Primary antibodies: GATA6, Vendor: R&D, Cat#: AF1700, Clone: Poly, Dilution: 1:200; CK17, Vendor: Proteintech, Cat#: 17516-1-AP, Clone: Poly, Dilution: 1:100; CK19, Vendor: Biocare Med, Cat#: CD242A, Clone: Ks19.1, Dilution: 1:100; CD8, Vendor: Dako, Cat#: M710301-2/M7103, Clone: C8/144B, Dilution: 1:100; TIGIT, Vendor: Abcam, Cat#: ab243903, Clone: BLR047F, Dilution: 1:200; PD-1, Vendor: CST, Cat#: 86163S, Clone: D4W2J, Dilution: 1:200; PVR, Vendor: CST, Cat#: 81254S, Clone: D8A5G, Dilution: 1:300; TFF3 (ITF), Vendor: Santa Cruz, Cat#: sc-398651, Clone: B-1, Dilution: 1:400<br>Secondary antibodies: 2HRP-polymer: Biocare Med, GHP516, Fluorophore: Opal 480, Vendor: Akoya, Cat#: FP1500001KT; 2HRP-polymer: Akoya, ARH1001, Fluorophore: Opal 520, Vendor: Akoya, Cat#: FP1487001KT; 2HRP-polymer: Akoya, ARH1001, Fluorophore: Opal 780, Vendor: Akoya, Cat#: FP1501001KT TSA-DIG & OP780; 2HRP-polymer: Akoya, ARH1001, Fluorophore: Opal 620, Vendor: Akoya, Cat#: FP1495001KT; 2HRP-polymer: Akoya, ARH1001, Fluorophore: Opal 690, Vendor: Akoya, Cat#: FP1497001KT
Validation	For multiplex immunofluorescence and imaging, five-micron formalin fixed paraffin-embedded sections were stained with Akoya Biosciences® Opal™ multiplex automation kit reagents (Leica Cat #ARD1001EA) on a Leica BondRX® autostainer, according to the manufacturers' instructions. All staining were done at our Experimental Pathology Core Facility. Prior to multiplex co-staining each antibody was internally validated by single immunohistochemistry staining on human PDAC slides and human tonsil tissue. In addition the multiplex panel was tested on human PDAC slides prior to staining of samples included in this study. Staining was evaluated by a board certified pathologist. Additional information on manufacturer's validation, manufacturer's datasheet and relevant citations are available for each antibody on the Vendor's/manufacturer's website using the Cat# provided above.

Clinical data

Policy information about clinical studies

All manuscripts should comply with the ICMJE guidelines for publication of clinical research and a completed CONSORT checklist must be included with all submissions.

Clinical trial registration	None
Study protocol	Because this is a non-therapeutic research trial sponsored by our institution, the research study protocol is confidential within our IRB.
Data collection	A total of 27 PDAC patients were recruited for study participation at the Perlmutter Cancer Center at New York University (NYU) Langone Health between May 1, 2020, and June 30, 2021.
Outcomes	None
